# Integrative Analysis of the microRNAome and Transcriptome Illuminates the Response of Susceptible Rice Plants to Rice Stripe Virus

**DOI:** 10.1371/journal.pone.0146946

**Published:** 2016-01-22

**Authors:** Jian Yang, Fen Zhang, Jing Li, Jian-Ping Chen, Heng-Mu Zhang

**Affiliations:** 1 State Key Laboratory Breeding Base for Zhejiang Sustainable Pest and Disease Control, Key Laboratory of Biotechnology in Plant Protection of MOA and Zhejiang Province, Institute of Virology and Biotechnology, Zhejiang Academy of Agricultural Sciences, Hangzhou, 310021, China; 2 College of Chemistry and Life Science, Zhejiang Normal University, Jinhua, 321004, China; National University of Singapore, SINGAPORE

## Abstract

Rice stripe virus (RSV) is one of the most serious rice viruses in East Asia. To investigate how rice responds to RSV infection, we integrated miRNA expression with parallel mRNA transcription profiling by deep sequencing. A total of 570 miRNAs were identified of which 69 miRNAs (56 up-regulated and 13 down-regulated) were significantly modified by RSV infection. Digital gene expression (DGE) analysis showed that 1274 mRNAs (431 up-regulated and 843 down-regulated genes) were differentially expressed as a result of RSV infection. The differential expression of selected miRNAs and mRNAs was confirmed by qRT-PCR. Gene ontology (GO) and pathway enrichment analysis showed that a complex set of miRNA and mRNA networks were selectively regulated by RSV infection. In particular, 63 differentially expressed miRNAs were found to be significantly and negatively correlated with 160 target mRNAs. Interestingly, 22 up-regulated miRNAs were negatively correlated with 24 down-regulated mRNAs encoding disease resistance-related proteins, indicating that the host defense responses were selectively suppressed by RSV infection. The suppression of both osa-miR1423-5p- and osa-miR1870-5p-mediated resistance pathways was further confirmed by qRT-PCR. Chloroplast functions were also targeted by RSV, especially the zeaxanthin cycle, which would affect the stability of thylakoid membranes and the biosynthesis of ABA. All these modifications may contribute to viral symptom development and provide new insights into the pathogenicity mechanisms of RSV.

## Introduction

Rice is not only a staple cereal but also a model monocotyledonous plant, for which much genomic and related expression sequence tag (EST) information is available. Its production is threatened by at least 15 virus or virus-like diseases [1], one of the most serious of which is rice stripe disease. This is caused by rice stripe virus (RSV) which is transmitted by the small brown planthopper, *Laodelphax striatellus* Fallén, in a persistent and transovarial propagative manner [2] and which can infect many different species of plants in the family Gramineae [3, 4]. The disease was first reported in the central part of Japan at the end of 19th century, and caused more than 50% losses in rice production in some fields [5, 6]. The disease now occurs throughout the major rice-growing areas of East Asia with major epidemics in both Japan and China in recent years, resulting in large yield losses [1, 7–9]. RSV is the type member of the genus *Tenuivirus* [10]. Its virions appear to be fine filaments [10] and its genome comprises four single-stranded RNAs. The largest genomic RNA (RNA1) has a single large open reading frame (ORF) on the viral complementary strand, while all other segments are bicistronic, each with one ORF transcribed from the viral RNA and one ORF from the complementary strand [7, 10].

Many host factors are known to be involved in the response of plants to viruses [11]. Experiments have shown that RSV infection selectively modifies gene expression and induces novel microRNAs (miRNAs) [12–14]. Comparative analysis of transcript data from resistant and susceptible rice plants revealed a broad list of potential host factors associated with resistance to RSV [15, 16]. A major RSV-resistance quantitative trait locus (QTL), qSTV11, was cloned from an indica cultivar and encodes a sulfotransferase (OsSOT1) that catalyzes the conversion of salicylic acid (SA) to sulphonated SA (SSA), leading to SA accumulation and inhibition of viral replication in RSV-infected plants [17]. Such studies are important for developing new strategies for disease control. Nevertheless, our knowledge of the plant response to RSV infection is still very limited, and needs further investigation to explore the mechanisms behind the appearance of disease symptoms and the virus resistance or immunity processes. Deep-sequencing is now changing the way that we understand viruses and its use has already had a substantial impact on molecular virology [18]. Here we used a deep sequencing approach to examine global changes induced by RSV infection in the expression patterns of miRNAs and mRNAs in susceptible rice plants.

## Materials and Methods

### Virus, insect vector and rice samples

Viruliferous small brown planthoppers (*Laodelphax striatellus*) were raised on rice plants (cv. Wuyujing 3) in a growth room. This cultivar is susceptible to RSV [19] and carries the susceptibility STV11-S allele [17]. Viruliferous adult insects were then transferred onto healthy rice seedlings at the three-leaf stage, the stage at which they are most susceptible [8]. Control seedlings received non-viruliferous planthoppers. After 72 h, the planthoppers were removed, and the inoculated seedlings were maintained under insect-proof conditions. One week after inoculation (sufficient for systemic infection to be routinely established in this cultivar), infection was confirmed by ELISA and RT-PCR specific for RSV. Leaves were then collected from the infected and control (mock-inoculated) plants, frozen and stored at -80°C.

### RNA extraction

Total RNA samples were extracted from infected and mock plants using Trizol agent (Invitrogen). The total RNA concentration was examined with a spectrophotometer (Nanodrop ND-2000, ThermoFisher Scientific, Wilmington, DE, USA), and the RNA sample integrity was verified by a Bio-Analyzer 2100 (Agilent Technologies, Waldbronn, Germany).

### Digital gene expression (DGE) sequencing of mRNA and statistical analysis

DGE libraries were prepared using the Illumina gene expression sample prep kit. Briefly, poly(A) RNA purified with oligo(dT) magnetic beads was used to synthesize double-stranded cDNAs and then digested with NlaIII and ligated to a first adapter (adapter 1: 5’-ACACTCTTTCCCTACACGACGCTCTTCCGATC-3’) containing a restriction site recognized by MmeI. After dephosphorylation with alkaline phosphatase CIAP, the purified MmeI-digested products were linked to a second adapter (adapter 2: 5’-GATCGGAAGAGCGGTTCAGCAGGAATGCCGAG-3’). Then, the double adapter-flanked tags from the mRNAs were amplified by PCR using Phusion DNA polymerase using the following program: 98°C for 30 sec, followed by 15 cycles of 98°C for 10 sec, 60°C for 30sec and 72°C for 15 sec, and a final 72°C for 10 min. The resulting 85 bp strips were then purified from a 6% TBE-PAGE gel, denatured and attached to the chip for sequencing.

Single end sequencing was done on an Illumina Hiseq2000 (read length: 50bp) or Illumina Nextseq 500 (read length: 75bp), according to the commercial manuals. Reads with low quality sequences or >4% unknown nucleotides were filtered from the raw data by FASTX Toolkit (-0.0.13.2) (http://hannonlab.cshl.edu/fastx_toolkit). All the resultant clean reads were mapped to the reference database (http://rice.plantbiology.msu.edu; release 7) by SOAP with the maximum number of mismatches allowed = 2 bp and number of valid alignments per read pair (-k) = 40. The numbers of these unambiguous clean tags for each gene was calculated and normalized to TPM (number of transcripts per million clean tags). The fold change (infected versus mock) of expression level for each gene was calculated as the log2 ratio using TPM values. Subsequently, we performed a rigorous significance test to determine the differentially expressed genes [20]. The resulting p-values for all genes were corrected for multiple tests using a FDR (false discovery rate) adjustment [21]. A DGE was defined as a gene with significantly changed expression with a FDR <0.05 and an absolute value of the log2 ratio >1.

### Small-RNA sequencing and statistical analysis

The small RNA libraries were prepared using the standard Solexa protocol. Briefly, 10μg total RNA was size-fractionated in 15% polyacrylamide gel electrophoresis (PAGE) and small RNAs of 18–30 nt were isolated. Then the purified small RNAs were ligated with 5’- and 3’-adaptors at both ends. The ligated small RNAs were further purified and reverse transcribed into cDNAs. These cDNAs were finally amplified using the following PCR program: a reaction at 98°C for 30 sec, followed by 15 cycles of 98°C for 10 sec, 60°C for 30sec and 72°C for 15 sec, and a final 72°C for 10 min. After obtaining the DNA band on 6% PAGE gels, the PCR products were ethanol precipitated and purified using Spin-X filter columns.

Single end sequencing of sRNA libraries was done on an Illumina Hiseq2000 (read length: 50 bp) according to the commercial manual. Low quality reads (single basic Q20 quality <20, ratio>40% in one read) were filtered from the raw data and adaptors were trimmed by FASTX Toolkit (-0.0.13.2) (http://hannonlab.cshl.edu/fastx_toolkit) with parameters -l 18. A custom Perl script was used to filter remaining low quality reads. All clean reads were aligned to miRBase (version 21) by miRDeep-p [22] with the following parameters: mapper.pl = -c -j -l 18 -m -p -s -t–v; quantifier.pl = -p -m -r–W; miRDeep2.pl = collapsed.fa genome.fa genome.arf mature-miRNA.fa mod-miRNA.fa pre-miRNA.fa. The number of perfect clean reads corresponding to each miRNA was calculated and normalized to TPM (TPM = read counts *1, 000, 000/library size). Differential expression between samples was identified if the log_2_ fold change was >1 and the statistical significance calculated by software DEGseq [23] was p<0.05.

The secondary structures of the putative miRNA precursors identified were predicted using RNAfold [24]. Target genes of novel miRNAs were predicted using psRNA Target [25], with the default parameters and a maximum expectation value of 3 (number of mismatches allowed). These genes were analyzed for enriched Gene Ontology (GO) and Kyoto Encyclopedia of Genes and Genomes (KEGG) categories through hypergeometric gene set enrichment analysis [26].

### Validation of the expression of miRNAs and mRNAs by qPCR

Real-time quantification of microRNAs was performed by stem-loop RT-PCR as previously reported [27]. Briefly, about 2 μg purified total RNA were reverse transcribed using miRNA-specific stem-loop primers (50 nM) in a 20 μl reaction volume with the Takara RNA PCR Kit to generate cDNA following the manufacturer’s instructions. The expression patterns of the miRNAs were then analyzed by Real-time Quantitative PCR (qPCR) with a standard SYBR Green I PCR kit (Invitrogen) protocol. Each qPCR reaction contained 10 μl of 2×SYBR PCR master mix, 0.8 μl of forward primer (10 μM), 0.8 μl of reverse primer (10 μM), 0.5μl of cDNA and sterilized ddH2O added up to 20μl. The reactions were incubated in a 96-well plate at 95°C for 10 min, followed by 40 cycles of 95°C for 15 s and 60°C for 1 min. Then, the samples were heated from 60 to 95°C to acquire the denaturing curve of the amplified products. All reactions, including no-template controls, were performed in triplicate on a 7900 Real Time PCR System (Applied Biosystems). Relative expression was calculated using the comparative cycle threshold method [28] and values for each miRNA were normalized to the expression levels of U6, a gene known to be stably expressed in the presence or absence of RSV [14]. All the primers used are listed in the Supporting Information (S1 Table).

The expression of the target gene was also analyzed by qPCR. The total RNA was first treated with DNase I (Takara) and then reverse transcribed to generate cDNA using an oligo(dT) primer and a PrimeScrip RT reagent kit (Takara) according to the manufacturer’s instructions. The target gene primers (S2 Table) were then added to amplify the PCR products. The qPCR was performed in triplicate on a 7900 Real Time PCR System (Applied Biosystems) with a standard SYBR Green I PCR kit (Invitrogen) protocol. In each reaction, the stably-expressed actin gene was used as the internal reference [13]. The reactions were incubated in a 96-well plate at 95°C for 3 min, followed by 40 cycles of 95°C for 15 s, 60°C for 20 s, and 72°C for 30 s. Each experiment was replicated three times. The relative expression levels were calculated using the 2−ΔΔCT method [28]. The values of the threshold cycle (CT) were calculated by Rotor-Gene 6 software (Corbett Robotics, Australia).

## Results

### Overview of Deep sequencing for small RNA libraries

Six small RNA libraries, three each from RSV-infected (RI) and mock-inoculated (CK) plants, were subjected to Solexa high-throughput RNA sequencing. The raw sequence data have been deposited in NCBI database with accession numbers GSM1921841- GSM1921846. After removing all the low-quality reads, RSV-infected samples yielded 4,807,944–5,237,572 clean reads, all between 18 and 28 nucleotides in length, representing 1,377,074–1,503,616 unique reads. Corresponding numbers from the mock-inoculated samples were respectively 4,638,162–4,964,387 and 1,236,819–1,275,653 (Table 1). There was good agreement between replicates. Using SOAP2, 74.1–77.6% reads and 67.1–69.3% uniques from sRNA libraries of RSV-infected rice plants and 87.9–91.8% reads and 80.1–81.3% uniques from libraries of mock-inoculated rice plants mapped well to the rice genome (Table 1).

**Table 1:** Summary of deep sequencing results: small RNAs from virus-infected and mock-inoculated rice small RNA libraries.

Libraries	RSV-infected ^e^			Mock-inoculated ^f^		
	RI- 1	RI-2	RI-3	CK-1	CK-2	CK-3
**Uniques** ^a^	1503616	1377074	1437284	1236819	1259375	1275653
**Reads** ^b^	5237572	4807944	4850785	4822413	4638162	4964387
**Uniques mapped to the rice genome** ^c^	1041466 (69.3%)	948160 (68.9%)	964862 (67.1%)	991835 (80.1%)	1015936 (80.7%)	1037105 (81.3%)
**Reads mapped to the rice genome**	3897231 (74.4%)	3562517 (74.1%)	3763478 (77.6%)	4331875 (89.8%)	4256023 (91.8%)	4367542 (87.9%)
**Uniques mapped to the virus genome** ^d^	87018 (5.7%)	79421 (5.8%)	74246 (5.1%)	34	29	23
**Reads mapped to the virus genome**	434958 (8.3%)	386592 (8.0%)	398842 (8.2%)	36	30	43

^a^ total number of uniques from each of the three replicates within the set (18–28 nt in length).

^b^ Total number of small RNA reads from each of the three replicates within the set (18–28 nt in length).

^C^ Sequences with perfect match to the rice genome, including those from tRNA, rRNA, snRNA, or snoRNAs.

^d^ Sequence with no more than 1 nt mismatch to the RSV genome.

^e^ RSV-infected: Small RNA library from RSV-infected rice.

^f^ Mock-inoculated: Small RNA library from mock-inoculated rice.

In the small RNA libraries from RSV-infected rice plants, similar percentages of high-quality reads and uniques were well matched with the RSV genome (8.0%-8.3% reads and 5.1–5.8% uniques), whereas negligible numbers (less than 50 reads) were obtained from the healthy controls (Table 1). The data show the high quality of the small RNA libraries, indicating that they are suitable for further comparative analysis.

### RSV infection selectively altered expression of rice miRNAs

**Fig 1:**
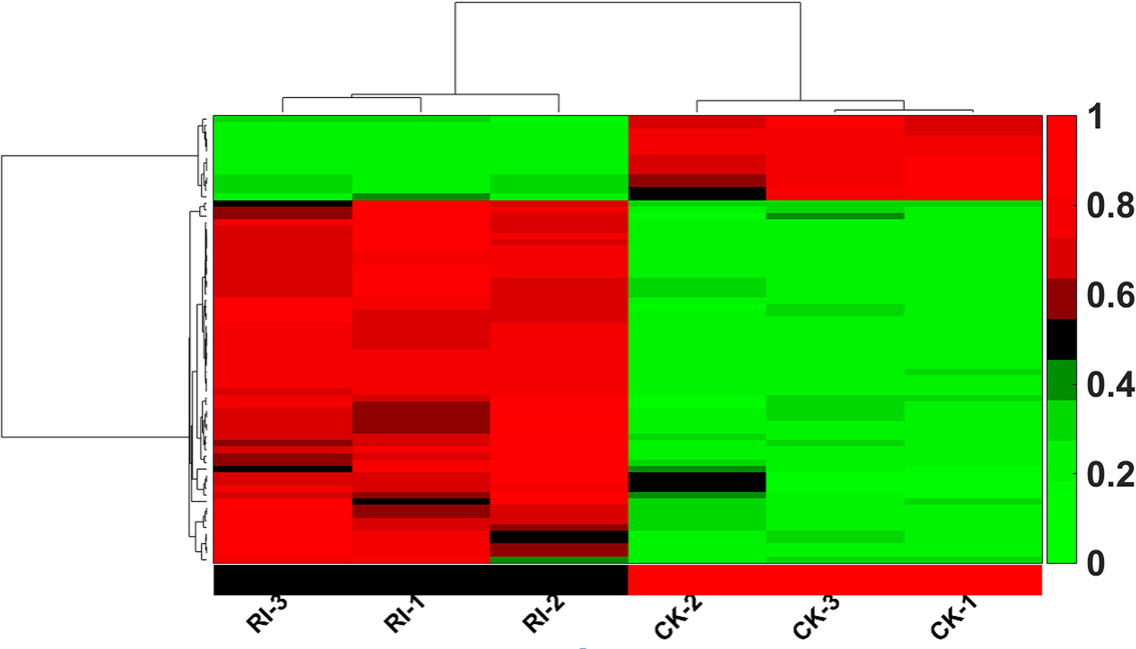
Differentially expressed miRNAs in RSV-infected (RI-1~RI-3) and mock-inoculated (CK-1~CK-2) rice plants. Every row shows a different miRNA. Green, black and red indicate expression levels of miRNAs, respectively low, medium and high.

The most recent version of miRBase, V21, catalogues more than 30,000 mature miRNAs from 223 species of organism (http://www.mirbase.org/) [29] of which 713 mature miRNAs, derived from 592 precursors, have been identified in rice. A total of 398066–425473 reads (8765–8912 uniques) from RSV-infected rice plants and 365783–392527 reads (5782–6035 uniques) from mock-inoculated rice plants matched perfectly to the rice miRNA precursors (Table 2). When aligned with the mature rice miRNAs, 1017–1566 uniques, representing 570 species of known mature miRNA, were obtained from these six small RNA libraries. Thus our libraries included the majority of all known rice miRNAs and can be used effectively to explore the rice miRNAs regulated by RSV infection. After normalization of the raw sequence read numbers, the average normalized reads of three independent biological replicates of mock-inoculated and RSV-infected libraries were then selected for further analysis of differential expression levels of miRNAs. Using stringent criteria (the average value of three independent biological replicates conformed to the parameters: p<0.05, fold change >2 and more than 100 reads in at least one library after normalization), we identified 69 miRNAs that were differentially expressed. Of those, 56 miRNAs were highly up-regulated in expression, while 13 were extremely down-regulated (Fig 1 and S3 Table).

**Table 2:** Summary of small RNAs mapped to known rice miRNA precursors in virus-infected and mock-inoculated rice plants.

	RSV-infected						Mock-inoculated					
	RI-1		RI-2		RI-3		CK-1		CK-2		CK-3	
	Unique sequences	Reads/million	Unique sequences	Reads/ million	Unique sequences	Reads/million	Unique sequences	Reads/ million	Unique sequences	Reads/ million	Unique sequences	Reads/ million
**Precursor**^a^	8,819	425,473	8,765	398,066	8,912	408,329	5,782	392,527	6,035	365,783	5,836	375,684
**miRNA**^b^	1,566 (17.8%)	415,262 (97.6%)	1,239 (14.1%)	386,866 (97.2%)	1,467 (16.4%)	394,423 (96.6%)	1,087 (18.8%)	363,087 (92.5%)	1,017 (16.8%)	326,179 (89.1%)	1,049 (18.0%)	346,381 (92.2%)
**Others**	7,253 (82.2%)	10,211 (2.4%)	7,526 (85.9%)	11,200 (2.8%)	7,445 (83.6%)	13,906 (3.4%)	4,695 (81.2%)	29,440 (7.5%)	5,018 (83.2%)	39,604 (10.9%)	4,787 (82.0%)	29,303 (7.8%)

^a^ Perfect match to sense miRNA precursor sequences from the miRBase database (http://microrna.sanger.ac.uk/sequences, version 21)

^b^ Encompasses the defined miRNA sequence ±1 nt on each side.

**Fig 2:**
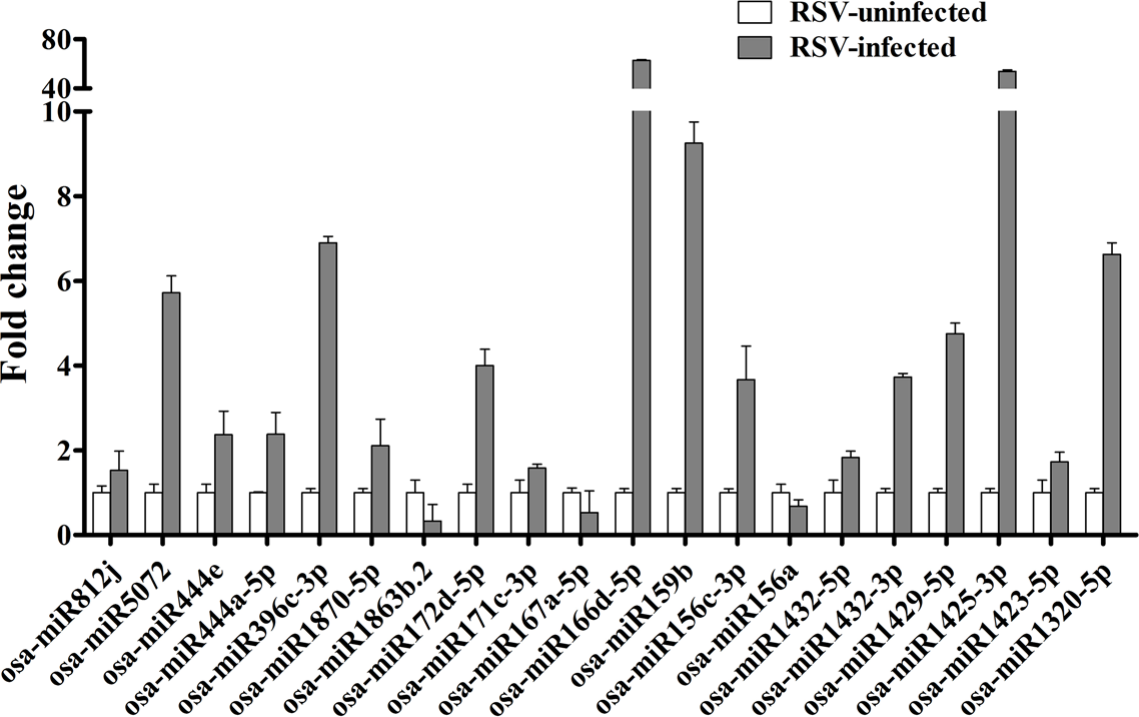
The relative levels of 20 differentially expressed miRNAs as shown by stem-loop qRT-PCR.

To validate the differences in expression levels suggested by the deep sequencing, 20 miRNAs (17 shown to be up-regulated and 3 down-regulated miRNAs in deep sequencing; S4 Table), were selected for qRT-PCR assays using the primers listed in S1 Table. osa-miR156a, -167a-5p and -1683b.2 were significantly down-regulated with fold changes ranging from 0.33 to 0.68 while the other 17 tested miRNAs were significantly up-regulated with fold changes ranging from 1.53 to 62.71 (Fig 2 and S4 Table). These results are very similar to those suggested by the deep sequencing analysis.

**Fig 3:**
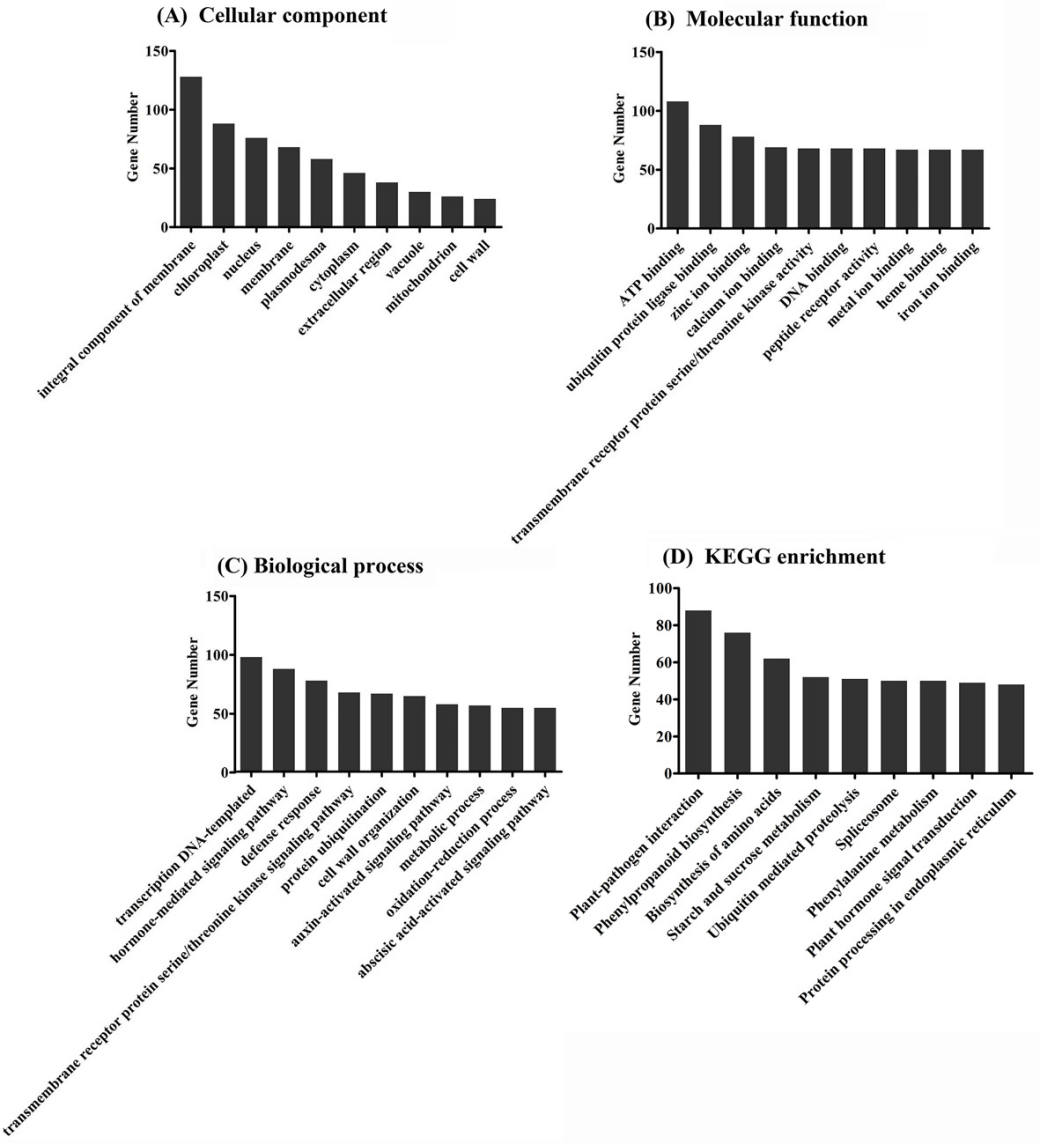
Gene ontology (GO) (A-C) and pathway enrichment (D) analysis of predicted targets by differentially expressed miRNAs.

GO categorization of the predicted targets of these differentially expressed miRNAs showed that they are involved in a broad range of processes, and that at least 100 targets were related to integral components of membrane, chloroplasts, nucleus, ATP binding, ubiquitin protein ligase binding, zinc ion binding, DNA-templated transcription, hormone-mediated signaling pathway, and defense responses (Fig 3A–3C). This is consistent with important regulatory roles for these differentially expressed miRNAs during rice growth, development, and responses to biotic and abiotic stress.

When these targets were mapped into the KEGG pathway database by applying a cut-off criterion of P<0.05, it was obviously that the targets involved in the plant-pathogen interaction pathway were more enriched than those in other pathways (Fig 3D).

### Overview of Deep sequencing for mRNA libraries

In order to profile the global gene expression and survey the profiling of all the miRNA targets that are differentially expressed in response to RSV stress, mRNA libraries were constructed for three replicates each of RSV-infected and mock-inoculated plants and sequenced as previously described. The raw sequence data have been deposited in NCBI database with accession numbers GSM1921835- GSM1921840. In total 14,492,224–14,835,261 and 14,549,952–14,881,310 reads were sequenced respectively from mRNA libraries of RSV-infected and mock-inoculated rice plants. The raw sequence data were filtered to remove low quality tags/reads and the resultant clean tags/reads were further mapped to rice genes. In the samples from RSV-infected rice plants, 63.86–65.10% of clean reads and 91.18–94.40% of uniques could be perfectly mapped to 12,795–12,863 rice genes (Table 3). In the three replicates from mock-inoculated rice plants, 59.69–62.26% of clean reads and 90.41–92.46% of uniques could be perfectly mapped to 12,153–12,531 rice genes (Table 3). The similar percentages of clean reads, mapped reads and uniques and the similar numbers of mapped genes indicates that these mRNA libraries are suitable for further comparative analyses of gene expression profiles.

**Table 3:** Summary of mRNA expression libraries.

libraries	RSV-infected			Mock-inoculated		
	RI-1	RI-2	RI-3	CK-1	CK-2	CK-3
Total Reads	14,835,261	14,492,224	14,732,837	14,881,310	14,669,472	14,549,952
Clean Reads	12,320,457 (83.05%)	12,017,087 (82.92%)	12,184,656 (82.70%)	12,536,406 (84.24%)	12,564,718 (85.65%)	12,904,497 (88.69%)
Gene Mapped Reads	7,909,384 (64.20%)	7,673,824 (63.86%)	7,931,898 (65.10%)	7,804,736 (62.26%)	7,679,041 (61.12%)	7,702,403 (59.69%)
Gene Mapped Unique Reads	7,211,957 (91.18%)	7,274,850 (94.80%)	7,435,947 (93.75%)	7,055,951 (90.41%)	7,078,078 (92.17%)	7,121,819 (92.46%)
Reads Perfect Match	3,816,259 (48.25%)	3,612,234 (47.07%)	3,946,378 (49.75%)	3,258,439 (41.74%)	3,916,967 (51.00%)	3,627,663 (47.10%)
No. of Mapped genes	12,863	12,795	12,797	12,531	12,438	12,153

Note

Total Reads: the raw data after sequencing

Clean Reads: the reads after filtering out low-quality tags, unexpected-length tags, and single-copy tags

Gene Mapped Reads: the reads of the clean reads that could be mapped to the rice genome

Reads perfect match: the reads of the gene that could be mapped to the rice genome with 0 mismatch.

### Identification of rice genes affected by RSV infection

**Fig 4:**
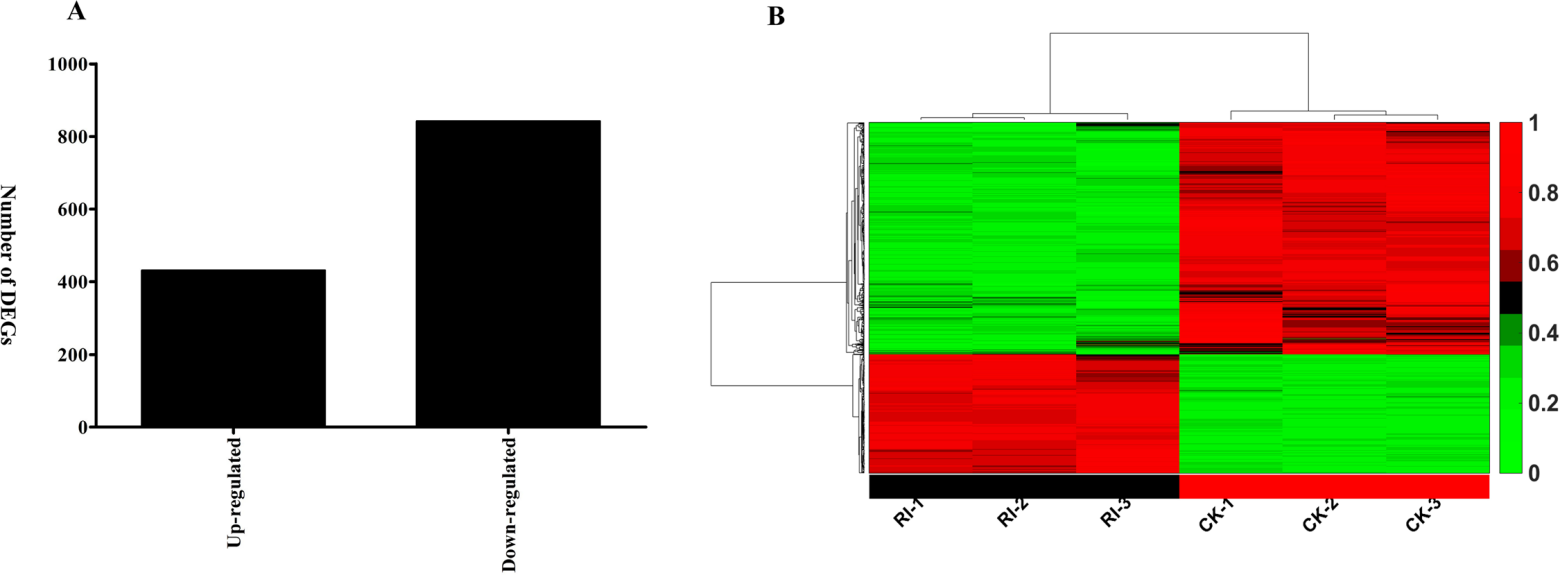
Differentially expressed mRNAs in RSV-infected and mock-inoculated rice plants. (A) Total numbers of up- and down-regulated DEGs; (B) Differentially expressed mRNAs in RSV-infected (RI-1~RI-3) and mock-inoculated (CK-1~CK-2) rice plants. Every row shows a different gene. Green, black and red indicate expression levels of genes, respectively low, medium and high.

To compare the mRNA expression profiling between the RSV-infected and mock-inoculated rice plants, the sequence data were normalized and analyzed using DEGseq software with the criteria of fold changes >2 and P<0.05 to identify significantly differentially expressed genes. The genome-wide expression profiling of host response to RSV infection was carried out using Digital Gene Expression Tag Profiling (DGE) [23, 30–31]. The averages of the three independent biological replicates of RSV-infected and non-infected rice plants were used for further analyses. Of the 1,274 genes differentially expressed, 431 were up-regulated and 843 were down-regulated in response to RSV infection (Fig 4A, S5 Table). The fold-change of gene expression ranged from 0 to 1036.15. Hierarchical cluster analysis validated the reproducibility of the results among the replicates (Fig 4B).

**Fig 5:**
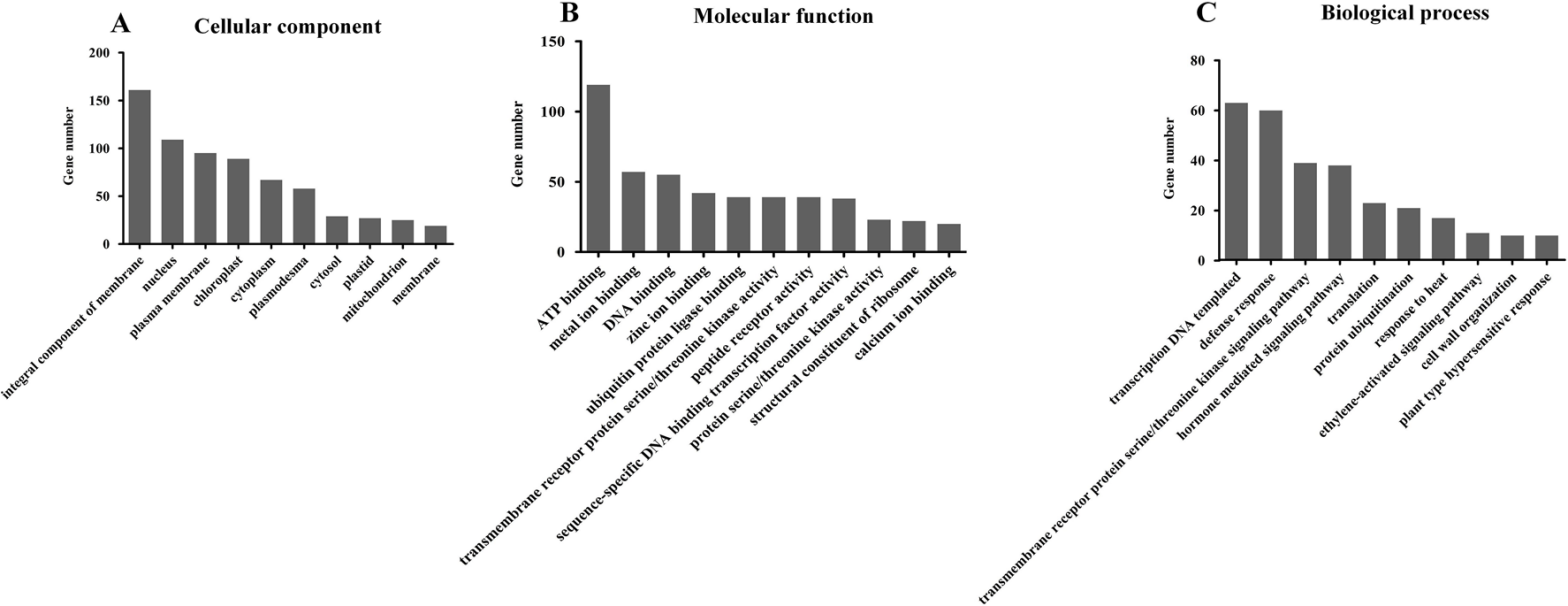
Gene ontology (GO) and pathway enrichment analysis of differentially expressed mRNAs. (A) Category of cellular components; (B) Category of molecular functions; (C) Category of biological process.

Gene ontology (GO) and pathway enrichment analyses were next performed (Fig 5). The main categories identified by GO were the integral components of membranes, nucleus, ATP binding, DNA-templated regulation of transcription, and defense response (Fig 5), similar to the predicted targets of the differentially expressed miRNAs (Fig 3).

To obtain more biological information about the molecular and biological responses induced by RSV infection, the RSV-responsive genes were matched to processes in the KEGG pathway. By applying a cut-off criterion of P<0.05, the results of enrichment analysis revealed a few important pathways that were significantly enriched after RSV infection (Table 4). It was obvious that genes involved in the plant-pathogen interaction pathway were most significantly enriched among the down-regulated genes, while genes involved in metabolic pathways, ribosomes and photosynthesis were significantly enriched among the up-regulated genes. These results indicate that a complex gene expression network is affected by RSV infection.

**Table 4:** Significant pathways and proportions after KEGG (Kyoto Encyclopedia of Genes and Genomes) analysis of differentially expressed genes after RSV infection (P<0.05).

	Pathway ID	DEGs with pathway annotation	P-value	Pathway
**Down regulated**	KO04626	25	5.00E-03	Plant-pathogen interaction
	KO00904	7	1.37E-03	Diterpenoid biosynthesis
	KO05204	6	3.75E-03	Chemical carcinogenesis
	KO00980	6	4.48E-03	Metabolism of xenobiotics by cytochrome P450
	KO00941	5	3.28E-03	Flavonoid biosynthesis
	KO03060	5	3.69E-03	Protein export
	KO04621	3	3.68E-03	NOD-like receptor signaling pathway
	KO05223	3	4.21E-03	Non-small cell lung cancer
**Up regulated**	KO00195	37	4.98E-24	Photosynthesis
	KO03010	37	1.36E-11	Ribosome
	KO00190	20	1.91E-07	Oxidative phosphorylation
	KO00710	7	2.99E-03	Carbon fixation in photosynthetic organisms
	KO01100	84	1.29E-03	Metabolic pathways
	KO00402	3	3.07E-02	Benzoxazinoid biosynthesis
	KO00630	5	2.83E-02	Glyoxylate and dicarboxylate metabolism
	KO00500	11	4.00E-02	Starch and sucrose metabolism

### Integrated analysis of miRNA and mRNA expression profiles

**Fig 6:**
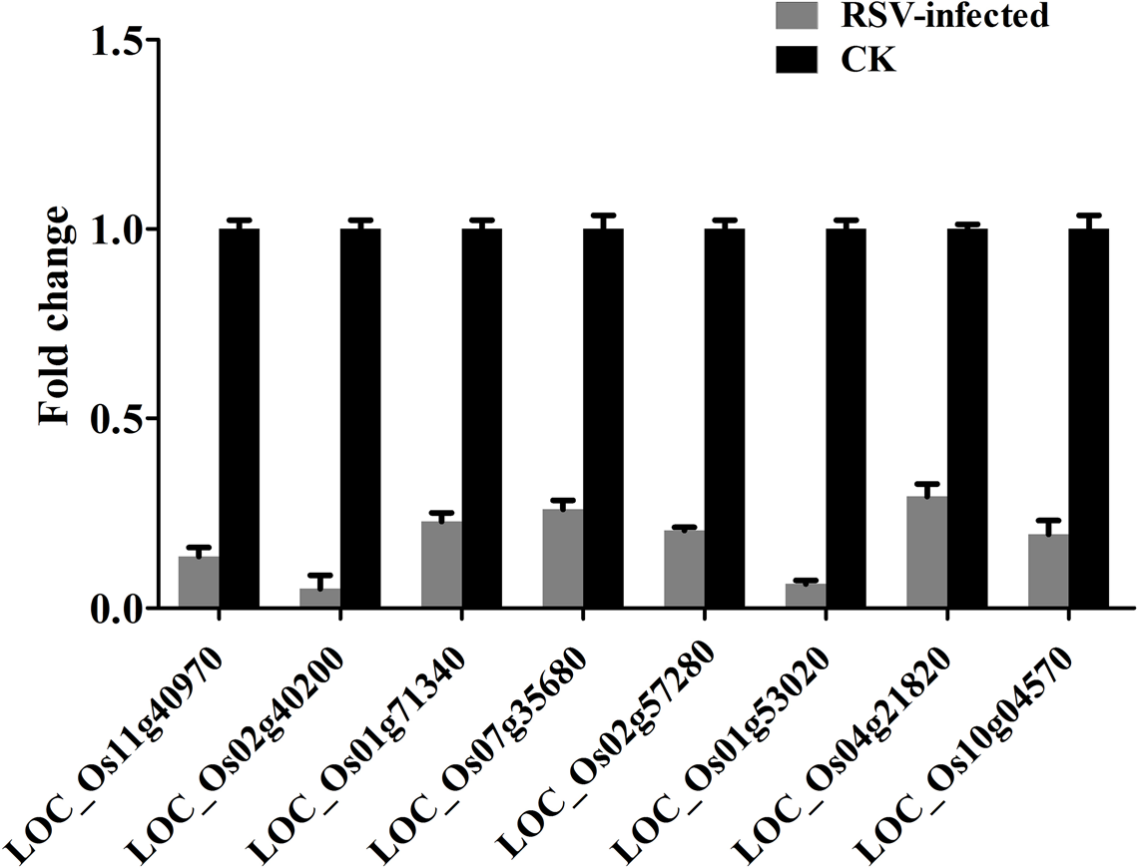
The relative levels of 8 differentially expressed disease resistance genes evaluated by qRT-PCR. These genes were identified as LRR receptor-like serine/threonine-protein kinase (LOC_Os11g40970) (target of Osa-miR159b), LRR receptor-like serine/threonine-protein kinase (LOC_Os02g40200) (target of Osa-miR444e), Glucan endo-1,3-beta-glucosidase (LOC_Os01g71340) (target of Osa-miR1320-5p), Cysteine-rich receptor-like protein kinase 8 (LOC_Os07g35680) (target of Osa-miR1429-5p and Osa-miR1432-3p), Brown planthopper-induced resistance protein 6 (LOC_Os02g57280) (target of Osa-miR1432-5p), heat shock protein DnaJ (LOC_Os01g53020) (target of Osa-miR172d-3p), Wall-associated receptor kinase 5 (LOC_Os04g21820) (target of Osa-miR396c-3p), and Putative disease resistance protein RGA4 (LOC_Os10g04570) (target of Osa-miR5072).

**Fig 7:**
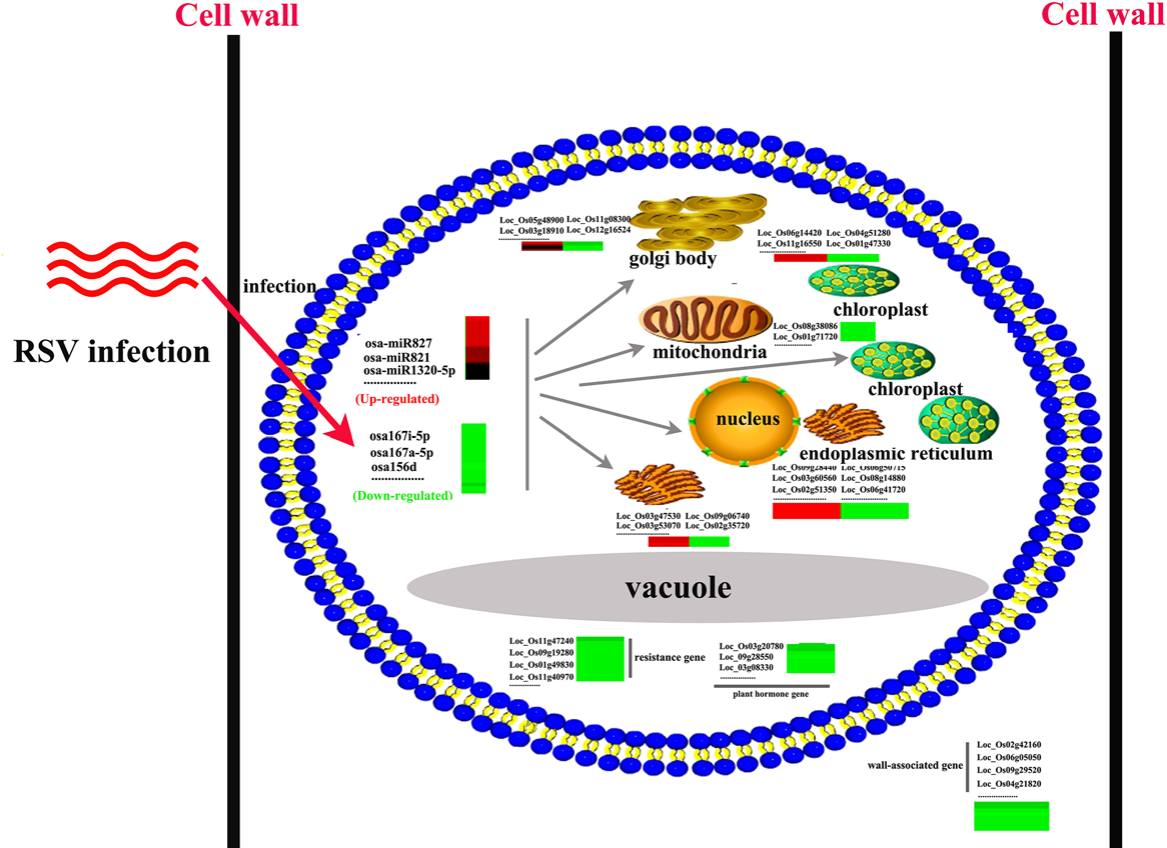
The global cellular responses of susceptible rice plants to RSV infection. Green and red rows/columns indicate that expression levels of miRNAs/ genes were down- or up-regulated, respectively.

In most cases, miRNAs act as negative regulators for their target mRNAs and therefore high levels of miRNAs can significantly down-regulate their target mRNAs [32]. Correlation analysis was performed using the 69 differentially expressed miRNAs (56 up-regulated and 13 down-regulated) and the 1274 differentially expressed mRNAs (431 up-regulated and 843 down-regulated) between RSV infected and mock-infected rice plants. Interestingly, it was found that 63 differentially expressed miRNAs were significantly and negatively correlated with 160 target mRNAs; 52 up-regulated miRNAs were negatively correlated with 150 down-regulated target mRNAs and 11 down-regulated miRNAs were negatively correlated with 10 up-regulated target mRNAs (S6 Table). Moreover, 22 of these up-regulated miRNAs corresponding to 24 down-regulated target genes have been reported to be involved in disease resistance (Table 5), whereas none of the up-regulated target genes was reported to be a disease resistance gene (S6 and S7 Tables). In addition, 22 down-regulated mRNAs targeted by 23 up-regulated miRNAs were related to chloroplasts whereas only one up-regulated mRNA targeted by 4 down-regulated members of miR167-5p (Table 6) is known to be chloroplast-related. This suggests that the functions of chloroplasts are affected by RSV infection. In order to confirm the relationship of these target genes with the corresponding miRNAs, 8 target genes related to disease resistance were selected for further qPCR assay. The target mRNAs were down-regulated with fold changes ranging from 0.04 to 0.4, consistent with the results of DEG (Fig 6) and indicating a negative relationship between these disease resistance genes and up-regulated miRNAs. Thus a complex pattern of miRNAs and mRNAs was regulated by viral infection. The global cellular responses of susceptible rice plants to RSV infection are summarized in Fig 7 according to the putative functions of the differentially expressed miRNAs and mRNAs.

**Table 5:** The target genes related to plant disease resistance.

miRNA	Targets	
	Gene ID	Description
osa-miR1320-3p	LOC_Os11g47240	Probable LRR receptor-like serine/threonine-protein kinase
osa-miR1320-5p	LOC_Os01g71340	Glucan endo-1,3-beta-glucosidase
osa-miR1423-5p	LOC_Os06g34400	RING-H2 finger protein ATL57
	LOC_Os09g19280	Disease resistance protein RPM1
osa-miR1425-3p	LOC_Os01g49830	AP2/ERF domain-containing protein
osa-miR1429-5p	LOC_Os07g35680	Cysteine-rich receptor-like protein kinase 8
osa-miR1432-3p	LOC_Os02g42160	Wall-associated receptor kinase-like 1
	LOC_Os07g35680	Cysteine-rich receptor-like protein kinase 8
osa-miR1432-5p	LOC_Os02g57280	Brown planthopper-induced resistance protein 6
osa-miR156j-3p	LOC_Os02g40180	Probable LRR receptor-like serine/threonine-protein kinase
osa-miR159a.1	LOC_Os10g04730	Cysteine-rich receptor-like protein kinase 5
	LOC_Os11g40970	Probable LRR receptor-like serine/threonine-protein kinase
osa-miR159a.2	LOC_Os01g02700	Probable receptor-like protein kinase
	LOC_Os01g02400	Probable receptor-like protein kinase
	LOC_Os02g17710	Probable leucine-rich repeat receptor-like protein kinase
	LOC_Os06g05050	Wall-associated receptor kinase 3
	LOC_Os11g40970	Probable LRR receptor-like serine/threonine-protein kinase
osa-miR159b	LOC_Os10g04730	Cysteine-rich receptor-like protein kinase 5
	LOC_Os11g40970	Probable LRR receptor-like serine/threonine-protein kinase
osa-miR159d	LOC_Os09g29520	Wall-associated receptor kinase 3
	LOC_Os10g04730	Cysteine-rich receptor-like protein kinase 5
	LOC_Os11g40970	Probable LRR receptor-like serine/threonine-protein kinase
osa-miR159e	LOC_Os09g29520	Wall-associated receptor kinase 3
	LOC_Os10g04730	Cysteine-rich receptor-like protein kinase 5
	LOC_Os11g40970	Probable LRR receptor-like serine/threonine-protein kinase
osa-miR164a	LOC_Os06g38340	Probable LRR receptor-like serine/threonine-protein kinase
	LOC_Os12g43410	Thaumatin-like protein
	LOC_Os02g57280	Brown planthopper-induced resistance protein 5
	LOC_Os11g40970	Probable LRR receptor-like serine/threonine-protein kinase
osa-miR164f	LOC_Os06g38340	Probable LRR receptor-like serine/threonine-protein kinase
	LOC_Os12g43410	Thaumatin-like protein
	LOC_Os02g57280	Brown planthopper-induced resistance protein 4
	LOC_Os11g40970	Probable LRR receptor-like serine/threonine-protein kinase
	LOC_Os12g43410	Thaumatin-like protein
	LOC_Os02g57280	Brown planthopper-induced resistance protein 3
osa-miR172c	LOC_Os12g16540	Wall-associated receptor kinase 3
	LOC_Os01g53020	heat shock protein DnaJ
osa-miR172d-3p	LOC_Os12g16540	Wall-associated receptor kinase 3
	LOC_Os01g53020	heat shock protein DnaJ
	LOC_Os02g57280	Brown planthopper-induced resistance protein 2
osa-miR396c-3p	LOC_Os04g21820	Wall-associated receptor kinase 5
osa-miR444a-5p	LOC_Os02g57280	Brown planthopper-induced resistance protein 1
osa-miR444c.2	LOC_Os01g02400	Probable receptor-like protein kinase
osa-miR444e	LOC_Os02g40200	Probable LRR receptor-like serine/threonine-protein kinase
osa-miR5072	LOC_Os10g04570	Putative disease resistance protein RGA4
osa-miR1870-5p	LOC-Os02g40190	LRR receptor-like serine/threonine-protein kinase EFR

**Table 6:** Integrative analysis of differentially expressed miRNAs that are negatively correlated with chloroplast-related genes.

microRNA	regulation	Gene ID	regulation	Description
osa-miR1423-5p	up	LOC_Os02g37090	down	Protein PHYLLO, chloroplastic
osa-miR1432-5p	up	LOC_Os12g16200	down	Glutathione synthetase, chloroplastic
		LOC_Os06g02500	down	Superoxide dismutase [Fe], chloroplastic
osa-miR164a, b, f	up	LOC_Os04g37619	down	Zeaxanthin epoxidase, chloroplastic
		LOC_Os07g48510	down	Thioredoxin-like 1–1, chloroplastic
osa-miR166b, d-5p	up	LOC_Os08g37700	down	33 kDa ribonucleoprotein, chloroplastic
osa-miR167a, c, e, i-5p	down	LOC_Os04g31040	up	Violaxanthin de-epoxidase, chloroplastic
osa-miR167e, i-3p	up	LOC_Os03g52460	down	Glucose-1-phosphate adenylyltransferase large subunit 1, chloroplastic
osa-miR167h-3p	up	LOC_Os12g08830	down	PsbP domain-containing protein 4, chloroplastic
osa-miR172c	up	LOC_Os01g44980	down	Peptide deformylase 1B, chloroplastic
osa-miR172d-3p	up	LOC_Os01g44980	down	Peptide deformylase 1B, chloroplastic
		LOC_Os07g08340	down	Pyruvate kinase isozyme A, chloroplastic
osa-miR1861h, j	up	LOC_Os08g37700	down	33 kDa ribonucleoprotein, chloroplastic
osa-miR1883a	up	LOC_Os10g40030	down	Short-chain dehydrogenase TIC 32, chloroplastic
osa-miR396c-3p	up	LOC_Os03g11670	down	Pentatricopeptide repeat-containing protein, chloroplastic
		LOC_Os07g32590	down	Methionine aminopeptidase 1B, chloroplastic
osa-miR444a-5p	up	LOC_Os08g41990	down	Glutamate-1-semialdehyde 2,1-aminomutase, chloroplastic
osa-miR5072	up	LOC_Os02g33020	down	Heme-binding-like protein, chloroplastic
		LOC_Os12g16200	down	Glutathione synthetase, chloroplastic
osa-miR812a, b, c, d, e	up	LOC_Os07g48510	down	Thioredoxin-like 1–1, chloroplastic

### RSV infection significantly affected the plant-pathogen interaction pathway

**Fig 8:**
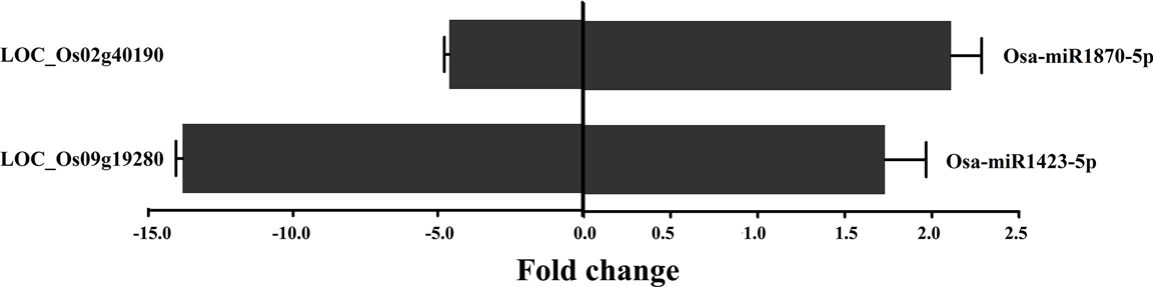
The fold changes of two pairs of miRNAs and their targets, osa-miR1423-5p/ RPM1 (LOC-Os09g19280) and -1870-5p/ EFR (LOC_Os02g40190), indicating a negative correlation.

**Fig 9:**
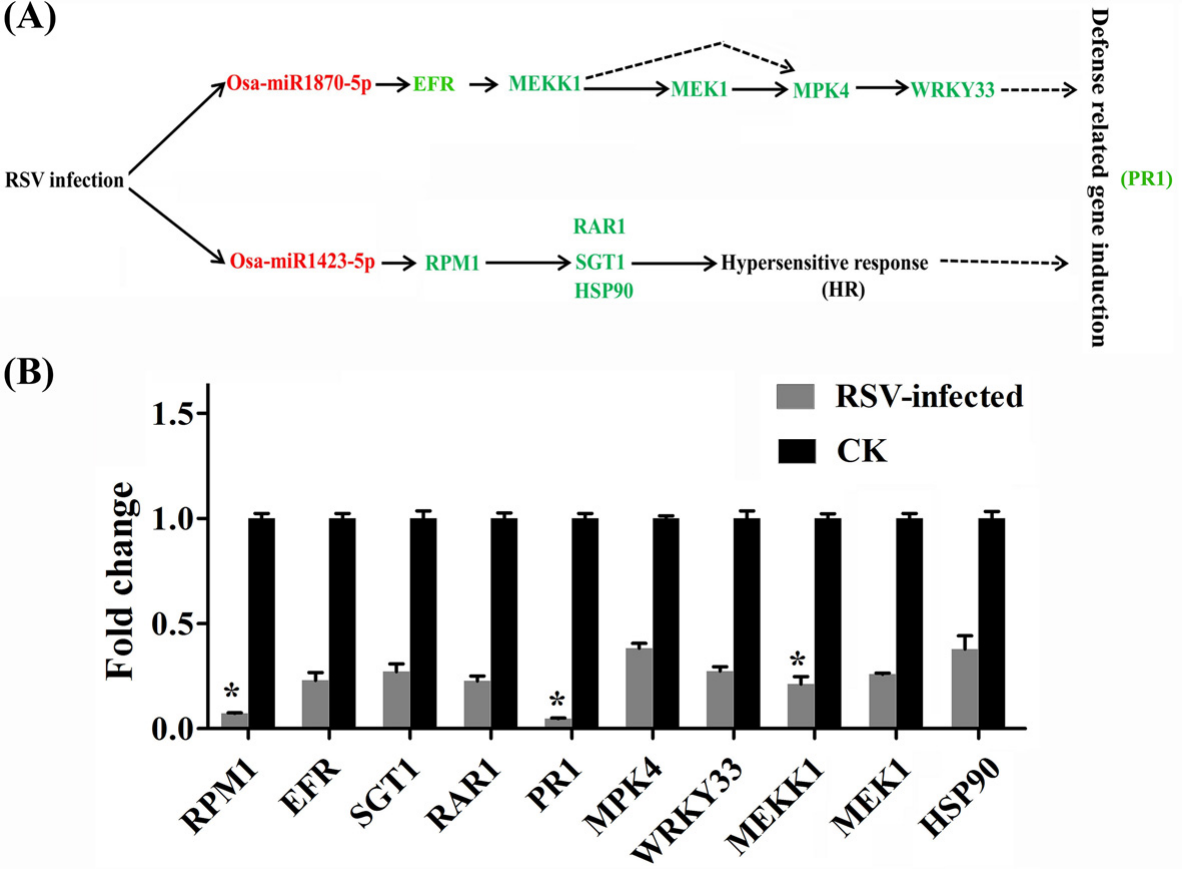
The plant-pathogen interaction pathways altered by RSV infection. (A) The plant-pathogen interaction pathways; (B) The relative levels of genes involved in the plant-pathogen interaction pathway evaluated by qRT-PCR. Green and red indicate that the expression of miRNAs/ genes were down- or up-regulated, respectively. These genes included RPM1 (LOC_Os09g19280), EFR (LOC_Os02g40190), SGT1 (LOC_Os08g34740), RAR1 (LOC_Os02g33180), PR1 (LOC_Os03g18850), MPK4 (LOC_Os06g48590), WRKY33 (LOC_Os03g55164), MEKK1 (LOC_Os03g49640), MEK1 (LOC_Os06g05520), and HSP90 (LOC_Os09g29840).

As shown above, the changes in miRNA and mRNA expression most frequently and significantly identified were those associated with the plant-pathogen interaction pathways (Table 5). In particular, two pairs of miRNAs and their targets, osa-miR1423-5p/ disease resistance protein RPM1 (LOC-Os09g19280) and -1870-5p/ LRR receptor-like serine/threonine-protein kinase EFR (LOC_Os02g40190), were found to be negatively correlated in response to RSV infection and both have previously been associated with plant-pathogen interactions [33, 34]. We therefore used qRT-PCR to explore further the relationship between these two target genes and their corresponding miRNAs. In response to RSV, osa-miR1423-5p and -1870-5p were up-regulated with fold changes from 2.8 to 4.2 and their corresponding target RPM1 and EFR mRNAs were down-regulated with fold changes from 3.5 to 11.3 (Fig 8), confirming the trends suggested by the deep sequencing data. Similar qRT-PCR experiments to investigate the responses of related disease resistance genes down-stream in the plant-pathogen pathway (Fig 9A) showed that these were also down-regulated by RSV infection. Changes to important genes in the miR1870-5P-mediated EFR resistance pathway (WRKY 33, MEK1, MPK4, and MEKK1) were 2.6–4.7 fold and those in the RPM1 pathway (RAR1, SGT1 and HSP90) were 2.6–4.3 (Fig 9B). The results demonstrate that RSV infection alters the osa-miR1423-5p and osa-miR1870-5p-mediated pathways by up-regulating the miRNAs and suppressing the defense response in susceptible plants.

## Discussion

### RSV infection selectively altered the miRNA expression pattern in host plants

Typically, miRNAs are at the core of gene regulatory networks, because their target genes are often gene expression regulators (e.g. including transcription factors and F-box proteins) or pivotal proteins involved in various pathways [32]. Recent studies have shown that expression of several miRNA families is regulated by abiotic and biotic stress, suggesting an important role of these miRNAs in stress response [35–37]. In this study, approximately 600 mature miRNAs were detected, the most abundant of which were miR168, 167, 156, 397, 172, 166, 164, 528, and 159 in both infected and healthy plants. This supports the view that they play essential roles in rice growth and development, and is mostly consistent with previous reports [14, 38].

In most cases, the expression levels of the miRNA-5p and 3p processed from the same precursor did not appear to be correlated or to be significantly regulated by viral infection. However, both the 5p and 3p of miR-1320, -1432, and -172d were significantly up-regulated by RSV infection whereas osa-miR156c-3p, -156g-3p, -156j-3p, osa-miR167a-3p, -167e-3p, -167h-3p, -167i-3p, which have been thought to be miRNA*s and to be subject to degradation during miRNA biogenesis [39], accumulated to significantly higher levels in RSV-infected plants than their down-regulated counterparts. These differences suggested that RSV infection might not only regulate the transcription but also affect the processing and maturation of these specific miRNAs in different ways. Both 5p and 3p of miR-1320, -1432, -172d, -156c, -156g, -156j, -167a, -167e, -167h, and -167i were negatively correlated with their targets by RSV infection (S6 Table), suggesting that all of them may play a role in response to infection.

A previous study showed that viral infection induces the accumulation of novel or phased siRNAs or miRNAs from several conserved cellular miRNA precursors [14]. In our study, the numbers of unique miRNA precursors were much greater in RSV-infected rice plants than in mock plants. About 85% of these extra uniques were produced from the flanking sequences of miRNA isoforms within precursors, indicating that RSV infection induces some new cleavage sites within miRNA precursors.

The miRNA expression pattern in RSV-infected plants was obviously distinct from that reported for other virus-infected plants. In TMV-infected *Arabidopsis thaliana* plants, all microRNAs except for miR160 accumulated to a higher level, and miR163, 164 and 167 showed remarkable increases of up to 7–26 times [40]. In tobacco plants infected by TMV, ToMV, PVX, TEV, and PVY, miRNAs 156, 160, 164, 166, 169, and 171 were most severely affected. These miRNA changes were greatest in response to TMV and ToMV, moderate to TEV and PVX, and least to PVY, and correlated with the severity of disease symptoms [41]. By contrast, in the RSV-infected plants reported here, 13 differentially-expressed miRNAs were down-regulated but 56 were up-regulated (S3 Table). Most miRNA families had only a few or no members that were affected by RSV infection, whereas in some families (e.g. -156, -1320, 393, and -444) all members were regulated by RSV infection. These different miRNA expression patterns can be related to the type of symptoms caused by different virus infections, suggesting that viruses can selectively perturb miRNA pathways and thus cause specific disease symptoms. Recent studies showed that different strains or viruses have the potential to change the miRNA profiles independently of their silencing suppression activity [41, 42] although both silencing suppression activity and miRNA profile alteration of CMV 2b were thought to be related to differences in the stability of complexes formed between 2b and AGO1 proteins [43]. Of the 13 down-regulated miRNAs in RSV infected plants, most (8 and 4 respectively) belong to the miR156 and 167 families, which are conserved across the plant kingdom and have numerous members [32, 40]. In previous studies, alteration of only one Osa-miR156 gene resulted in abnormal phenotypes [44–46]. In this study all members of the miR156 family were consistently down-regulated 9–15 times after RSV infection, suggesting that this activity may be partially responsible for disease symptoms. However, the precise mechanism by which RSV selectively altered the expression of these specific miRNAs remains to be determined. It would be interesting to determine whether the viral silencing suppressor p3 (NS3) encoded on the sense strand RNA 3 [47] and P2, which binds to OsSGS3 [48], are involved in the selective modification of miRNA expression patterns.

### RSV may block the cellular defense response in rice plants

Plants are vulnerable to attack from many viruses and share several sophisticated defense pathways, including the salicylic acid- /ethylene /jasmonic acid-mediated defense responses and hypersensitive reactions (HR), that inhibit viral replication and movement [49]. The defense response systems are known to be triggered in many compatible virus-host interactions, including responses to RYMV, RTSV, RDV, CMV, PVX, and TMV [50–52]. Here high throughput sequence data showed that dozens of miRNAs (S4 Table) and more than one thousand genes (S6 Table), related to a variety of molecular functions, were significantly regulated by RSV at the expression level, reflecting a complex interaction between RSV and its host (Fig 7). In the susceptible cultivar, infection by RSV selectively modified the expression of host genes, which is consistent with previous results [13]. Unexpectedly, most of the differential changes were to up-regulate miRNAs and down-regulate mRNAs. Of the up-regulated miRNAs, the normalized reads of 7, namely Osa-miR166d-5p, 167h-3p, 1320-5p, 1425-3p, 1432-5p, 1870-5p, and 2871a-5p (S3 and S6 Tables), were more than one thousand in infected plants and >10-fold more abundant than in healthy plants. Most of these miRNAs and their targets are related to the stresses. For example, Osa-miR166d-5p and LOC_Os01g07590 (universal stress protein domain containing protein), Osa-miR167h-3p and LOC_Os06g22440 (Jasmonate O-methyltransferase), Osa-miR1425-3p and LOC_Os01g49830 (AP2/ERF domain-containing protein), Osa-miR1432-5p and LOC_Os02g57280 (Brown planthopper-induced resistance protein 6), Osa-miR1870-5p and LOC_Os02g40190 (LRR receptor-like serine/threonine-protein kinase EFR) have all been suggested to be regulated under abiotic or biotic stresses. Almost all differentially expressed cell wall-associated genes were decreased, which is similar to results with RDV [52], but most differentially expressed genes related to defense response appeared to be reduced in the case of RSV but activated in RDV [12, 13]. Some members of the APETALA 2 (AP2), dehydration-responsive element-binding protein (DREBP), and ethylene-responsive element-binding protein (EREBP) gene families, vital transcription factors involved in signaling, stress responses and plant defenses, were significantly and selectively up- or down regulated by RSV infection (S8 Table), suggesting that these changes play a key role in response to viral infection [53, 54]. Furthermore, all tested down-stream genes of the miR1870-5p- and miR1423-5p-mediated disease resistance pathways were significantly down-regulated in RSV-infected rice plants (Fig 9), suggesting that both pathways may be blocked by viral infection. Interestingly, in a highly resistant rice variety, most of disease resistance-related genes, including LRR receptor-like protein kinase, pathogenesis-related protein, and hypersensitive reaction, were also markedly down-regulated [16], suggesting that these defense pathways may be selectively suppressed in the responses of both susceptible and resistant rice cultivars. However, in infected *Arabidopsis thaliana* displaying phenotypic and cytopathological symptoms different from those in rice plants, the defense pathways were activated by RSV [55], perhaps suggesting that monocot and dicot hosts respond in different ways to RSV. It will be instructive to investigate further the different responses of these two model plants.

### RSV may hijack chloroplasts by altering the zeaxanthin cycles

Virus infections often induce obvious symptoms in host plants and these effects must reflect a perturbation of fundamental metabolism and signal pathways in different cellular components. Our study used the rice cultivar containing the susceptibility allele STV11-S [17] and such seedlings typically respond to RSV with chlorosis, poor growth, and premature death, symptoms that are more severe than those caused by any other known rice virus. Previous ultrastructural studies of infected cells in leaves with chlorotic mottling showed that RSV typically induces four types of inclusion body and that chloroplasts have sparse, thick grana, or are even destroyed [56, 57]. In this study, 22 down-regulated mRNAs targeted by 23 up-regulated miRNAs were related to chloroplasts whereas there was only one up-regulated mRNA targeted by 4 down-regulated members of miR167-5p (Table 6). This suggests that the function of chloroplasts was seriously affected by RSV infection, which is consistent with microarray data [12, 13] and explains the typical symptoms and abnormal chloroplasts. In a recent report, iTRAQ-based quantitative proteomics analysis of rice leaves also revealed that RSV infection affected the accumulation of several proteins related to chloroplasts, among which 10 chlorophyll biosynthesis and 5 chlorophyll a/b-binding proteins were decreased by more than 3 fold in RSV infected rice plants [58]. However, only two of these 15 genes (LOC_Os08g41990 and LOC_Os01g18320 encoding Glutamate-1-semialdehyde 2,1-aminomutase and Protoporphyrinogen oxidase, respectively) were significantly down-regulated at a transcriptional level in the present study (S5 Table) which suggests that the role(s) of these genes in viral symptom development will be useful to investigate.

Two key genes (LOC_Os04g31040 and LOC_Os04g37619), encoding chloroplastic violaxanthin de-epoxidase and Zeaxanthin epoxidase have been reported to control the xanthin cycle in chloroplasts [59]. Carotenoids of the xanthophyll family act as membrane stabilizers for the lipid phase of the thylakoid membranes in chloroplasts [59]. The interaction between the xanthophyll molecules and the membrane lipids brings about a decrease in membrane fluidity, an increase in membrane thermostability and a lowered susceptibility to lipid peroxidation [59]. In our study, both genes were significantly regulated at the transcriptional level by RSV infection; violaxanthin de-epoxidase was up-regulated, but Zeaxanthin epoxidase was down-regulated. A recent quantitative proteomics study also showed that the accumulation of Zeaxanthin epoxidase was decreased more than fivefold by RSV infection [58]. These results consistently suggest that RSV infection seriously perturbs the xanthin cycle resulting in a series of chloroplast abnormities in the virus-infected plant cells. On the other hand, the xanthin cycle also controls the biosynthesis of Abscisic acid (ABA), a phytohormone playing a central role in plant growth and development as well as adaptation to a variety of stresses [60]. In rice plants, zeaxanthin is thought to be the first committed precursor and an insertion mutant in the epoxidation of zeaxanthin impairs ABA biosynthesis resulting in a wilting phenotype, one of the characteristic features of ABA-biosynthesis mutants [61]. A recent study has suggested that ABA may be involved in RSV symptom development since application of exogenous ABA prior to RSV inoculation delayed and alleviated chlorosis [62]. All these results suggest that the zeaxanthin cycle in chloroplasts may be crucially targeted by RSV infection and that this may not only impair the stabilization of chloroplast structures but also interfere with the biosynthesis of ABA, all of which may in turn contribute to symptom development. This is the first report that a plant virus can target the xanthin cycle and provides a new clue for understanding the pathogenicity mechanisms of RSV. Interestingly, some reports have showed that the viral CP and NSvc4 proteins of RSV can interact and contribute to symptom development by targeting chloroplasts [56, 63–65]. RSV CP has been confirmed to interact with host PsbP, a 23 kDa oxygen-evolving complex protein of plants, and thus enhance virus symptoms [66]. It will be interesting to investigate in depth the relationship between the zeaxanthin cycle and these viral pathogenic factors.

## Supporting Information

S1 Table The primers used for miRNA expression analysis. (PDF)

S2 Table The primers used to test the target genes. (PDF)

S3 Table The miRNAs significantly induced by RSV infection (p<0.05, Fold change ≥2, reads more than 100). (PDF)

S4 Table The miRNAs selected for qPCR assays. (PDF)

S5 Table The different expression level of genes after RSV infection (Fold change ≥2 and P<0.05). (PDF)

S6 Table The miRNAs negatively correlated with their target mRNAs. (PDF)

S7 Table The relative expression levels of mRNAs evaluated by qPCR assay. (PDF)

S8 Table The different expression levels of AP2/DREBP/EREBP/NAC-like transcript factors after RSV infection (Fold change ≥2 and P<0.05). (PDF)
