# Supplementary material for: Integrative Analysis of the microRNAome and Transcriptome Illuminates the Response of Susceptible Rice Plants to Rice Stripe Virus
Source: PLoS One. 2016 Jan 22;11(1):e0146946. doi: 10.1371/journal.pone.0146946 (PMC4723043; doi:10.1371/journal.pone.0146946)
Supplement: S1 Table — (PDF) [file pone.0146946.s001.pdf]

S1 Table. The primers used for miRNA expression analysis

miRNAs	RT Primers	Forward primers	Universal Reverse Primer
osa-miR812j	GTCGTATCCAGTGCAGGGTCCGAGGTATTCGCACTGGATACGACTGTCCA	CAAGCGTGAAGACGGATGA	CAGTGCA GGGTCCG AGGTATT
osa-miR5072	GTCGTATCCAGTGCAGGGTCCGAGGTATTCGCACTGGATACGACTGGCGA	ATATCGTGCGATTCCCCAGC	
osa-miR444a-5p	GTCGTATCCAGTGCAGGGTCCGAGGTATTCGCACTGGATACGACTATGCA	ATAGCGTGGCTAGAGGTGGC	
osa-miR444e	GTCGTATCCAGTGCAGGGTCCGAGGTATTCGCACTGGATACGACAAGCTT	ATAGCGTGTGCAGTTGCTGC	
osa-miR396c-3p	GTCGTATCCAGTGCAGGGTCCGAGGTATTCGCACTGGATACGACCTTCCC	TCAGCGTAGGTCAAGAAAGC	
osa-miR1870-5p	GTCGTATCCAGTGCAGGGTCCGAGGTATTCGCACTGGATACGACATGCCC	CCAGCGTGTGCTGAATTAGA	
osa-miR172d-5p	GTCGTATCCAGTGCAGGGTCCGAGGTATTCGCACTGGATACGACGTGAAT	ATATCGTGGCAGCACCATCA	
osa-miR171c-3p	GTCGTATCCAGTGCAGGGTCCGAGGTATTCGCACTGGATACGACGATATT	ATAGTGTGATTGAGCCGTGC	
osa-miR166d-5p	GTCGTATCCAGTGCAGGGTCCGAGGTATTCGCACTGGATACGACCCTCGA	ACAGCGTGGGAATGTTGTCT	
osa-miR159b	GTCGTATCCAGTGCAGGGTCCGAGGTATTCGCACTGGATACGACCAGAG	ACAGCGTGTTTGGATTGAAG	
osa-miR156c-3p	GTCGTATCCAGTGCAGGGTCCGAGGTATTCGCACTGGATACGACGCTGA	ACAGCGTGGCTCACTTCTCT	
osa-miR1432-5p	GTCGTATCCAGTGCAGGGTCCGAGGTATTCGCACTGGATACGACGTCGGT	ACAGCGTGATCAGGAGAGAT	
osa-miR1432-3p	GTCGTATCCAGTGCAGGGTCCGAGGTATTCGCACTGGATACGACGTCGGT	CCAGCGTGCAGGTGTCATCT	
osa-miR1429-5p	GTCGTATCCAGTGCAGGGTCCGAGGTATTCGCACTGGATACGACATGCAC	CCAGCGTGGTAATATACTAA	
osa-miR1425-3p	GTCGTATCCAGTGCAGGGTCCGAGGTATTCGCACTGGATACGACATTAA	CCAGCGTGCAGCAAGAACTG	
osa-miR1423-5p	GTCGTATCCAGTGCAGGGTCCGAGGTATTCGCACTGGATACGACCGAGCG	ATAGCGTGAGGCAACTACAC	
osa-miR1320-5p	GTCGTATCCAGTGCAGGGTCCGAGGTATTCGCACTGGATACGACCTATA	CCAGCGTGTGGAACGGAGGA	
osa-miR1863b.2	GTCGTATCCAGTGCAGGGTCCGAGGTATTCGCACTGGATACGACAGTAA	CAAGCGTGAGAGACTTGGCT	
osa-miR167a-5p	GTCGTATCCAGTGCAGGGTCCGAGGTATTCGCACTGGATACGACTAGAT	ATAGCGTGTGAAGCTGCCAG	
osa-miR156a	GTCGTATCCAGTGCAGGGTCCGAGGTATTCGCACTGGATACGACGTGCT	TCAGCGTGTGACAGAAGAGA	
