# Supplementary material for: Integrative Analysis of the microRNAome and Transcriptome Illuminates the Response of Susceptible Rice Plants to Rice Stripe Virus
Source: PLoS One. 2016 Jan 22;11(1):e0146946. doi: 10.1371/journal.pone.0146946 (PMC4723043; doi:10.1371/journal.pone.0146946)
Supplement: S2 Table — (PDF) [file pone.0146946.s002.pdf]

S2 Table. The primers used to test the target genes

Gene ID	F primer (5'-3')	R primer (5'-3')
LOC_Os09g19280	TGCGAATACCTCGGACCCT	GGCTTCATCCCAACCGTTAC
LOC_Os02g40190	CGATTCCTATCAGTATGCCC	TGACACCTTTCCACCAACAG
LOC_Os08g34740	GTTACTCGTTCGCATCTGGT	CTTCGTGGATAGCACTTGGT
LOC_Os02g33180	CAAGGTTTCTTTTGCTCTGA	TTCCACCCTCTAATCCTGTC
LOC_Os03g18850	AGCCGCTACAATGTTTCACT	GTACTTCTTCTCGTCCACCC
LOC_Os06g48590	AATCAGCTCAAGCTCATAGT	ATGCCAGGAGTGTATGGAAG
LOC_Os03g55164	CTGCTCGACGACGGCTACCG	CTCTTTCATCTGGTGAGCGC
LOC_Os03g49640	GCTGATATTTGGAGTCTTGG	TCGAAGCGGTCTATTAACAA
LOC_Os01g42400	GTCTGCCTCGAGGCCGACGG	ACGACAGTGATGTCGGCTGT
LOC_Os06g05520	CCATATCCTCCTCGTGAAAG	TTGCTTGTATTGAAGGTGGC
LOC_Os01g60490	TACATGGCTTCCTCCATGCA	GGAGACGATCACATCCTTCT
LOC_Os02g05480	TCAGCTCAAGCTCATTGTCA	GGGCACACCGGGAGTGTAGG
LOC_Os09g29840	GACAAGGAGGACTACAAAAA	GGAAGAGTAAAACCGCAACA
LOC_Os11g40970	GCACTAATTCTTGAGTATAG	GTCTCCAGCTACTCTTACCC
LOC_Os02g40200	GCTCTTTCTTGCACTCGTCT	GCAAAGCGTACTTTCTCGCT
LOC_Os01g71340	TCGCCGAGCGACGTCGTGCA	GTCCATGGTGAGGGCGATGC
LOC_Os07g35680	GATCCGGCGATGCGCGGCCG	GAGCATCACGCTCACCGCCG
LOC_Os02g57280	ACCAAGATCGGAGGAGGGAG	CCACTCACCGGAGATGATGG
LOC_Os01g53020	ATGTTCATCAATGAAGTTTA	CCTAGGGGCACTGTCATCGA
LOC_Os04g21820	TCAGCTAACATACTTTTAGA	GTAGCCAAAGGTGCCTTGTA
LOC_Os02g57280	GTGCTGGTAGCCACTGCCGA	GTCACTCTTGACGATCACTC
LOC_Os10g04570	CCACTTCGTCTTATGCCTCC	TAACTCAAAGTCCTCCTTTG
