# Supplementary material for: Integrative Analysis of the microRNAome and Transcriptome Illuminates the Response of Susceptible Rice Plants to Rice Stripe Virus
Source: PLoS One. 2016 Jan 22;11(1):e0146946. doi: 10.1371/journal.pone.0146946 (PMC4723043; doi:10.1371/journal.pone.0146946)
Supplement: S3 Table — (PDF) [file pone.0146946.s003.pdf]

S3 Table. The miRNAs significantly induced by RSV infection (p<0.05, Fold change≥2, reads more than 100)

miRNA	Sequence	Average reads		p-value	Fold change(RI/CK)
		RI	CK		
osa-miR812j	AAGACGGAUGAUUAAAGUUGGACA	1585.23 ± 126.95	584.88 ± 332.10	0.016	2.710
osa-miR812i	AAGACGGAUGAUUAAAGUUGGACA	1505.06 ± 136.95	573.68 ± 334.23	0.022	2.624
osa-miR812h	AAGACGGAUGAUUAAAGUUGGACA	1505.06 ± 136.95	573.68 ± 334.23	0.022	2.624
osa-miR812e	GACGGACGGUUAAACGUUGGAC	769.10 ± 222.95	190.83 ± 77.56	0.026	4.030
osa-miR812d	GACGGACGGUUAAACGUUGGAC	592.81 ± 59.59	73.13 ± 56.58	0.001	8.107
osa-miR812c	GACGGACGGUUAAACGUUGGAC	844.10 ± 169.39	190.83 ± 77.56	0.008	4.423
osa-miR812b	GACGGACGGUUAAACGUUGGAC	487.77 ± 81.77	81.05 ± 57.97	0.005	6.018
osa-miR812a	GACGGACGGUUAAACGUUGGAC	850.29 ± 167.22	190.83 ± 77.56	0.007	4.456
osa-miR5077	GUUCGCGUCGGGUUCACCA	889.85 ± 207.10	224.99 ± 40.88	0.011	3.955
osa-miR5072	CGAUUCCCCAGCGGAGUCGCCA	249.94 ± 32.87	45.19 ± 27.06	0.002	5.530
osa-miR444f	UGCAGUUGUUGCCUCAAGCUU	1455.90 ± 227.86	698.67 ± 188.19	0.022	2.084
osa-miR444e	UGCAGUUGCUGCCUCAAGCUU	640.67 ± 29.80	106.97 ± 10.78	0.000	5.989
osa-miR444d.2	UGCAGUUGCUGCCUCAAGCUU	630.82 ± 29.69	105.44 ± 11.70	0.000	5.983
osa-miR444c.2	UGCAGUUGUUGUCUCAAGCUU	1345.83 ± 177.66	660.34 ± 193.45	0.021	2.038
osa-miR444a-5p	GCUAGAGGUGGCAACUGCAUA	1398.91 ± 351.64	439.60 ± 81.51	0.020	3.182
osa-miR444a.2	UGCAGUUGCUGCCUCAAGCUU	643.58 ± 30.97	108.30 ± 12.08	0.000	5.943
osa-miR396c-3p	GGUCAAGAAAGCUGUGGGAAG	193.94 ± 29.42	27.27 ± 4.53	0.001	7.112
osa-miR393b	UCCAAAGGGAUCGCAUUGAUCU	134.34 ± 13.42	54.35 ± 6.66	0.002	2.472
osa-miR393	UCCAAAGGGAUCGCAUUGAUC	124.93 ± 16.38	50.35 ± 6.57	0.004	2.481
osa-miR2871a-5p	GACCGUAGAAACUAGCAUAGAAAA	1242.10 ± 430.65	37.83 ± 12.95	0.017	32.833
osa-miR1883a	ACCUGUGACGGGCCGAGAAUGGAA	258.90 ± 92.82	60.52 ± 11.71	0.040	4.278
osa-miR1870-5p	UGCUGAAUUAGACCUAGUGGGCAU	2165.33 ± 279.45	156.41 ± 19.10	0.001	13.844
osa-miR1863b.2	AGAGACUUGGCUGAUGCAUUACU	159.81 ± 41.39	403.72 ± 32.84	0.003	0.396

osa-miR1861j	CGGUCUUGAGGCAGGAACUGAG	169.62 ± 44.11	11.44 ± 2.61	0.007	14.829
osa-miR1861h	CGGUCUUGAGGCAGGAACUGAG	169.62 ± 44.11	11.44 ± 2.61	0.007	14.829
osa-miR172d-5p	GCAGCACCAUCAAGAUUCAC	667.58 ± 22.38	59.76 ± 18.29	0.000	11.171
osa-miR172d-3p	AGAAUCUUGAUGAUGCUGCAU	15880.86 ± 2505.18	5314.54 ± 2426.61	0.013	2.988
osa-miR172c	UGAAUCUUGAUGAUGCUGCAC	548.03 ± 131.03	69.31 ± 58.14	0.009	7.907
osa-miR171c-3p	UGAUUGAGCCGUGCCAAUAUC	242.23 ± 6.77	28.37 ± 17.26	0.000	8.539
osa-miR167i-5p	UGAAGCUGCCAGCAUGAUCUG	333.64 ± 53.00	933.74 ± 195.36	0.014	0.357
osa-miR167i-3p	AGAUCAUGUUGCAGCUUCACU	437.48 ± 41.59	63.99 ± 15.09	0.000	6.837
osa-miR167h-3p	AGGUCAUGCUGUAGUUUCAUC	1747.73 ± 128.09	15.80 ± 7.34	0.000	110.619
osa-miR167e-5p	UGAAGCUGCCAGCAUGAUCUG	447.77 ± 75.95	962.22 ± 197.36	0.026	0.465
osa-miR167e-3p	AGAUCAUGUUGCAGCUUCACU	437.48 ± 41.59	63.99 ± 15.09	0.000	6.837
osa-miR167c-5p	UGAAGCUGCCAGCAUGAUCUA	597.39 ± 242.72	3659.87 ± 937.65	0.011	0.163
osa-miR167a-5p	UGAAGCUGCCAGCAUGAUCUA	597.39 ± 242.72	3659.87 ± 937.65	0.011	0.163
osa-miR167a-3p	AUCAUGCAUGACAGCCUCAUUU	97.33 ± 31.56	11.38 ± 6.51	0.020	8.553
osa-miR166d-5p	GGAAUGUUGUCUGGCUCGAGG	1664.15 ± 212.45	44.99 ± 13.18	0.000	36.989
osa-miR166b-5p	GGAAUGUUGUCUGGCUCGGGG	165.07 ± 6.55	5.31 ± 2.16	0.000	31.089
osa-miR164f	UGGAGAAGCAGGGCACGUGCA	9567.50 ± 654.79	4724.00 ± 86.88	0.000	2.025
osa-miR164b	UGGAGAAGCAGGGCACGUGCA	10898.81 ± 1662.59	5057.07 ± 554.40	0.009	2.155
osa-miR164a	UGGAGAAGCAGGGCACGUGCA	10900.50 ± 1662.18	5057.86 ± 555.52	0.009	2.155
osa-miR160d-3p	GCGUGCGAGGAGCCAAGCAUG	144.64 ± 10.06	0.00 ± 0.00	0.000	NA
osa-miR160c-3p	GCGUGCACGGAGCCAAGCAUA	120.97 ± 8.87	0.00 ± 0.00	0.000	NA
osa-miR160b-3p	GCGUGCAAGGAGCCAAGCAUG	141.85 ± 24.04	14.09 ± 7.79	0.002	10.066
osa-miR160a-3p	GCGUGCAAGGAGCCAAGCAUG	141.85 ± 24.04	14.09 ± 7.79	0.002	10.066
osa-miR159e	AUUGGAUUGAAGGGAGCUCCU	332.15 ± 83.15	73.18 ± 6.65	0.012	4.539
osa-miR159d	AUUGGAUUGAAGGGAGCUCCG	379.25 ± 145.58	74.51 ± 3.91	0.042	5.090
osa-miR159b	UUUGGAUUGAAGGGAGCUCUG	8706.52 ± 416.03	2943.48 ± 387.62	0.000	2.958
osa-miR159a.2	UUGCAUGCCCCAGGAGCUGCA	6519.31 ± 286.06	783.41 ± 94.21	0.000	8.322

osa-miR159a.1	UUUGGAUUGAAGGGAGCUCUG	8373.18 ± 682.39	2276.82 ± 757.04	0.001	3.678
osa-miR156k	UGACAGAAGAGAGAGAGCACA	11457.92 ± 775.60	176994.42 ± 12030.08	0.000	0.065
osa-miR156j-5p	UGACAGAAGAGAGUGAGCAC	2922.48 ± 335.43	28265.84 ± 4356.10	0.001	0.103
osa-miR156j-3p	GCUCGCUCCUCUUUCUGUCAGC	115.68 ± 4.78	6.04 ± 1.41	0.000	19.154
osa-miR156i	UGACAGAAGAGAGUGAGCAC	19179.03 ± 4823.24	208953.80 ± 6546.05	0.000	0.092
osa-miR156h-5p	UGACAGAAGAGAGUGAGCAC	2921.84 ± 334.58	28266.54 ± 4356.14	0.001	0.103
osa-miR156g-3p	GCUCACUUCUCUCUCUGUCAGC	153.24 ± 45.70	0.00 ± 0.00	0.009	NA
osa-miR156f-5p	UGACAGAAGAGAGUGAGCAC	2921.84 ± 334.58	28266.54 ± 4356.14	0.001	0.103
osa-miR156e	UGACAGAAGAGAGUGAGCAC	19179.03 ± 4823.24	208953.80 ± 6546.05	0.000	0.092
osa-miR156d	UGACAGAAGAGAGUGAGCAC	24960.15 ± 4648.45	230533.14 ± 30811.82	0.001	0.108
osa-miR156c-3p	GCUCACUUCUCUCUCUGUCAGC	153.24 ± 45.70	0.00 ± 0.00	0.009	NA
osa-miR156a	UGACAGAAGAGAGUGAGCAC	19512.37 ± 4757.86	208956.99 ± 6548.31	0.000	0.093
osa-miR1432-5p	AUCAGGAGAGAUGACACCGAC	9330.33 ± 152.16	341.50 ± 271.41	0.000	27.321
osa-miR1432-3p	CAGGUGUCAUCUCCCCUGAAC	236.19 ± 34.58	0.00 ± 0.00	0.001	NA
osa-miR1429-5p	GUAAUAUACUAAUCCGUGCAU	214.67 ± 30.13	58.09 ± 24.61	0.005	3.695
osa-miR1425-3p	CAGCAAGAACUGGAUCUUAAU	15480.54 ± 2450.08	677.46 ± 138.32	0.001	22.851
osa-miR1423-5p	AGGCAACUACACGUUGGGCGCUCG	266.94 ± 23.28	59.65 ± 14.68	0.000	4.475
osa-miR1320-5p	UGGAACGGAGGAAUUUUAUAG	4257.84 ± 596.40	263.52 ± 70.43	0.001	16.158
osa-miR1320-3p	UGUAAAAUUCAUUCGUUCCAA	374.13 ± 45.95	41.98 ± 10.23	0.001	8.911
