# Supplementary material for: Integrative Analysis of the microRNAome and Transcriptome Illuminates the Response of Susceptible Rice Plants to Rice Stripe Virus
Source: PLoS One. 2016 Jan 22;11(1):e0146946. doi: 10.1371/journal.pone.0146946 (PMC4723043; doi:10.1371/journal.pone.0146946)
Supplement: S4 Table — (PDF) [file pone.0146946.s004.pdf]

S4 Table. The miRNAs selected for qPCR assays

miRNAs	miRNA sequences (5'-3')	RI		CK		p-value	Fold change
		CT values	Std. Dev.	CT values	Std. Dev.		
osa-miR812j	AAGACGGAUGAUUAAAGUUGGACA	24.3	0.0286	25.16	0.0452	0.008	1.53
osa-miR5072	CGAUUCCCCAGCGGAGUCGCCA	21.5	0.0353	24.24	0.0140	0.001	5.72
osa-miR444e	UGCAGUUGCUGCCUCAAGCUU	22.1	0.0690	23.57	0.0558	0.009	2.37
osa-miR444a-5p	GCUAGAGGUGGCAACUGCAUA	19.7	0.0366	21.15	0.0513	0.006	2.38
osa-miR396c-3p	GGUCAAGAAAGCUGUGGGAAG	18.2	0.0799	21.21	0.1489	0.000	6.90
osa-miR1870-5p	UGCUGAAUUAGACCUAGUGGGCAU	20.7	0.0633	22.06	0.0629	0.039	2.11
osa-miR1863b.2	AGAGACUUGGCUGAUGCAUUACU	22.4	0.0633	20.50	0.0391	0.002	0.33
osa-miR172d-5p	GCAGCACCAUCAAGAUUCAC	27.0	0.0683	29.25	0.0391	0.002	4.00
osa-miR171c-3p	UGAUUGAGCCGUGCCAAUAUC	18.3	0.0866	21.77	0.0093	0.002	1.58
osa-miR167a-5p	UGAAGCUGCCAGCAUGAUCUA	21.7	0.0690	19.20	0.0513	0.004	0.53
osa-miR166d-5p	GGAAUGUUGUCUGGCUCGAGG	19.62	0.0267	25.84	0.0799	0.000	62.71
osa-miR159b	UUUGGAUUGAAGGGAGCUCUG	19.02	0.0160	22.47	0.0506	0.001	9.25
osa-miR156c-3p	GCUCACUUCUCUCUCUGUCAGC	18.80	0.1000	19.46	0.0792	0.007	1.58
osa-miR156a	UGACAGAAGAGAGUGAGCAC	25.6	0.0353	23.50	0.1489	0.001	0.68
osa-miR1432-5p	AUCAGGAGAGAUGACACCGAC	21.8	0.0516	22.87	0.0515	0.000	1.83
osa-miR1432-3p	CAGGUGUCAUCUCCCCUGAAC	20.2	0.0738	22.35	0.0866	0.002	3.73
osa-miR1429-5p	GUAAUAUACUAAUCCGUGCAU	24.3	0.0633	26.78	0.2552	0.001	4.75
osa-miR1425-3p	CAGCAAGAACUGGAUCUUAAU	18.6	0.0429	24.60	0.0125	0.000	53.84
osa-miR1423-5p	AGGCAACUACACGUUGGGCGCUCG	22.5	0.0633	23.53	0.0229	0.014	1.73
osa-miR1320-5p	UGGAACGGAGGAAUUUUAUAG	17.5	0.0161	20.49	0.0267	0.003	6.63
