# Supplementary material for: Integrative Analysis of the microRNAome and Transcriptome Illuminates the Response of Susceptible Rice Plants to Rice Stripe Virus
Source: PLoS One. 2016 Jan 22;11(1):e0146946. doi: 10.1371/journal.pone.0146946 (PMC4723043; doi:10.1371/journal.pone.0146946)
Supplement: S5 Table — (PDF) [file pone.0146946.s005.pdf]

S5 Table. The different expression level of genes after RSV infection (Fold change≥2 and P<0.05)

Gene ID	Average reads		p-value	Fold change (RI/CK)
	RI FPKM values	CK FPKM values		
LOC_Os01g01620	36.672 ± 2.349	17.529 ± 2.115	0.001	2.09
LOC_Os01g01660	580.402 ± 76.756	19.153 ± 2.992	0.009	30.30
LOC_Os01g01700	12.921 ± 0.630	27.451 ± 1.848	0.004	0.47
LOC_Os01g01760	2.146 ± 0.501	23.391 ± 4.283	0.019	0.09
LOC_Os01g01870	17.515 ± 0.543	7.972 ± 0.482	0.000	2.20
LOC_Os01g02390	9.825 ± 0.492	20.236 ± 1.493	0.006	0.49
LOC_Os01g02400	8.024 ± 1.021	22.551 ± 0.689	0.000	0.36
LOC_Os01g02570	4.351 ± 1.033	15.875 ± 1.663	0.002	0.27
LOC_Os01g02600	4.826 ± 0.996	12.294 ± 0.537	0.002	0.39
LOC_Os01g02700	9.195 ± 2.173	20.646 ± 2.436	0.008	0.45
LOC_Os01g02710	23.453 ± 3.638	60.131 ± 11.642	0.037	0.39
LOC_Os01g02780	2.309 ± 0.514	21.014 ± 2.193	0.005	0.11
LOC_Os01g02830	5.046 ± 0.285	10.599 ± 1.042	0.012	0.48
LOC_Os01g02900	6.982 ± 0.468	23.214 ± 2.916	0.014	0.30
LOC_Os01g03040	11.389 ± 0.450	24.914 ± 3.738	0.035	0.46
LOC_Os01g03320	267.491 ± 6.103	32.925 ± 1.987	0.000	8.12
LOC_Os01g03340	10.318 ± 0.924	3.053 ± 1.815	0.015	3.38
LOC_Os01g03680	18.918 ± 1.411	3.434 ± 0.532	0.002	5.51
LOC_Os01g03760	7.778 ± 0.660	17.625 ± 0.184	0.001	0.44
LOC_Os01g04010	116.999 ± 22.796	315.121 ± 46.159	0.013	0.37
LOC_Os01g04050	55.331 ± 6.709	8.588 ± 1.702	0.007	6.44
LOC_Os01g04330	77.716 ± 10.558	14.563 ± 1.562	0.012	5.34
LOC_Os01g04380	29.717 ± 8.493	3.602 ± 0.487	0.049	8.25
LOC_Os01g05064	13.477 ± 0.792	6.344 ± 0.321	0.002	2.12
LOC_Os01g05540	3.043 ± 0.318	13.411 ± 0.542	0.000	0.23
LOC_Os01g05630	9.780 ± 1.837	26.131 ± 3.263	0.007	0.37
LOC_Os01g06750	3.733 ± 0.584	26.096 ± 5.719	0.030	0.14
LOC_Os01g06876	0.554 ± 0.315	11.407 ± 2.004	0.015	0.05
LOC_Os01g07090	9.921 ± 0.576	21.790 ± 1.437	0.003	0.46
LOC_Os01g07370	16.048 ± 1.013	6.897 ± 0.190	0.005	2.33
LOC_Os01g07590	6.970 ± 0.282	15.182 ± 1.564	0.015	0.46
LOC_Os01g08860	1387.597 ± 65.497	646.775 ± 51.838	0.000	2.15
LOC_Os01g09190	34.640 ± 2.269	111.347 ± 15.281	0.018	0.31
LOC_Os01g09220	354.509 ± 30.455	4.252 ± 1.127	0.004	83.37
LOC_Os01g09620	13.780 ± 2.287	4.041 ± 0.620	0.020	3.41
LOC_Os01g09770	22.680 ± 0.407	4.624 ± 0.400	0.000	4.90
LOC_Os01g09800	5.365 ± 0.701	11.612 ± 0.830	0.001	0.46
LOC_Os01g09870	11.050 ± 1.462	2.133 ± 1.180	0.003	5.18
LOC_Os01g10400	1815.489 ± 58.413	3784.225 ± 26.332	0.000	0.48
LOC_Os01g11160	23.746 ± 2.232	69.002 ± 1.660	0.000	0.34
LOC_Os01g11590	5.638 ± 0.260	14.640 ± 2.178	0.027	0.39

LOC_Os01g12400	8.204 ± 0.269	16.846 ± 0.109	0.000	0.49
LOC_Os01g12920	20.987 ± 0.890	47.382 ± 7.144	0.033	0.44
LOC_Os01g13570	31.975 ± 2.866	79.278 ± 11.061	0.021	0.40
LOC_Os01g13690	74.515 ± 4.885	12.059 ± 1.901	0.001	6.18
LOC_Os01g14410	15.252 ± 1.368	30.542 ± 3.650	0.018	0.50
LOC_Os01g15270	594.805 ± 71.605	263.700 ± 13.532	0.020	2.26
LOC_Os01g16430	27.395 ± 1.470	11.163 ± 1.606	0.000	2.45
LOC_Os01g16620	23.468 ± 1.497	47.274 ± 0.096	0.002	0.50
LOC_Os01g17050	19.962 ± 4.099	2.310 ± 0.354	0.025	8.64
LOC_Os01g17150	79.546 ± 2.895	168.098 ± 9.861	0.004	0.47
LOC_Os01g18320	7.361 ± 0.354	16.017 ± 0.562	0.000	0.46
LOC_Os01g19020	11.172 ± 0.954	0.646 ± 0.241	0.003	17.30
LOC_Os01g20890	4.971 ± 0.213	10.148 ± 0.588	0.003	0.49
LOC_Os01g20910	25.512 ± 6.506	66.374 ± 5.383	0.003	0.38
LOC_Os01g25540	26.763 ± 4.564	6.443 ± 1.390	0.017	4.15
LOC_Os01g26240	4.354 ± 0.183	13.371 ± 1.429	0.011	0.33
LOC_Os01g28790	73.799 ± 5.253	24.087 ± 4.285	0.001	3.06
LOC_Os01g32670	18.489 ± 1.483	2.610 ± 1.445	0.000	7.08
LOC_Os01g34790	42.717 ± 8.108	116.381 ± 11.049	0.002	0.37
LOC_Os01g35330	12.858 ± 0.627	133.339 ± 21.177	0.015	0.10
LOC_Os01g36294	1.853 ± 0.234	11.081 ± 2.101	0.024	0.17
LOC_Os01g36850	0.029 ± 0.042	10.970 ± 0.385	0.001	0.00
LOC_Os01g36890	5.393 ± 0.142	12.202 ± 1.025	0.010	0.44
LOC_Os01g36920	7.015 ± 0.664	14.627 ± 0.316	0.001	0.48
LOC_Os01g37040	14.251 ± 1.646	5.121 ± 1.022	0.005	2.78
LOC_Os01g37130	19.720 ± 1.037	42.284 ± 1.637	0.000	0.47
LOC_Os01g37630	6.240 ± 0.462	16.742 ± 1.211	0.003	0.37
LOC_Os01g37750	34.865 ± 4.840	16.122 ± 2.542	0.016	2.16
LOC_Os01g37820	6.128 ± 1.262	14.304 ± 3.061	0.048	0.43
LOC_Os01g37920	77.277 ± 2.051	18.903 ± 2.015	0.000	4.09
LOC_Os01g37950	276.057 ± 15.747	104.175 ± 17.736	0.001	2.65
LOC_Os01g38980	42.740 ± 3.493	15.045 ± 1.967	0.002	2.84
LOC_Os01g40070	67.951 ± 9.148	12.037 ± 0.338	0.013	5.65
LOC_Os01g41420	30.007 ± 0.173	0.293 ± 0.210	0.000	102.47
LOC_Os01g42790	4.101 ± 0.496	36.114 ± 3.010	0.004	0.11
LOC_Os01g43070	5.045 ± 0.241	11.851 ± 2.014	0.039	0.43
LOC_Os01g43120	20.776 ± 0.417	44.064 ± 4.759	0.020	0.47
LOC_Os01g43270	14.488 ± 1.902	38.756 ± 2.513	0.001	0.37
LOC_Os01g43430	10.410 ± 1.081	4.763 ± 0.475	0.009	2.19
LOC_Os01g43750	51.920 ± 2.036	19.846 ± 0.689	0.001	2.62
LOC_Os01g43844	5.231 ± 0.347	13.616 ± 0.888	0.002	0.38
LOC_Os01g43851	2.383 ± 0.069	10.004 ± 0.722	0.004	0.24
LOC_Os01g44120	17.747 ± 2.239	5.479 ± 0.451	0.014	3.24
LOC_Os01g44390	7.561 ± 0.959	23.315 ± 4.394	0.032	0.32
LOC_Os01g44980	6.408 ± 0.286	13.518 ± 1.870	0.030	0.47

LOC_Os01g45530	5.694 ± 0.427	12.282 ± 1.028	0.005	0.46
LOC_Os01g45914	14.493 ± 1.521	5.572 ± 0.157	0.014	2.60
LOC_Os01g46980	11.412 ± 1.101	25.450 ± 0.673	0.000	0.45
LOC_Os01g47330	306.917 ± 31.154	681.780 ± 6.963	0.002	0.45
LOC_Os01g47370	2.937 ± 1.109	10.308 ± 0.404	0.006	0.28
LOC_Os01g47450	5.364 ± 0.107	15.307 ± 1.768	0.015	0.35
LOC_Os01g47730	48.560 ± 6.696	19.882 ± 1.950	0.020	2.44
LOC_Os01g47760	67.858 ± 4.205	20.934 ± 2.970	0.000	3.24
LOC_Os01g48200	10.283 ± 1.850	21.154 ± 0.651	0.008	0.49
LOC_Os01g48446	17.712 ± 1.693	4.216 ± 0.573	0.004	4.20
LOC_Os01g48610	6.718 ± 0.564	28.479 ± 1.245	0.000	0.24
LOC_Os01g48620	13.312 ± 0.746	48.094 ± 4.426	0.007	0.28
LOC_Os01g48640	26.041 ± 2.086	78.022 ± 5.501	0.002	0.33
LOC_Os01g48770	13.114 ± 0.182	29.200 ± 3.817	0.027	0.45
LOC_Os01g48950	17.030 ± 1.972	53.199 ± 3.829	0.001	0.32
LOC_Os01g49210	20.831 ± 1.780	7.503 ± 1.518	0.001	2.78
LOC_Os01g49750	0.346 ± 0.183	31.337 ± 3.417	0.006	0.01
LOC_Os01g49830	6.585 ± 0.455	16.588 ± 1.448	0.006	0.40
LOC_Os01g50030	12.758 ± 0.795	36.066 ± 3.872	0.011	0.35
LOC_Os01g50032	10.636 ± 0.795	28.403 ± 5.675	0.045	0.37
LOC_Os01g50410	15.219 ± 3.617	1.072 ± 0.332	0.030	14.19
LOC_Os01g50420	16.433 ± 2.839	0.239 ± 0.036	0.015	68.73
LOC_Os01g50770	9.283 ± 0.875	21.609 ± 0.634	0.000	0.43
LOC_Os01g50849	6.357 ± 0.367	14.511 ± 0.566	0.000	0.44
LOC_Os01g50930	7.826 ± 0.496	16.667 ± 1.024	0.002	0.47
LOC_Os01g51390	10.449 ± 1.304	21.723 ± 1.343	0.001	0.48
LOC_Os01g51410	51.786 ± 0.684	120.567 ± 12.601	0.016	0.43
LOC_Os01g51570	27.245 ± 4.581	74.977 ± 4.124	0.000	0.36
LOC_Os01g52010	8.436 ± 1.810	29.047 ± 2.067	0.000	0.29
LOC_Os01g52500	37.951 ± 1.701	90.599 ± 7.017	0.006	0.42
LOC_Os01g53020	50.289 ± 1.284	129.284 ± 7.299	0.003	0.39
LOC_Os01g53150	10.648 ± 0.087	22.204 ± 1.380	0.007	0.48
LOC_Os01g53920	12.123 ± 0.176	32.702 ± 2.415	0.007	0.37
LOC_Os01g54050	4.641 ± 0.117	10.765 ± 0.863	0.009	0.43
LOC_Os01g54350	6.239 ± 0.376	14.448 ± 1.485	0.012	0.43
LOC_Os01g54880	4.364 ± 0.504	10.038 ± 1.638	0.030	0.43
LOC_Os01g55450	4.773 ± 0.515	10.482 ± 1.436	0.020	0.46
LOC_Os01g55510	23.914 ± 5.917	3.434 ± 0.488	0.038	6.96
LOC_Os01g55870	6.038 ± 0.241	15.189 ± 2.503	0.034	0.40
LOC_Os01g55950	17.835 ± 0.900	42.415 ± 2.503	0.002	0.42
LOC_Os01g56350	15.621 ± 1.352	32.378 ± 4.413	0.025	0.48
LOC_Os01g57599	6.558 ± 0.607	43.826 ± 4.905	0.008	0.15
LOC_Os01g57958	48.813 ± 4.507	3.588 ± 1.381	0.003	13.61
LOC_Os01g57962	24.669 ± 3.591	11.546 ± 1.179	0.026	2.14
LOC_Os01g57964	10.884 ± 1.290	4.335 ± 0.248	0.016	2.51

LOC_Os01g58000	14.824 ± 0.796	3.116 ± 0.952	0.000	4.76
LOC_Os01g58020	122.907 ± 15.280	36.284 ± 3.862	0.011	3.39
LOC_Os01g58039	12.107 ± 1.625	3.918 ± 0.709	0.010	3.09
LOC_Os01g58049	28.050 ± 5.514	9.131 ± 1.637	0.031	3.07
LOC_Os01g58100	16.961 ± 0.920	0.187 ± 0.111	0.001	90.68
LOC_Os01g58420	31.267 ± 3.130	8.477 ± 2.614	0.002	3.69
LOC_Os01g58470	13.887 ± 1.791	33.609 ± 3.887	0.009	0.41
LOC_Os01g58620	40.940 ± 1.721	12.878 ± 1.304	0.000	3.18
LOC_Os01g60280	5.100 ± 0.338	11.526 ± 0.414	0.000	0.44
LOC_Os01g60640	22.408 ± 2.534	3.777 ± 0.277	0.009	5.93
LOC_Os01g61044	12.008 ± 0.140	1.223 ± 0.090	0.000	9.82
LOC_Os01g61670	16.309 ± 1.882	6.519 ± 0.816	0.009	2.50
LOC_Os01g62670	10.131 ± 1.131	1.835 ± 0.675	0.002	5.52
LOC_Os01g63400	10.186 ± 0.657	20.589 ± 1.667	0.006	0.49
LOC_Os01g63410	29.139 ± 2.560	60.302 ± 3.001	0.000	0.48
LOC_Os01g64360	28.081 ± 3.258	7.225 ± 0.612	0.010	3.89
LOC_Os01g64470	52.627 ± 6.058	8.218 ± 1.537	0.006	6.40
LOC_Os01g64680	8.198 ± 0.718	19.359 ± 2.331	0.015	0.42
LOC_Os01g65230	6.942 ± 0.377	15.020 ± 1.353	0.010	0.46
LOC_Os01g67134	28.622 ± 0.971	62.437 ± 3.662	0.004	0.46
LOC_Os01g67810	11.304 ± 0.409	4.409 ± 0.307	0.000	2.56
LOC_Os01g68140	7.598 ± 0.202	17.950 ± 3.076	0.041	0.42
LOC_Os01g68269	59.335 ± 13.482	143.679 ± 21.004	0.013	0.41
LOC_Os01g68300	896.499 ± 70.902	2439.812 ± 167.582	0.002	0.37
LOC_Os01g68324	9.247 ± 0.603	18.565 ± 0.530	0.000	0.50
LOC_Os01g68450	36.447 ± 1.350	85.541 ± 7.976	0.011	0.43
LOC_Os01g68650	13.938 ± 2.816	0.788 ± 0.412	0.020	17.68
LOC_Os01g68770	37.759 ± 3.031	16.367 ± 1.126	0.005	2.31
LOC_Os01g69120	21.399 ± 0.934	44.118 ± 1.944	0.001	0.49
LOC_Os01g69840	87.498 ± 7.326	272.372 ± 13.747	0.000	0.32
LOC_Os01g70110	5.157 ± 0.631	14.946 ± 0.516	0.000	0.35
LOC_Os01g70120	7.731 ± 1.255	20.584 ± 3.043	0.016	0.38
LOC_Os01g70130	8.666 ± 0.889	17.569 ± 1.225	0.002	0.49
LOC_Os01g70460	11.100 ± 0.873	26.048 ± 1.280	0.000	0.43
LOC_Os01g70710	25.865 ± 1.083	52.033 ± 5.924	0.022	0.50
LOC_Os01g70720	4.097 ± 0.445	13.642 ± 0.935	0.001	0.30
LOC_Os01g70790	189.786 ± 3.941	31.022 ± 3.686	0.000	6.12
LOC_Os01g71340	9.590 ± 0.835	35.518 ± 3.575	0.007	0.27
LOC_Os01g71630	23.011 ± 1.170	51.914 ± 4.966	0.011	0.44
LOC_Os01g71720	8.609 ± 0.647	22.800 ± 2.789	0.015	0.38
LOC_Os01g72100	69.451 ± 3.284	15.453 ± 1.086	0.001	4.49
LOC_Os01g72360	10.003 ± 2.124	1.041 ± 0.154	0.026	9.61
LOC_Os01g72530	98.168 ± 22.459	10.959 ± 1.490	0.031	8.96
LOC_Os01g72800	8.931 ± 0.304	28.783 ± 2.330	0.006	0.31
LOC_Os01g72900	17.137 ± 1.986	4.810 ± 2.070	0.004	3.56

LOC_Os01g72910	12.740 ± 2.239	5.202 ± 2.042	0.025	2.45
LOC_Os01g73250	168.159 ± 9.731	730.147 ± 115.009	0.020	0.23
LOC_Os01g73450	34.774 ± 3.273	137.031 ± 4.496	0.000	0.25
LOC_Os01g73970	14.773 ± 0.791	35.807 ± 1.206	0.000	0.41
LOC_Os01g74040	40.135 ± 4.087	6.484 ± 1.029	0.005	6.19
LOC_Os01g74160	19.375 ± 1.230	118.124 ± 4.928	0.001	0.16
LOC_Os01g74300	84.380 ± 5.731	387.054 ± 44.973	0.010	0.22
LOC_Os01g74410	12.216 ± 0.665	45.584 ± 2.326	0.001	0.27
LOC_Os01g74540	4.231 ± 0.387	10.062 ± 1.355	0.020	0.42
LOC_Os02g01220	16.795 ± 0.795	7.781 ± 0.694	0.000	2.16
LOC_Os02g02120	10.466 ± 0.615	32.674 ± 3.529	0.011	0.32
LOC_Os02g02390	13.516 ± 1.054	28.593 ± 1.672	0.001	0.47
LOC_Os02g02770	5.850 ± 0.573	12.207 ± 1.190	0.007	0.48
LOC_Os02g02830	22.599 ± 2.216	56.110 ± 6.780	0.013	0.40
LOC_Os02g02860	5.122 ± 0.614	13.879 ± 0.868	0.001	0.37
LOC_Os02g03330	8.886 ± 0.266	19.005 ± 1.815	0.014	0.47
LOC_Os02g03370	5.317 ± 0.606	12.331 ± 0.319	0.001	0.43
LOC_Os02g03710	342.529 ± 79.639	73.451 ± 12.144	0.038	4.66
LOC_Os02g03960	19.185 ± 2.850	51.357 ± 4.966	0.003	0.37
LOC_Os02g04130	23.432 ± 1.263	1.510 ± 0.437	0.001	15.52
LOC_Os02g05470	16.827 ± 1.299	38.106 ± 4.724	0.018	0.44
LOC_Os02g05790	9.095 ± 1.089	18.201 ± 0.480	0.002	0.50
LOC_Os02g06270	4.191 ± 0.612	10.287 ± 0.730	0.001	0.41
LOC_Os02g08440	90.279 ± 11.876	35.460 ± 3.620	0.016	2.55
LOC_Os02g09490	3.514 ± 0.479	10.060 ± 0.142	0.001	0.35
LOC_Os02g09990	14.796 ± 1.140	4.362 ± 0.629	0.001	3.39
LOC_Os02g10810	7.900 ± 0.913	24.404 ± 4.166	0.026	0.32
LOC_Os02g11720	7.117 ± 1.100	18.178 ± 3.405	0.034	0.39
LOC_Os02g13060	59.058 ± 7.109	146.097 ± 5.789	0.000	0.40
LOC_Os02g13130	35.775 ± 0.367	77.024 ± 5.019	0.007	0.46
LOC_Os02g13510	57.899 ± 1.915	148.689 ± 6.431	0.001	0.39
LOC_Os02g15110	4.086 ± 0.789	11.497 ± 1.356	0.005	0.36
LOC_Os02g15120	16.922 ± 0.833	41.130 ± 3.430	0.007	0.41
LOC_Os02g15860	11.162 ± 3.066	2.596 ± 1.604	0.039	4.30
LOC_Os02g16490	4.393 ± 0.555	10.036 ± 0.365	0.001	0.44
LOC_Os02g16620	23.424 ± 0.384	11.583 ± 0.489	0.000	2.02
LOC_Os02g17470	7.154 ± 0.102	19.404 ± 2.201	0.016	0.37
LOC_Os02g17710	4.860 ± 0.442	13.375 ± 1.922	0.020	0.36
LOC_Os02g18070	4.236 ± 0.285	20.353 ± 1.230	0.002	0.21
LOC_Os02g18784	4.900 ± 1.075	16.922 ± 2.737	0.015	0.29
LOC_Os02g19150	6.337 ± 0.529	19.233 ± 0.903	0.000	0.33
LOC_Os02g20360	13.096 ± 1.926	119.162 ± 3.679	0.000	0.11
LOC_Os02g20934	5.597 ± 0.906	14.109 ± 1.426	0.004	0.40
LOC_Os02g24100	4.737 ± 1.959	11.448 ± 0.819	0.026	0.41
LOC_Os02g24598	20.148 ± 1.685	8.162 ± 3.141	0.017	2.47

LOC_Os02g24604	13.983 ± 2.630	3.692 ± 1.887	0.014	3.79
LOC_Os02g24628	10.165 ± 2.908	0.440 ± 0.321	0.040	23.11
LOC_Os02g24634	43.572 ± 6.970	20.599 ± 1.222	0.040	2.12
LOC_Os02g26700	128.824 ± 10.132	44.181 ± 2.863	0.004	2.92
LOC_Os02g28720	10.973 ± 0.689	5.280 ± 0.956	0.003	2.08
LOC_Os02g30470	17.010 ± 4.736	1.276 ± 0.972	0.038	13.33
LOC_Os02g32580	28.540 ± 2.860	5.279 ± 1.638	0.002	5.41
LOC_Os02g32590	24.325 ± 0.517	9.573 ± 0.511	0.000	2.54
LOC_Os02g33020	8.045 ± 0.327	27.918 ± 1.218	0.001	0.29
LOC_Os02g33070	42.197 ± 4.474	95.240 ± 10.355	0.009	0.44
LOC_Os02g33110	13.708 ± 1.407	33.868 ± 1.851	0.000	0.40
LOC_Os02g33680	79.056 ± 10.226	11.599 ± 0.864	0.011	6.82
LOC_Os02g34580	1.918 ± 0.138	23.461 ± 1.615	0.003	0.08
LOC_Os02g35329	70.141 ± 7.353	25.878 ± 1.012	0.012	2.71
LOC_Os02g35347	38.716 ± 6.537	10.509 ± 0.860	0.024	3.68
LOC_Os02g35365	34.473 ± 6.235	8.611 ± 1.499	0.023	4.00
LOC_Os02g35440	9.866 ± 1.568	21.103 ± 0.840	0.003	0.47
LOC_Os02g35720	4.794 ± 0.969	10.146 ± 0.738	0.004	0.47
LOC_Os02g35820	13.877 ± 0.487	5.448 ± 0.916	0.001	2.55
LOC_Os02g36330	11.501 ± 0.906	5.673 ± 0.297	0.007	2.03
LOC_Os02g36940	69.455 ± 4.420	152.250 ± 7.044	0.000	0.46
LOC_Os02g37090	5.390 ± 0.336	13.057 ± 0.826	0.002	0.41
LOC_Os02g37300	129.222 ± 26.123	49.288 ± 4.398	0.046	2.62
LOC_Os02g37330	44.570 ± 5.988	108.974 ± 11.147	0.005	0.41
LOC_Os02g38160	20.814 ± 0.648	48.493 ± 2.755	0.003	0.43
LOC_Os02g38170	11.358 ± 0.978	33.516 ± 1.560	0.000	0.34
LOC_Os02g38386	13.606 ± 2.464	3.025 ± 0.490	0.023	4.50
LOC_Os02g38920	6.597 ± 0.906	16.904 ± 0.698	0.000	0.39
LOC_Os02g39330	24.433 ± 6.375	52.961 ± 3.808	0.010	0.46
LOC_Os02g39660	9.306 ± 1.980	27.743 ± 1.977	0.001	0.34
LOC_Os02g40040	7.725 ± 0.260	19.679 ± 2.285	0.017	0.39
LOC_Os02g40130	0.000 ± 0.000	28.492 ± 3.441	0.007	0.00
LOC_Os02g40180	0.014 ± 0.020	17.154 ± 1.188	0.002	0.00
LOC_Os02g40190	10.12±1.18	0.07 ± 0.1	0.000	0.01
LOC_Os02g40200	0.000 ± 0.000	30.785 ± 0.554	0.000	0.00
LOC_Os02g40240	0.278 ± 0.071	1110.210 ± 31.547	0.000	0.00
LOC_Os02g40500	2.731 ± 0.192	10.758 ± 1.856	0.025	0.25
LOC_Os02g40690	6.765 ± 0.544	24.703 ± 4.232	0.025	0.27
LOC_Os02g40700	5.089 ± 0.303	11.080 ± 1.669	0.033	0.46
LOC_Os02g40830	9.025 ± 0.246	18.058 ± 1.784	0.017	0.50
LOC_Os02g41590	4.888 ± 0.492	13.291 ± 0.811	0.001	0.37
LOC_Os02g41680	13.021 ± 1.343	2.735 ± 0.529	0.004	4.76
LOC_Os02g41860	15.073 ± 0.646	7.048 ± 0.869	0.001	2.14
LOC_Os02g42150	3.265 ± 0.619	12.209 ± 1.478	0.006	0.27
LOC_Os02g42160	5.017 ± 0.326	17.690 ± 1.620	0.006	0.28

LOC_Os02g43500	43.378 ± 6.097	98.683 ± 6.598	0.001	0.44
LOC_Os02g43540	26.357 ± 6.223	9.093 ± 2.026	0.048	2.90
LOC_Os02g43700	20.682 ± 1.227	8.213 ± 0.287	0.003	2.52
LOC_Os02g44940	20.860 ± 0.111	3.951 ± 0.725	0.001	5.28
LOC_Os02g45225	29.081 ± 3.078	105.597 ± 7.055	0.001	0.28
LOC_Os02g45710	84.283 ± 11.463	21.392 ± 1.806	0.014	3.94
LOC_Os02g45780	27.269 ± 0.468	1.068 ± 0.513	0.000	25.53
LOC_Os02g46220	14.789 ± 0.814	30.887 ± 2.276	0.005	0.48
LOC_Os02g47470	21.636 ± 3.811	3.636 ± 0.247	0.021	5.95
LOC_Os02g48560	24.733 ± 2.835	9.460 ± 0.735	0.013	2.61
LOC_Os02g48710	258.632 ± 56.886	586.603 ± 82.271	0.013	0.44
LOC_Os02g48850	34.331 ± 5.122	0.247 ± 0.349	0.011	139.10
LOC_Os02g48900	18.054 ± 0.581	7.891 ± 1.116	0.001	2.29
LOC_Os02g49760	11.528 ± 1.412	28.080 ± 1.999	0.001	0.41
LOC_Os02g50240	21.219 ± 3.343	49.232 ± 0.876	0.005	0.43
LOC_Os02g50460	4.759 ± 0.499	15.254 ± 1.743	0.009	0.31
LOC_Os02g50470	15.583 ± 6.130	51.382 ± 0.920	0.013	0.30
LOC_Os02g50540	12.606 ± 3.182	26.800 ± 1.854	0.010	0.47
LOC_Os02g50570	19.341 ± 2.283	8.555 ± 1.317	0.009	2.26
LOC_Os02g50600	41.424 ± 0.452	6.556 ± 1.692	0.001	6.32
LOC_Os02g50620	11.098 ± 1.248	24.801 ± 1.220	0.000	0.45
LOC_Os02g51100	11.297 ± 0.922	25.594 ± 3.423	0.021	0.44
LOC_Os02g51350	22.420 ± 0.912	5.344 ± 0.433	0.000	4.20
LOC_Os02g51480	10.354 ± 1.263	21.649 ± 1.908	0.004	0.48
LOC_Os02g52010	34.708 ± 2.810	5.092 ± 0.591	0.003	6.82
LOC_Os02g52170	21.471 ± 3.901	6.435 ± 1.505	0.021	3.34
LOC_Os02g52210	13.271 ± 0.968	0.728 ± 0.215	0.002	18.22
LOC_Os02g52860	10.574 ± 0.426	25.287 ± 3.426	0.025	0.42
LOC_Os02g53180	7.457 ± 0.462	19.776 ± 1.975	0.010	0.38
LOC_Os02g53410	17.819 ± 2.099	6.891 ± 0.335	0.016	2.59
LOC_Os02g54470	93.163 ± 15.304	38.226 ± 3.404	0.032	2.44
LOC_Os02g54780	14.908 ± 1.205	5.367 ± 0.281	0.006	2.78
LOC_Os02g54890	28.190 ± 2.919	3.885 ± 0.922	0.004	7.26
LOC_Os02g57280	150.157 ± 5.629	333.589 ± 30.298	0.011	0.45
LOC_Os02g57290	40.554 ± 1.321	87.074 ± 9.064	0.017	0.47
LOC_Os02g57670	22.572 ± 1.296	64.986 ± 9.735	0.024	0.35
LOC_Os02g57720	41.015 ± 3.429	17.001 ± 1.047	0.006	2.41
LOC_Os02g57924	2.879 ± 0.500	10.460 ± 0.781	0.001	0.28
LOC_Os02g58139	6.609 ± 0.295	14.374 ± 0.266	0.000	0.46
LOC_Os03g01300	70.732 ± 8.605	4.460 ± 0.858	0.008	15.86
LOC_Os03g01320	40.509 ± 3.342	9.885 ± 0.771	0.004	4.10
LOC_Os03g01350	16.471 ± 1.073	33.591 ± 1.429	0.000	0.49
LOC_Os03g01740	60.379 ± 7.608	15.706 ± 2.450	0.009	3.84
LOC_Os03g01830	34.412 ± 0.673	74.683 ± 1.910	0.000	0.46
LOC_Os03g02020	16.414 ± 0.553	51.383 ± 1.696	0.000	0.32

LOC_Os03g02070	14.070 ± 2.305	3.312 ± 0.632	0.017	4.25
LOC_Os03g02470	0.054 ± 0.076	48.457 ± 2.313	0.001	0.00
LOC_Os03g02550	6.825 ± 0.205	15.378 ± 1.416	0.012	0.44
LOC_Os03g03510	13.625 ± 1.087	4.647 ± 0.657	0.001	2.93
LOC_Os03g03724	4.572 ± 0.669	22.737 ± 1.141	0.000	0.20
LOC_Os03g04370	31.815 ± 3.885	14.753 ± 3.665	0.011	2.16
LOC_Os03g04470	25.176 ± 2.107	58.104 ± 2.294	0.000	0.43
LOC_Os03g05470	8.429 ± 0.652	22.988 ± 1.163	0.000	0.37
LOC_Os03g05750	54.756 ± 3.960	5.222 ± 0.989	0.002	10.49
LOC_Os03g06970	55.775 ± 5.437	2.314 ± 0.442	0.005	24.10
LOC_Os03g07400	18.362 ± 1.234	3.934 ± 0.611	0.001	4.67
LOC_Os03g07870	42.833 ± 3.341	20.355 ± 1.960	0.003	2.10
LOC_Os03g07994	8.476 ± 1.392	23.846 ± 1.200	0.000	0.36
LOC_Os03g08080	10.846 ± 0.370	22.660 ± 2.358	0.017	0.48
LOC_Os03g08280	7.873 ± 0.300	16.516 ± 0.846	0.002	0.48
LOC_Os03g08330	106.750 ± 15.056	4.251 ± 0.811	0.010	25.11
LOC_Os03g08500	170.069 ± 8.295	64.924 ± 13.471	0.002	2.62
LOC_Os03g08520	11.447 ± 2.685	1.640 ± 0.535	0.031	6.98
LOC_Os03g08940	10.340 ± 1.427	2.897 ± 0.914	0.006	3.57
LOC_Os03g08970	57.365 ± 8.436	23.839 ± 3.693	0.018	2.41
LOC_Os03g10100	7.049 ± 0.365	19.367 ± 0.176	0.000	0.36
LOC_Os03g10950	13.541 ± 1.004	5.628 ± 0.278	0.005	2.41
LOC_Os03g11670	8.789 ± 1.055	18.480 ± 0.796	0.001	0.48
LOC_Os03g11780	67.203 ± 5.681	147.661 ± 6.647	0.000	0.46
LOC_Os03g12990	17.881 ± 3.047	41.518 ± 2.937	0.001	0.43
LOC_Os03g13450	21.499 ± 0.827	9.566 ± 1.271	0.001	2.25
LOC_Os03g13840	22.297 ± 1.265	6.072 ± 0.262	0.002	3.67
LOC_Os03g14205	5.704 ± 0.755	27.972 ± 6.238	0.035	0.20
LOC_Os03g14370	15.435 ± 1.653	5.984 ± 0.599	0.008	2.58
LOC_Os03g15110	2.699 ± 0.180	13.202 ± 2.116	0.019	0.20
LOC_Os03g15600	4.857 ± 0.277	10.427 ± 0.800	0.006	0.47
LOC_Os03g15780	15.303 ± 1.094	46.004 ± 2.270	0.001	0.33
LOC_Os03g16030	103.543 ± 15.235	20.167 ± 4.405	0.011	5.13
LOC_Os03g16780	6.463 ± 0.282	16.207 ± 0.759	0.001	0.40
LOC_Os03g17174	5.954 ± 0.845	15.930 ± 1.066	0.001	0.37
LOC_Os03g17200	7.532 ± 0.389	20.480 ± 0.373	0.000	0.37
LOC_Os03g17470	93.664 ± 3.030	42.107 ± 4.931	0.001	2.22
LOC_Os03g17700	24.817 ± 2.848	9.675 ± 1.193	0.009	2.57
LOC_Os03g18070	12.139 ± 0.795	2.436 ± 0.313	0.001	4.98
LOC_Os03g18150	109.987 ± 11.121	32.945 ± 3.059	0.007	3.34
LOC_Os03g18180	5.263 ± 0.757	10.749 ± 2.003	0.047	0.49
LOC_Os03g18200	93.033 ± 6.698	32.900 ± 0.565	0.006	2.83
LOC_Os03g18264	9.507 ± 0.563	23.635 ± 1.221	0.001	0.40
LOC_Os03g18740	18.482 ± 1.794	4.850 ± 0.866	0.003	3.81
LOC_Os03g18779	0.033 ± 0.046	37.067 ± 0.784	0.000	0.00

LOC_Os03g18820	11.667 ± 0.795	3.584 ± 0.521	0.001	3.26
LOC_Os03g18910	24.304 ± 2.311	1.684 ± 0.385	0.004	14.43
LOC_Os03g19040	9.714 ± 1.057	25.927 ± 2.911	0.009	0.37
LOC_Os03g19070	19.785 ± 3.228	1.837 ± 0.509	0.014	10.77
LOC_Os03g19400	16.321 ± 1.512	7.157 ± 1.876	0.007	2.28
LOC_Os03g19720	119.746 ± 20.188	45.706 ± 10.888	0.019	2.62
LOC_Os03g20090	12.800 ± 2.422	2.169 ± 0.310	0.023	5.90
LOC_Os03g20330	35.021 ± 4.836	174.628 ± 10.756	0.001	0.20
LOC_Os03g20600	6.357 ± 2.561	16.487 ± 0.986	0.020	0.39
LOC_Os03g20630	5.326 ± 0.241	11.675 ± 1.606	0.028	0.46
LOC_Os03g20640	37.928 ± 4.430	79.323 ± 2.023	0.002	0.48
LOC_Os03g20780	57.716 ± 7.108	115.866 ± 3.773	0.002	0.50
LOC_Os03g21230	18.654 ± 1.579	46.922 ± 2.918	0.001	0.40
LOC_Os03g21490	11.322 ± 0.061	24.472 ± 3.163	0.028	0.46
LOC_Os03g21664	108.572 ± 9.740	250.192 ± 9.267	0.000	0.43
LOC_Os03g22430	8.545 ± 0.107	22.581 ± 0.172	0.000	0.38
LOC_Os03g24640	15270.343± 2977.355	57.375 ± 20.606	0.019	266.15
LOC_Os03g24930	26.893 ± 1.551	113.493 ± 3.331	0.000	0.24
LOC_Os03g25500	5.573 ± 0.134	15.862 ± 1.133	0.005	0.35
LOC_Os03g27080	31.736 ± 2.272	76.193 ± 2.738	0.000	0.42
LOC_Os03g27120	46.582 ± 1.776	134.025 ± 6.019	0.001	0.35
LOC_Os03g27830	3.798 ± 0.522	10.138 ± 1.113	0.006	0.37
LOC_Os03g29184	0.109 ± 0.077	32.324 ± 1.314	0.001	0.00
LOC_Os03g29240	32.144 ± 1.202	6.706 ± 0.212	0.001	4.79
LOC_Os03g29770	110.109 ± 9.935	229.469 ± 14.995	0.001	0.48
LOC_Os03g29850	8.839 ± 2.111	36.806 ± 6.511	0.018	0.24
LOC_Os03g31510	150.021 ± 5.855	58.036 ± 1.776	0.001	2.58
LOC_Os03g32160	5.646 ± 0.538	12.028 ± 1.825	0.030	0.47
LOC_Os03g36080	27.260 ± 1.874	11.585 ± 0.764	0.003	2.35
LOC_Os03g38120	5.688 ± 0.963	14.127 ± 1.000	0.001	0.40
LOC_Os03g38150	1.671 ± 1.435	13.245 ± 2.965	0.017	0.13
LOC_Os03g38350	17.258 ± 1.571	41.589 ± 4.052	0.007	0.41
LOC_Os03g40550	5.438 ± 0.976	12.358 ± 0.170	0.008	0.44
LOC_Os03g41390	10.404 ± 0.312	0.598 ± 0.376	0.000	17.41
LOC_Os03g42130	66.567 ± 0.727	10.407 ± 2.784	0.001	6.40
LOC_Os03g42530	18.179 ± 2.194	6.797 ± 1.927	0.006	2.67
LOC_Os03g42600	4.907 ± 1.016	18.205 ± 2.479	0.009	0.27
LOC_Os03g43100	0.000 ± 0.000	15.011 ± 1.856	0.008	0.00
LOC_Os03g43420	9.680 ± 0.291	20.319 ± 1.436	0.007	0.48
LOC_Os03g43810	4.546 ± 0.963	10.285 ± 1.994	0.037	0.44
LOC_Os03g45280	628.442 ± 41.470	1289.663 ± 177.163	0.029	0.49
LOC_Os03g45370	13.284 ± 0.977	3.766 ± 0.916	0.001	3.53
LOC_Os03g45400	20.226 ± 1.103	61.059 ± 4.917	0.005	0.33
LOC_Os03g46010	14.988 ± 1.634	61.118 ± 6.443	0.007	0.25
LOC_Os03g46070	44.491 ± 1.271	235.550 ± 10.912	0.001	0.19

LOC_Os03g46200	9.037 ± 1.349	24.461 ± 3.007	0.009	0.37
LOC_Os03g46230	3.071 ± 0.066	21.285 ± 1.054	0.002	0.14
LOC_Os03g47280	12.847 ± 0.690	2.824 ± 1.431	0.003	4.55
LOC_Os03g47530	17.971 ± 1.935	0.150 ± 0.112	0.006	119.48
LOC_Os03g48060	12.668 ± 0.455	3.398 ± 1.075	0.002	3.73
LOC_Os03g48920	20.067 ± 0.570	40.158 ± 2.514	0.006	0.50
LOC_Os03g49440	15.046 ± 1.014	5.333 ± 0.753	0.001	2.82
LOC_Os03g49720	6.888 ± 0.874	14.224 ± 1.646	0.011	0.48
LOC_Os03g50210	17.278 ± 3.043	2.825 ± 1.114	0.013	6.12
LOC_Os03g50850	6.131 ± 0.176	15.700 ± 1.971	0.020	0.39
LOC_Os03g50960	95.010 ± 1.511	24.575 ± 4.733	0.001	3.87
LOC_Os03g51390	16.856 ± 2.083	5.187 ± 0.391	0.013	3.25
LOC_Os03g51950	18.559 ± 0.186	37.625 ± 2.020	0.005	0.49
LOC_Os03g52410	32.412 ± 7.184	2.716 ± 1.015	0.026	11.94
LOC_Os03g52460	7.205 ± 0.310	15.309 ± 0.978	0.004	0.47
LOC_Os03g52630	11.169 ± 0.043	3.538 ± 0.579	0.003	3.16
LOC_Os03g52640	10.620 ± 0.631	25.387 ± 0.894	0.000	0.42
LOC_Os03g52720	13.464 ± 1.927	4.793 ± 0.699	0.015	2.81
LOC_Os03g52730	11.164 ± 0.395	22.917 ± 1.398	0.004	0.49
LOC_Os03g53010	4.991 ± 1.361	11.557 ± 1.739	0.015	0.43
LOC_Os03g53020	64.791 ± 2.060	14.904 ± 1.325	0.000	4.35
LOC_Os03g53070	127.780 ± 9.662	44.880 ± 0.948	0.006	2.85
LOC_Os03g53230	98.466 ± 5.337	26.328 ± 3.260	0.000	3.74
LOC_Os03g53670	31.407 ± 1.364	63.223 ± 4.322	0.005	0.50
LOC_Os03g53800	12.360 ± 2.275	1.864 ± 0.262	0.022	6.63
LOC_Os03g54900	12.666 ± 0.713	26.834 ± 0.986	0.000	0.47
LOC_Os03g55430	16.961 ± 3.496	3.088 ± 0.988	0.023	5.49
LOC_Os03g55540	271.930 ± 22.237	61.543 ± 9.615	0.002	4.42
LOC_Os03g55590	35.447 ± 4.400	16.286 ± 1.703	0.016	2.18
LOC_Os03g55670	63.808 ± 2.810	155.939 ± 16.400	0.013	0.41
LOC_Os03g55874	10.351 ± 0.750	3.470 ± 0.777	0.001	2.98
LOC_Os03g56000	29.496 ± 0.570	13.330 ± 1.226	0.001	2.21
LOC_Os03g56160	37.626 ± 7.288	83.996 ± 2.168	0.008	0.45
LOC_Os03g56840	9.088 ± 1.009	20.615 ± 1.379	0.001	0.44
LOC_Os03g57220	199.540 ± 30.314	467.105 ± 10.704	0.003	0.43
LOC_Os03g57545	16.877 ± 4.045	39.408 ± 3.602	0.004	0.43
LOC_Os03g57790	17.985 ± 0.870	50.567 ± 6.791	0.020	0.36
LOC_Os03g58300	14.380 ± 1.659	3.573 ± 0.335	0.009	4.02
LOC_Os03g59180	31.517 ± 4.364	85.137 ± 4.568	0.000	0.37
LOC_Os03g59740	40.302 ± 2.307	84.370 ± 3.759	0.000	0.48
LOC_Os03g59770	25.383 ± 5.098	5.805 ± 0.455	0.031	4.37
LOC_Os03g59774	4.943 ± 0.429	11.313 ± 0.646	0.001	0.44
LOC_Os03g60080	293.077 ± 43.510	88.794 ± 5.751	0.020	3.30
LOC_Os03g60090	9.611 ± 0.054	21.345 ± 0.134	0.000	0.45
LOC_Os03g60560	26.676 ± 4.171	3.172 ± 0.956	0.012	8.41

LOC_Os03g60650	18.736 ± 1.052	7.838 ± 1.201	0.001	2.39
LOC_Os03g60670	27.961 ± 4.417	3.201 ± 0.553	0.014	8.73
LOC_Os03g60780	72.571 ± 12.705	30.455 ± 1.194	0.042	2.38
LOC_Os03g60820	16.926 ± 0.616	7.546 ± 0.913	0.001	2.24
LOC_Os03g61030	5.994 ± 0.670	15.153 ± 0.593	0.000	0.40
LOC_Os03g61150	40.029 ± 5.242	18.847 ± 2.272	0.017	2.12
LOC_Os03g61260	27.706 ± 4.804	67.762 ± 4.817	0.001	0.41
LOC_Os03g61900	2.231 ± 1.185	27.982 ± 3.686	0.006	0.08
LOC_Os03g62180	15.616 ± 2.613	33.473 ± 1.627	0.002	0.47
LOC_Os03g62379	3.382 ± 0.535	10.094 ± 0.649	0.000	0.34
LOC_Os03g62670	10.081 ± 3.506	25.206 ± 2.357	0.010	0.40
LOC_Os03g64140	7.960 ± 1.339	17.294 ± 2.966	0.031	0.46
LOC_Os04g01330	53.820 ± 6.386	0.654 ± 0.196	0.007	82.26
LOC_Os04g01570	13.980 ± 0.918	6.512 ± 1.736	0.012	2.15
LOC_Os04g05650	35.979 ± 3.275	2.790 ± 1.220	0.002	12.89
LOC_Os04g06520	52.561 ± 5.385	11.897 ± 2.744	0.003	4.42
LOC_Os04g07110	2.036 ± 0.342	28.920 ± 3.075	0.006	0.07
LOC_Os04g08630	1.468 ± 0.271	18.981 ± 2.699	0.011	0.08
LOC_Os04g10160	1.610 ± 0.114	14.037 ± 1.044	0.003	0.11
LOC_Os04g10940	102.125 ± 1.349	242.576 ± 18.467	0.008	0.42
LOC_Os04g11450	14.186 ± 0.667	5.019 ± 0.939	0.001	2.83
LOC_Os04g12600	0.000 ± 0.000	21.488 ± 1.248	0.002	0.00
LOC_Os04g14550	31.594 ± 3.604	0.034 ± 0.033	0.006	918.00
LOC_Os04g14690	3.896 ± 0.063	12.521 ± 0.264	0.000	0.31
LOC_Os04g15580	5.279 ± 0.150	10.706 ± 0.853	0.010	0.49
LOC_Os04g15660	4.835 ± 0.127	13.631 ± 0.355	0.000	0.35
LOC_Os04g16722	12138.694 ± 2517.405	4356.567 ± 705.921	0.040	2.79
LOC_Os04g16732	19.956 ± 3.003	4.387 ± 0.752	0.014	4.55
LOC_Os04g16745	19.768 ± 1.522	5.897 ± 1.685	0.001	3.35
LOC_Os04g16748	31.527 ± 3.055	12.884 ± 2.048	0.003	2.45
LOC_Os04g16760	11.144 ± 0.515	4.326 ± 0.455	0.000	2.58
LOC_Os04g16770	512.476 ± 28.268	107.057 ± 21.240	0.000	4.79
LOC_Os04g16772	43.333 ± 7.615	15.784 ± 3.838	0.020	2.75
LOC_Os04g16775	10.278 ± 2.243	2.403 ± 0.763	0.028	4.28
LOC_Os04g16816	24.776 ± 2.667	6.740 ± 0.535	0.009	3.68
LOC_Os04g16818	41.166 ± 2.089	13.804 ± 3.501	0.002	2.98
LOC_Os04g16832	11.405 ± 1.623	3.076 ± 0.637	0.010	3.71
LOC_Os04g16872	55.394 ± 7.734	23.091 ± 1.656	0.023	2.40
LOC_Os04g16874	171.702 ± 27.157	65.652 ± 2.628	0.030	2.62
LOC_Os04g17100	19.547 ± 1.169	54.513 ± 4.879	0.007	0.36
LOC_Os04g20164	17.942 ± 1.505	50.120 ± 0.328	0.001	0.36
LOC_Os04g21130	2.060 ± 0.422	10.628 ± 0.797	0.001	0.19
LOC_Os04g21820	3.514 ± 0.119	12.330 ± 1.243	0.009	0.28
LOC_Os04g22270	3.847 ± 0.338	11.697 ± 1.178	0.007	0.33

LOC_Os04g23550	71.369 ± 2.362	2.196 ± 0.284	0.000	32.50
LOC_Os04g23820	15.062 ± 0.794	31.559 ± 1.794	0.002	0.48
LOC_Os04g24220	13.117 ± 1.315	50.082 ± 4.687	0.005	0.26
LOC_Os04g26320	6.494 ± 0.469	15.767 ± 1.687	0.011	0.41
LOC_Os04g26910	9.271 ± 0.784	39.964 ± 3.473	0.005	0.23
LOC_Os04g26920	12.676 ± 1.489	233.588 ± 3.180	0.000	0.05
LOC_Os04g27860	10.116 ± 2.306	20.513 ± 3.350	0.028	0.49
LOC_Os04g29550	46.211 ± 7.900	107.068 ± 14.553	0.013	0.43
LOC_Os04g29770	0.000 ± 0.000	21.611 ± 1.088	0.001	0.00
LOC_Os04g29810	0.000 ± 0.000	24.789 ± 3.412	0.009	0.00
LOC_Os04g30030	0.407 ± 0.060	21.082 ± 0.530	0.000	0.02
LOC_Os04g30240	6.999 ± 0.101	15.081 ± 2.020	0.030	0.46
LOC_Os04g30250	6.815 ± 0.446	14.708 ± 1.172	0.005	0.46
LOC_Os04g30420	70.172 ± 15.319	11.250 ± 0.841	0.032	6.24
LOC_Os04g31010	6.025 ± 0.507	13.209 ± 1.163	0.006	0.46
LOC_Os04g31030	24.947 ± 0.479	62.226 ± 1.775	0.001	0.40
LOC_Os04g31040	55.047 ± 5.155	26.218 ± 1.898	0.009	2.10
LOC_Os04g31690	4.848 ± 0.707	13.568 ± 1.560	0.007	0.36
LOC_Os04g31760	18.506 ± 1.504	114.747 ± 8.152	0.003	0.16
LOC_Os04g31820	11.544 ± 0.984	4.475 ± 1.451	0.007	2.58
LOC_Os04g32850	26.491 ± 3.238	4.845 ± 0.740	0.008	5.47
LOC_Os04g34290	5.710 ± 0.207	14.781 ± 1.260	0.008	0.39
LOC_Os04g34320	2.301 ± 0.105	10.484 ± 1.423	0.014	0.22
LOC_Os04g35190	4.260 ± 0.243	10.150 ± 0.798	0.005	0.42
LOC_Os04g35864	2.321 ± 0.190	20.120 ± 1.839	0.005	0.12
LOC_Os04g36570	8.714 ± 1.389	34.456 ± 2.359	0.001	0.25
LOC_Os04g36790	7.776 ± 1.960	17.894 ± 3.748	0.043	0.43
LOC_Os04g37619	35.693 ± 2.549	71.490 ± 4.049	0.001	0.50
LOC_Os04g37710	23.269 ± 0.820	8.933 ± 0.729	0.000	2.60
LOC_Os04g37740	10.016 ± 0.884	21.417 ± 2.301	0.011	0.47
LOC_Os04g38390	107.054 ± 15.493	51.018 ± 0.442	0.036	2.10
LOC_Os04g38480	20.646 ± 3.209	52.416 ± 2.684	0.001	0.39
LOC_Os04g38600	431.765 ± 18.314	191.361 ± 1.112	0.003	2.26
LOC_Os04g39140	7.840 ± 0.471	16.207 ± 1.640	0.013	0.48
LOC_Os04g39700	22.868 ± 2.837	49.294 ± 3.161	0.001	0.46
LOC_Os04g39950	5.479 ± 0.505	11.370 ± 1.191	0.010	0.48
LOC_Os04g39980	11.374 ± 1.974	5.639 ± 1.370	0.033	2.02
LOC_Os04g40420	5.916 ± 0.255	13.433 ± 1.718	0.023	0.44
LOC_Os04g41960	168.886 ± 18.930	28.622 ± 3.926	0.007	5.90
LOC_Os04g42250	8.379 ± 0.855	17.707 ± 0.996	0.001	0.47
LOC_Os04g42520	4.033 ± 0.366	11.244 ± 0.507	0.000	0.36
LOC_Os04g43680	16.408 ± 0.970	4.218 ± 1.047	0.000	3.89
LOC_Os04g43710	5.819 ± 0.483	16.099 ± 1.790	0.011	0.36
LOC_Os04g43770	5.077 ± 0.596	12.830 ± 1.417	0.008	0.40
LOC_Os04g43800	41.573 ± 1.063	7.277 ± 0.041	0.000	5.71

LOC_Os04g44430	5.866 ± 0.205	12.044 ± 0.786	0.005	0.49
LOC_Os04g44924	15.848 ± 3.093	37.679 ± 5.831	0.018	0.42
LOC_Os04g44950	7.582 ± 1.858	15.548 ± 2.200	0.018	0.49
LOC_Os04g45460	10.075 ± 1.274	4.629 ± 0.771	0.011	2.18
LOC_Os04g45710	4.140 ± 0.610	10.668 ± 1.728	0.023	0.39
LOC_Os04g45940	59.412 ± 8.891	28.797 ± 3.643	0.026	2.06
LOC_Os04g46010	23.693 ± 3.838	50.135 ± 1.888	0.004	0.47
LOC_Os04g46440	13.942 ± 1.851	3.479 ± 0.893	0.006	4.01
LOC_Os04g47360	41.381 ± 4.313	238.666 ± 5.714	0.000	0.17
LOC_Os04g48030	40.759 ± 8.041	19.810 ± 4.383	0.046	2.06
LOC_Os04g48350	19.079 ± 2.598	0.750 ± 0.151	0.010	25.43
LOC_Os04g49510	26.319 ± 0.989	61.573 ± 2.620	0.001	0.43
LOC_Os04g49660	3.782 ± 0.118	12.817 ± 0.592	0.002	0.30
LOC_Os04g49690	18.836 ± 0.375	6.783 ± 0.788	0.000	2.78
LOC_Os04g50970	24.975 ± 5.063	61.277 ± 1.669	0.005	0.41
LOC_Os04g51280	28.777 ± 2.074	61.234 ± 1.617	0.000	0.47
LOC_Os04g51792	17.401 ± 0.749	37.004 ± 3.192	0.010	0.47
LOC_Os04g52090	101.857 ± 9.393	11.384 ± 3.227	0.003	8.95
LOC_Os04g52260	240.114 ± 13.102	42.715 ± 4.617	0.001	5.62
LOC_Os04g52840	20.677 ± 2.998	9.334 ± 0.728	0.028	2.22
LOC_Os04g53860	3.052 ± 0.207	10.847 ± 2.379	0.043	0.28
LOC_Os04g54630	65.881 ± 15.320	245.558 ± 19.510	0.001	0.27
LOC_Os04g55050	20.190 ± 1.333	42.474 ± 1.993	0.000	0.48
LOC_Os04g55710	33.689 ± 4.882	87.008 ± 6.278	0.001	0.39
LOC_Os04g55980	15.081 ± 2.185	3.468 ± 1.346	0.006	4.35
LOC_Os04g57760	53.672 ± 2.515	24.883 ± 2.720	0.000	2.16
LOC_Os04g58055	28.049 ± 1.060	11.720 ± 1.154	0.000	2.39
LOC_Os04g58280	45.518 ± 4.407	93.956 ± 2.657	0.001	0.48
LOC_Os04g58410	9.631 ± 0.250	22.262 ± 2.618	0.020	0.43
LOC_Os04g58710	47.245 ± 7.856	20.394 ± 1.844	0.034	2.32
LOC_Os04g58810	60.640 ± 5.716	12.670 ± 1.680	0.004	4.79
LOC_Os04g58850	154.827 ± 17.358	36.163 ± 1.055	0.010	4.28
LOC_Os05g01290	229.741 ± 13.261	85.465 ± 15.690	0.001	2.69
LOC_Os05g01675	10.694 ± 1.191	4.781 ± 1.223	0.008	2.24
LOC_Os05g02070	46.305 ± 0.434	187.612 ± 18.354	0.008	0.25
LOC_Os05g02140	12.069 ± 1.792	4.362 ± 0.346	0.023	2.77
LOC_Os05g02770	17.623 ± 2.962	8.366 ± 1.912	0.027	2.11
LOC_Os05g03040	37.235 ± 7.101	95.716 ± 5.033	0.001	0.39
LOC_Os05g03150	5.708 ± 0.054	25.622 ± 4.838	0.028	0.22
LOC_Os05g03620	15.614 ± 1.310	2.582 ± 0.385	0.003	6.05
LOC_Os05g03740	14.268 ± 1.288	6.777 ± 1.323	0.005	2.11
LOC_Os05g04584	3.772 ± 0.366	11.781 ± 1.194	0.007	0.32
LOC_Os05g04700	358.813 ± 59.095	99.582 ± 7.486	0.024	3.60
LOC_Os05g04830	8.851 ± 0.319	22.905 ± 0.483	0.000	0.39
LOC_Os05g05040	44.169 ± 3.837	95.410 ± 17.705	0.049	0.46

LOC_Os05g05060	43.576 ± 4.152	94.997 ± 11.677	0.016	0.46
LOC_Os05g05680	24.523 ± 0.872	53.225 ± 2.338	0.001	0.46
LOC_Os05g05940	229.521 ± 7.915	84.103 ± 4.754	0.000	2.73
LOC_Os05g06440	18.080 ± 1.599	49.604 ± 9.496	0.039	0.36
LOC_Os05g06920	11.948 ± 2.524	2.958 ± 0.205	0.036	4.04
LOC_Os05g07632	5.099 ± 0.982	10.502 ± 0.542	0.006	0.49
LOC_Os05g08370	10.505 ± 0.539	3.624 ± 0.111	0.002	2.90
LOC_Os05g08420	159.016 ± 27.706	48.393 ± 10.987	0.019	3.29
LOC_Os05g08860	11.474 ± 0.841	5.042 ± 1.687	0.018	2.28
LOC_Os05g08900	17.446 ± 0.554	4.553 ± 0.461	0.000	3.83
LOC_Os05g08910	25.419 ± 6.405	7.794 ± 2.215	0.048	3.26
LOC_Os05g09020	11.129 ± 0.760	34.370 ± 4.638	0.017	0.32
LOC_Os05g09728	22.868 ± 1.560	56.606 ± 5.786	0.010	0.40
LOC_Os05g10310	14.127 ± 0.086	4.359 ± 0.309	0.000	3.24
LOC_Os05g10414	71.995 ± 7.857	167.116 ± 18.864	0.010	0.43
LOC_Os05g10754	11.558 ± 1.124	23.438 ± 1.763	0.003	0.49
LOC_Os05g11320	87.240 ± 11.605	360.942 ± 7.694	0.000	0.24
LOC_Os05g13904	5.364 ± 1.015	10.971 ± 0.449	0.007	0.49
LOC_Os05g13940	139.358 ± 13.309	38.072 ± 6.112	0.003	3.66
LOC_Os05g14240	48.798 ± 0.734	110.961 ± 11.070	0.015	0.44
LOC_Os05g15770	133.352 ± 10.841	8.769 ± 0.341	0.004	15.21
LOC_Os05g19910	3.157 ± 0.477	11.696 ± 0.632	0.000	0.27
LOC_Os05g21180	5.749 ± 0.263	12.812 ± 1.233	0.012	0.45
LOC_Os05g22716	18.857 ± 5.948	0.476 ± 0.404	0.048	39.62
LOC_Os05g22718	13.994 ± 3.172	2.535 ± 0.399	0.034	5.52
LOC_Os05g24650	52.609 ± 8.338	116.800 ± 19.504	0.029	0.45
LOC_Os05g24970	13.027 ± 0.912	28.990 ± 4.621	0.035	0.45
LOC_Os05g25350	6.428 ± 0.727	13.639 ± 1.865	0.021	0.47
LOC_Os05g25450	27.357 ± 1.437	55.725 ± 8.826	0.042	0.49
LOC_Os05g26990	9.430 ± 0.462	19.047 ± 0.264	0.000	0.50
LOC_Os05g27010	9.306 ± 1.473	19.875 ± 1.677	0.003	0.47
LOC_Os05g27780	157.632 ± 7.633	337.021 ± 23.366	0.005	0.47
LOC_Os05g28740	169.969 ± 4.071	364.421 ± 6.386	0.000	0.47
LOC_Os05g29990	13.155 ± 0.103	28.809 ± 1.590	0.005	0.46
LOC_Os05g30500	49.211 ± 8.465	18.273 ± 5.928	0.017	2.69
LOC_Os05g30850	8.392 ± 1.726	23.890 ± 3.365	0.010	0.35
LOC_Os05g31620	33.824 ± 1.438	4.624 ± 1.532	0.000	7.31
LOC_Os05g31744	4.935 ± 1.207	19.797 ± 1.845	0.001	0.25
LOC_Os05g31750	26.692 ± 1.903	11.566 ± 1.949	0.001	2.31
LOC_Os05g32140	27.808 ± 1.725	58.009 ± 3.920	0.003	0.48
LOC_Os05g32330	5.607 ± 0.602	11.933 ± 0.914	0.002	0.47
LOC_Os05g32680	5.213 ± 0.251	11.017 ± 1.053	0.012	0.47
LOC_Os05g32860	15.757 ± 2.722	1.050 ± 0.830	0.011	15.01
LOC_Os05g33090	172.024 ± 16.902	83.043 ± 8.037	0.008	2.07
LOC_Os05g33210	6.859 ± 1.393	16.976 ± 0.931	0.002	0.40

LOC_Os05g33900	19.574 ± 2.128	43.653 ± 2.304	0.000	0.45
LOC_Os05g34500	8.184 ± 1.180	16.867 ± 1.832	0.008	0.49
LOC_Os05g34830	15.105 ± 0.647	5.993 ± 1.367	0.004	2.52
LOC_Os05g34980	2.226 ± 0.138	17.718 ± 2.251	0.010	0.13
LOC_Os05g35140	5.434 ± 0.265	12.392 ± 1.517	0.020	0.44
LOC_Os05g35500	24.021 ± 3.983	8.556 ± 2.357	0.015	2.81
LOC_Os05g35980	4.515 ± 0.368	11.599 ± 1.120	0.007	0.39
LOC_Os05g36994	40.150 ± 5.128	87.900 ± 5.031	0.001	0.46
LOC_Os05g37884	25.296 ± 2.878	8.207 ± 1.120	0.007	3.08
LOC_Os05g37950	5.095 ± 0.324	10.894 ± 0.502	0.000	0.47
LOC_Os05g38630	9.866 ± 1.672	27.797 ± 5.548	0.036	0.35
LOC_Os05g38880	12.430 ± 0.446	28.608 ± 0.711	0.000	0.43
LOC_Os05g39230	4.343 ± 0.274	10.374 ± 0.385	0.000	0.42
LOC_Os05g39930	27.130 ± 4.450	2.516 ± 0.145	0.016	10.78
LOC_Os05g40020	10.602 ± 1.569	26.609 ± 4.179	0.022	0.40
LOC_Os05g40270	18.938 ± 1.603	41.455 ± 5.082	0.017	0.46
LOC_Os05g41460	66.654 ± 7.207	17.723 ± 3.558	0.004	3.76
LOC_Os05g41490	11.768 ± 2.668	3.850 ± 0.747	0.044	3.06
LOC_Os05g41590	18.759 ± 0.340	3.421 ± 0.520	0.000	5.48
LOC_Os05g41780	375.156 ± 39.887	59.612 ± 4.436	0.007	6.29
LOC_Os05g42100	22.299 ± 2.989	47.803 ± 7.856	0.032	0.47
LOC_Os05g43040	48.457 ± 0.403	18.025 ± 1.517	0.001	2.69
LOC_Os05g43170	59.635 ± 1.489	202.360 ± 22.727	0.012	0.29
LOC_Os05g43310	23.129 ± 1.670	63.408 ± 2.357	0.000	0.36
LOC_Os05g44060	21.184 ± 4.582	0.901 ± 0.222	0.024	23.52
LOC_Os05g44340	14.482 ± 2.415	6.426 ± 1.311	0.024	2.25
LOC_Os05g44770	6.979 ± 0.699	27.435 ± 1.967	0.002	0.25
LOC_Os05g44900	66.886 ± 7.725	8.757 ± 3.303	0.003	7.64
LOC_Os05g45710	32.822 ± 3.433	14.033 ± 1.184	0.010	2.34
LOC_Os05g46340	51.488 ± 5.548	15.097 ± 0.652	0.011	3.41
LOC_Os05g46760	31.750 ± 3.073	6.657 ± 2.015	0.001	4.77
LOC_Os05g46830	24.249 ± 2.110	10.146 ± 3.168	0.009	2.39
LOC_Os05g47650	4.351 ± 0.065	19.795 ± 2.507	0.013	0.22
LOC_Os05g47700	39.936 ± 4.583	3.934 ± 0.410	0.008	10.15
LOC_Os05g47960	38.684 ± 12.211	0.638 ± 0.182	0.048	60.61
LOC_Os05g48200	18.998 ± 1.472	41.518 ± 1.880	0.000	0.46
LOC_Os05g48360	8.290 ± 0.988	18.706 ± 0.275	0.003	0.44
LOC_Os05g48900	19.493 ± 1.990	1.297 ± 0.347	0.005	15.03
LOC_Os05g48930	38.125 ± 2.387	12.126 ± 0.910	0.002	3.14
LOC_Os05g49050	4.109 ± 0.500	10.133 ± 1.160	0.009	0.41
LOC_Os05g49140	25.548 ± 1.529	11.039 ± 2.111	0.002	2.31
LOC_Os05g49940	3.698 ± 0.760	14.177 ± 1.935	0.009	0.26
LOC_Os05g50340	9.523 ± 2.388	53.204 ± 5.037	0.002	0.18
LOC_Os05g50624	13.023 ± 0.985	29.450 ± 2.160	0.003	0.44
LOC_Os05g50980	5.520 ± 0.111	15.287 ± 0.804	0.003	0.36

LOC_Os05g51670	23.773 ± 1.519	11.817 ± 1.854	0.002	2.01
LOC_Os06g02370	14.107 ± 1.521	31.078 ± 3.079	0.007	0.45
LOC_Os06g02470	20.869 ± 1.620	41.967 ± 4.635	0.015	0.50
LOC_Os06g02500	10.749 ± 0.468	23.811 ± 0.737	0.000	0.45
LOC_Os06g03930	14.854 ± 0.883	5.836 ± 0.177	0.004	2.55
LOC_Os06g04230	210.491 ± 16.096	17.737 ± 1.381	0.003	11.87
LOC_Os06g04240	137.237 ± 4.764	15.312 ± 2.533	0.000	8.96
LOC_Os06g05050	3.025 ± 0.035	13.074 ± 1.477	0.011	0.23
LOC_Os06g05110	10.398 ± 1.643	21.795 ± 0.748	0.004	0.48
LOC_Os06g05390	9.630 ± 1.134	21.065 ± 1.002	0.000	0.46
LOC_Os06g05450	7.276 ± 0.721	18.393 ± 0.647	0.000	0.40
LOC_Os06g05480	70.673 ± 1.957	18.253 ± 0.796	0.000	3.87
LOC_Os06g05520	9.152 ± 0.476	29.885 ± 2.583	0.006	0.31
LOC_Os06g06470	10.286 ± 1.710	5.091 ± 0.347	0.045	2.02
LOC_Os06g07941	5.724 ± 0.454	17.130 ± 0.511	0.000	0.33
LOC_Os06g08041	10.296 ± 0.216	20.768 ± 0.660	0.001	0.50
LOC_Os06g08320	94.458 ± 14.472	211.395 ± 23.034	0.006	0.45
LOC_Os06g08460	5.166 ± 1.004	11.884 ± 1.157	0.004	0.43
LOC_Os06g09560	7.311 ± 0.186	20.921 ± 3.121	0.025	0.35
LOC_Os06g09688	10.955 ± 1.048	24.656 ± 0.452	0.001	0.44
LOC_Os06g09880	11.610 ± 0.103	25.343 ± 0.717	0.001	0.46
LOC_Os06g10840	7.874 ± 0.405	30.340 ± 1.105	0.000	0.26
LOC_Os06g11150	5.758 ± 1.141	14.005 ± 1.292	0.003	0.41
LOC_Os06g11290	20.031 ± 1.141	2.074 ± 0.297	0.001	9.66
LOC_Os06g11760	47.776 ± 8.578	4.522 ± 0.968	0.018	10.57
LOC_Os06g11800	5.273 ± 0.338	12.369 ± 1.121	0.008	0.43
LOC_Os06g13880	1.887 ± 0.248	11.773 ± 1.713	0.013	0.16
LOC_Os06g14240	21.556 ± 5.201	293.748 ± 30.272	0.005	0.07
LOC_Os06g14324	4.697 ± 0.635	22.427 ± 1.279	0.000	0.21
LOC_Os06g14370	11.726 ± 0.525	1.054 ± 0.419	0.000	11.13
LOC_Os06g14400	11.011 ± 1.235	3.524 ± 0.303	0.010	3.12
LOC_Os06g14420	85.241 ± 3.544	16.820 ± 0.712	0.001	5.07
LOC_Os06g15330	23.628 ± 3.577	47.880 ± 1.288	0.006	0.49
LOC_Os06g15730	1.146 ± 0.159	16.728 ± 1.707	0.006	0.07
LOC_Os06g16050	0.013 ± 0.019	35.497 ± 5.140	0.010	0.00
LOC_Os06g19370	14.264 ± 0.878	28.679 ± 2.369	0.007	0.50
LOC_Os06g20820	5.499 ± 1.814	11.543 ± 1.450	0.023	0.48
LOC_Os06g21110	26.682 ± 1.057	78.619 ± 8.201	0.011	0.34
LOC_Os06g21530	21.589 ± 6.201	48.918 ± 4.503	0.009	0.44
LOC_Os06g21820	2.189 ± 0.647	20.155 ± 2.500	0.007	0.11
LOC_Os06g22440	3.779 ± 0.334	14.149 ± 1.668	0.010	0.27
LOC_Os06g22690	22.766 ± 2.367	51.212 ± 8.191	0.031	0.44
LOC_Os06g23775	5.513 ± 1.437	21.209 ± 1.898	0.001	0.26
LOC_Os06g25010	18.219 ± 1.060	2.958 ± 1.287	0.000	6.16
LOC_Os06g25605	4.434 ± 0.432	12.302 ± 1.031	0.003	0.36

LOC_Os06g28050	27.235 ± 2.586	57.280 ± 1.187	0.001	0.48
LOC_Os06g28194	10.104 ± 1.486	27.179 ± 0.589	0.001	0.37
LOC_Os06g29730	11.249 ± 1.142	29.183 ± 3.052	0.008	0.39
LOC_Os06g32650	0.000 ± 0.000	11.257 ± 2.024	0.016	0.00
LOC_Os06g32910	0.195 ± 0.142	11.398 ± 3.622	0.048	0.02
LOC_Os06g33200	32.607 ± 1.961	88.219 ± 1.453	0.000	0.37
LOC_Os06g33330	4.129 ± 0.283	10.135 ± 1.180	0.015	0.41
LOC_Os06g34400	4.841 ± 0.415	13.340 ± 1.887	0.020	0.36
LOC_Os06g34450	5.472 ± 0.515	13.709 ± 0.719	0.000	0.40
LOC_Os06g35560	21.368 ± 1.230	66.012 ± 11.708	0.032	0.32
LOC_Os06g36040	3.307 ± 0.364	12.517 ± 2.018	0.020	0.26
LOC_Os06g36270	10.900 ± 0.158	22.091 ± 0.378	0.000	0.49
LOC_Os06g37010	7.468 ± 0.998	29.861 ± 5.160	0.022	0.25
LOC_Os06g37150	13.426 ± 0.944	4.677 ± 0.818	0.001	2.87
LOC_Os06g38210	340.684 ± 12.758	691.843 ± 40.514	0.004	0.49
LOC_Os06g38340	29.648 ± 1.332	85.091 ± 4.337	0.001	0.35
LOC_Os06g38450	166.134 ± 6.545	480.157 ± 7.802	0.000	0.35
LOC_Os06g38580	3.804 ± 1.843	13.411 ± 2.255	0.010	0.28
LOC_Os06g38660	7.385 ± 1.727	24.713 ± 2.991	0.005	0.30
LOC_Os06g38764	0.000 ± 0.000	28.000 ± 1.506	0.001	0.00
LOC_Os06g38830	4.217 ± 0.718	12.873 ± 0.857	0.000	0.33
LOC_Os06g39120	72.450 ± 2.836	34.994 ± 3.334	0.000	2.07
LOC_Os06g39240	339.180 ± 33.868	81.285 ± 4.863	0.008	4.17
LOC_Os06g39690	12.674 ± 0.602	41.769 ± 0.853	0.000	0.30
LOC_Os06g39740	12.916 ± 1.644	2.910 ± 0.172	0.013	4.44
LOC_Os06g39744	11.598 ± 2.519	4.676 ± 1.830	0.039	2.48
LOC_Os06g39750	8.329 ± 0.579	22.986 ± 1.099	0.000	0.36
LOC_Os06g40010	1.397 ± 0.537	10.472 ± 2.065	0.020	0.13
LOC_Os06g40640	100.815 ± 13.352	209.914 ± 30.292	0.023	0.48
LOC_Os06g40940	84.409 ± 1.127	271.771 ± 17.285	0.004	0.31
LOC_Os06g41120	0.000 ± 0.000	155.152 ± 10.053	0.002	0.00
LOC_Os06g41240	0.000 ± 0.000	33.400 ± 3.898	0.007	0.00
LOC_Os06g41720	10.267 ± 3.368	20.740 ± 2.479	0.028	0.50
LOC_Os06g43090	16.694 ± 0.317	4.752 ± 0.897	0.001	3.51
LOC_Os06g43800	13.691 ± 1.468	44.529 ± 1.992	0.000	0.31
LOC_Os06g43810	121.341 ± 3.791	56.182 ± 5.623	0.000	2.16
LOC_Os06g43850	25.052 ± 1.589	56.101 ± 2.190	0.000	0.45
LOC_Os06g43930	5.997 ± 0.166	12.288 ± 1.086	0.013	0.49
LOC_Os06g44170	28.590 ± 4.103	68.509 ± 7.094	0.005	0.42
LOC_Os06g45660	12.180 ± 1.406	31.406 ± 1.060	0.000	0.39
LOC_Os06g45970	14.220 ± 7.022	42.639 ± 9.776	0.033	0.33
LOC_Os06g45980	3.847 ± 0.189	10.726 ± 0.977	0.008	0.36
LOC_Os06g46436	131.093 ± 10.964	43.041 ± 9.808	0.001	3.05
LOC_Os06g46460	71.867 ± 18.790	159.792 ± 14.948	0.008	0.45
LOC_Os06g46950	557.147 ± 27.062	47.971 ± 1.803	0.001	11.61

LOC_Os06g47550	5.688 ± 0.147	11.971 ± 0.768	0.006	0.48
LOC_Os06g47640	60.535 ± 2.619	173.519 ± 9.133	0.002	0.35
LOC_Os06g48680	33.154 ± 6.246	74.480 ± 1.924	0.007	0.45
LOC_Os06g48770	11.272 ± 0.072	26.174 ± 3.231	0.023	0.43
LOC_Os06g49250	33.411 ± 3.016	68.263 ± 4.115	0.001	0.49
LOC_Os06g49780	24.093 ± 1.451	9.999 ± 1.057	0.001	2.41
LOC_Os06g50300	95.368 ± 1.597	220.596 ± 9.679	0.002	0.43
LOC_Os06g50490	8.110 ± 1.248	17.496 ± 0.546	0.003	0.46
LOC_Os06g50539	6.792 ± 0.844	17.689 ± 1.355	0.001	0.38
LOC_Os06g50580	8.559 ± 1.350	19.927 ± 2.822	0.016	0.43
LOC_Os06g50670	7.009 ± 1.331	17.816 ± 0.674	0.002	0.39
LOC_Os06g50706	6.938 ± 0.421	18.379 ± 1.733	0.008	0.38
LOC_Os06g50715	3.157 ± 0.567	11.667 ± 1.042	0.002	0.27
LOC_Os06g50742	6.921 ± 0.866	17.217 ± 1.080	0.001	0.40
LOC_Os06g50818	6.183 ± 0.819	18.486 ± 1.264	0.001	0.33
LOC_Os06g51070	31.221 ± 5.318	83.764 ± 7.554	0.002	0.37
LOC_Os07g01904	1.838 ± 0.181	21.220 ± 0.815	0.001	0.09
LOC_Os07g03025	5.667 ± 0.861	19.898 ± 0.585	0.000	0.28
LOC_Os07g03030	11.850 ± 1.631	34.074 ± 3.502	0.005	0.35
LOC_Os07g03040	9.707 ± 0.823	21.245 ± 1.647	0.003	0.46
LOC_Os07g03050	9.387 ± 0.421	21.092 ± 3.336	0.036	0.45
LOC_Os07g03180	197.030 ± 3.488	72.149 ± 19.812	0.011	2.73
LOC_Os07g03200	8.711 ± 3.455	44.724 ± 3.749	0.001	0.19
LOC_Os07g03910	2.063 ± 0.131	15.668 ± 0.703	0.001	0.13
LOC_Os07g03960	2.409 ± 0.400	23.612 ± 3.253	0.011	0.10
LOC_Os07g05480	811.942 ± 14.434	320.577 ± 33.696	0.001	2.53
LOC_Os07g05580	3.859 ± 1.120	14.312 ± 0.809	0.001	0.27
LOC_Os07g05640	20.720 ± 3.484	53.091 ± 8.106	0.018	0.39
LOC_Os07g05820	142.227 ± 39.801	286.634 ± 26.447	0.017	0.50
LOC_Os07g05840	0.309 ± 0.036	13.030 ± 0.439	0.001	0.02
LOC_Os07g06450	52.469 ± 3.918	111.396 ± 6.008	0.001	0.47
LOC_Os07g06834	0.102 ± 0.083	16.932 ± 1.334	0.003	0.01
LOC_Os07g06850	3.760 ± 0.432	12.992 ± 0.804	0.001	0.29
LOC_Os07g07040	23.782 ± 1.271	3.445 ± 0.665	0.000	6.90
LOC_Os07g07220	7.126 ± 0.622	15.753 ± 1.578	0.009	0.45
LOC_Os07g07620	6.448 ± 0.626	13.564 ± 2.245	0.038	0.48
LOC_Os07g08340	14.265 ± 2.603	30.147 ± 0.828	0.008	0.47
LOC_Os07g10830	11.382 ± 0.586	27.315 ± 1.049	0.000	0.42
LOC_Os07g10840	10.410 ± 1.461	3.870 ± 0.508	0.016	2.69
LOC_Os07g11280	4.575 ± 0.117	10.668 ± 1.253	0.020	0.43
LOC_Os07g11970	3.129 ± 0.555	10.211 ± 1.140	0.005	0.31
LOC_Os07g12340	43.132 ± 0.605	21.039 ± 1.617	0.001	2.05
LOC_Os07g12780	26.559 ± 2.557	58.821 ± 7.959	0.021	0.45
LOC_Os07g12890	37.814 ± 2.932	123.491 ± 4.706	0.000	0.31
LOC_Os07g13230	0.644 ± 0.271	13.854 ± 1.320	0.004	0.05

LOC_Os07g13530	12.921 ± 0.389	28.318 ± 1.678	0.004	0.46
LOC_Os07g14740	10.938 ± 1.228	23.934 ± 0.899	0.000	0.46
LOC_Os07g17184	31.200 ± 3.640	74.456 ± 4.818	0.001	0.42
LOC_Os07g17250	7.179 ± 0.569	15.508 ± 1.034	0.002	0.46
LOC_Os07g18240	8.092 ± 1.248	25.910 ± 1.253	0.000	0.31
LOC_Os07g18720	4.079 ± 0.133	10.375 ± 1.259	0.018	0.39
LOC_Os07g22498	130.657 ± 5.008	46.036 ± 5.114	0.000	2.84
LOC_Os07g23510	0.103 ± 0.146	19.879 ± 4.028	0.020	0.01
LOC_Os07g23640	12.536 ± 2.413	0.808 ± 0.361	0.019	15.51
LOC_Os07g24190	12.798 ± 0.455	5.581 ± 0.880	0.002	2.29
LOC_Os07g25018	21.288 ± 1.931	5.367 ± 1.082	0.002	3.97
LOC_Os07g25022	12.466 ± 2.517	3.619 ± 2.024	0.020	3.44
LOC_Os07g25024	44.226 ± 5.690	16.108 ± 4.107	0.006	2.75
LOC_Os07g26520	4.186 ± 0.685	41.984 ± 6.993	0.016	0.10
LOC_Os07g27780	8.598 ± 2.677	19.721 ± 1.349	0.014	0.44
LOC_Os07g28614	58.775 ± 10.613	149.378 ± 13.629	0.002	0.39
LOC_Os07g29224	10.691 ± 0.341	30.868 ± 5.277	0.032	0.35
LOC_Os07g30020	15.962 ± 0.565	31.986 ± 1.926	0.004	0.50
LOC_Os07g32590	5.398 ± 0.451	12.396 ± 0.572	0.000	0.44
LOC_Os07g32650	1.287 ± 0.168	11.739 ± 0.857	0.002	0.11
LOC_Os07g33480	4.501 ± 1.030	11.177 ± 2.184	0.033	0.40
LOC_Os07g33780	20.546 ± 0.663	72.776 ± 2.775	0.001	0.28
LOC_Os07g34650	8.463 ± 0.534	17.467 ± 0.703	0.000	0.48
LOC_Os07g34730	4.011 ± 0.484	11.052 ± 0.754	0.001	0.36
LOC_Os07g34830	0.587 ± 0.100	11.156 ± 0.823	0.003	0.05
LOC_Os07g35004	3.964 ± 0.272	11.537 ± 1.441	0.015	0.34
LOC_Os07g35260	7.983 ± 0.450	16.749 ± 2.272	0.028	0.48
LOC_Os07g35560	15.987 ± 0.472	46.943 ± 3.905	0.007	0.34
LOC_Os07g35680	2.002 ± 0.175	14.073 ± 0.780	0.001	0.14
LOC_Os07g35690	7.994 ± 0.502	24.512 ± 5.105	0.043	0.33
LOC_Os07g35870	4.864 ± 0.320	12.009 ± 1.803	0.027	0.41
LOC_Os07g35979	0.275 ± 0.125	48.236 ± 8.086	0.014	0.01
LOC_Os07g36170	23.896 ± 0.973	7.767 ± 0.832	0.000	3.08
LOC_Os07g36800	3.686 ± 0.622	14.907 ± 1.546	0.004	0.25
LOC_Os07g38630	218.279 ± 34.934	101.178 ± 21.067	0.023	2.16
LOC_Os07g38800	4.949 ± 0.510	15.213 ± 0.601	0.000	0.33
LOC_Os07g38810	8.596 ± 0.926	18.995 ± 2.546	0.019	0.45
LOC_Os07g39780	6.048 ± 0.109	12.919 ± 0.065	0.000	0.47
LOC_Os07g40690	3.029 ± 0.387	10.039 ± 0.978	0.004	0.30
LOC_Os07g41370	4.934 ± 0.434	10.496 ± 1.268	0.017	0.47
LOC_Os07g42040	0.000 ± 0.000	11.786 ± 2.642	0.024	0.00
LOC_Os07g42324	7.712 ± 1.183	20.784 ± 1.551	0.001	0.37
LOC_Os07g42450	8.055 ± 0.694	20.531 ± 3.831	0.040	0.39
LOC_Os07g42650	6.139 ± 0.335	14.931 ± 1.340	0.008	0.41
LOC_Os07g42730	26.258 ± 2.462	57.624 ± 4.454	0.003	0.46

LOC_Os07g43560	86.633 ± 2.792	178.694 ± 9.463	0.003	0.48
LOC_Os07g43800	22.194 ± 3.578	49.369 ± 7.942	0.025	0.45
LOC_Os07g43990	12.114 ± 0.224	1.510 ± 0.287	0.000	8.02
LOC_Os07g46410	11.125 ± 0.740	23.991 ± 2.603	0.014	0.46
LOC_Os07g46600	6.238 ± 0.381	12.765 ± 1.626	0.025	0.49
LOC_Os07g46670	69.645 ± 8.429	27.568 ± 2.110	0.015	2.53
LOC_Os07g46990	46.305 ± 7.628	109.084 ± 21.355	0.041	0.42
LOC_Os07g47400	12.264 ± 0.398	35.702 ± 1.025	0.000	0.34
LOC_Os07g48040	28.378 ± 2.119	63.365 ± 0.876	0.000	0.45
LOC_Os07g48280	36.826 ± 2.072	6.619 ± 0.695	0.001	5.56
LOC_Os07g48490	245.624 ± 31.856	1210.085 ± 188.697	0.016	0.20
LOC_Os07g48500	227.109 ± 23.183	984.521 ± 39.765	0.000	0.23
LOC_Os07g48510	20.332 ± 1.273	42.541 ± 0.063	0.002	0.48
LOC_Os07g48780	29.400 ± 1.092	80.429 ± 7.286	0.009	0.37
LOC_Os08g01220	24.358 ± 1.113	9.923 ± 1.927	0.002	2.45
LOC_Os08g01240	10.791 ± 2.013	25.575 ± 1.910	0.002	0.42
LOC_Os08g01270	8.786 ± 0.699	19.017 ± 2.038	0.012	0.46
LOC_Os08g01380	1053.140 ± 100.588	467.392 ± 37.088	0.008	2.25
LOC_Os08g01940	7.172 ± 0.666	15.566 ± 1.071	0.002	0.46
LOC_Os08g02340	43.136 ± 3.453	93.351 ± 11.274	0.017	0.46
LOC_Os08g02680	4.618 ± 0.575	10.687 ± 0.972	0.004	0.43
LOC_Os08g05480	4.952 ± 1.204	11.661 ± 2.540	0.047	0.42
LOC_Os08g05970	1.100 ± 0.770	12.898 ± 3.221	0.030	0.09
LOC_Os08g06100	20.819 ± 1.478	66.426 ± 5.505	0.005	0.31
LOC_Os08g06230	5.646 ± 0.181	12.451 ± 0.505	0.001	0.45
LOC_Os08g06550	12.966 ± 0.829	33.621 ± 3.520	0.011	0.39
LOC_Os08g06800	6.059 ± 0.949	12.753 ± 1.968	0.025	0.48
LOC_Os08g07060	10.047 ± 0.330	20.598 ± 2.797	0.032	0.49
LOC_Os08g07080	64.318 ± 9.129	1.355 ± 0.229	0.010	47.46
LOC_Os08g07660	27.744 ± 5.509	12.501 ± 3.989	0.039	2.22
LOC_Os08g07690	7.077 ± 0.688	20.153 ± 3.679	0.034	0.35
LOC_Os08g07774	10.452 ± 0.534	25.967 ± 2.124	0.007	0.40
LOC_Os08g07830	107.199 ± 7.856	28.008 ± 1.937	0.003	3.83
LOC_Os08g08130	0.017 ± 0.025	18.357 ± 0.892	0.001	0.00
LOC_Os08g08205	5.353 ± 0.449	12.113 ± 2.236	0.046	0.44
LOC_Os08g08690	10.435 ± 1.863	2.103 ± 0.653	0.016	4.96
LOC_Os08g09290	0.000 ± 0.000	12.752 ± 3.067	0.028	0.00
LOC_Os08g09840	28.177 ± 1.387	57.337 ± 5.761	0.015	0.49
LOC_Os08g09860	10.192 ± 2.763	22.315 ± 3.912	0.028	0.46
LOC_Os08g10480	14.890 ± 0.792	5.111 ± 1.178	0.001	2.91
LOC_Os08g13630	5.009 ± 0.443	11.229 ± 1.762	0.032	0.45
LOC_Os08g13690	31.486 ± 3.758	67.385 ± 10.279	0.027	0.47
LOC_Os08g13920	12.325 ± 1.577	1.457 ± 0.420	0.007	8.46
LOC_Os08g14350	51.793 ± 10.596	0.050 ± 0.071	0.020	1036.15
LOC_Os08g14450	26.001 ± 0.413	54.236 ± 4.970	0.015	0.48

LOC_Os08g14880	5.175 ± 0.082	24.808 ± 1.320	0.002	0.21
LOC_Os08g15266	43.571 ± 0.958	15.711 ± 0.445	0.000	2.77
LOC_Os08g15278	11.502 ± 1.307	2.286 ± 0.741	0.003	5.03
LOC_Os08g15460	5.225 ± 0.635	17.104 ± 0.990	0.000	0.31
LOC_Os08g15500	6.580 ± 0.305	13.711 ± 1.263	0.012	0.48
LOC_Os08g15650	14.803 ± 1.635	30.270 ± 2.738	0.005	0.49
LOC_Os08g19480	2.863 ± 0.256	18.477 ± 1.024	0.001	0.15
LOC_Os08g19670	16.825 ± 1.218	5.583 ± 0.971	0.001	3.01
LOC_Os08g24160	0.000 ± 0.000	12.220 ± 1.092	0.004	0.00
LOC_Os08g25050	3.714 ± 0.622	23.547 ± 1.458	0.001	0.16
LOC_Os08g25090	11.517 ± 0.680	23.790 ± 2.708	0.019	0.48
LOC_Os08g26230	36.193 ± 1.061	146.147 ± 6.534	0.001	0.25
LOC_Os08g26820	6.279 ± 0.520	19.434 ± 3.372	0.029	0.32
LOC_Os08g26870	51.934 ± 2.470	111.017 ± 10.155	0.011	0.47
LOC_Os08g28670	4.792 ± 0.675	14.226 ± 2.909	0.038	0.34
LOC_Os08g29020	7.450 ± 0.338	25.207 ± 3.114	0.014	0.30
LOC_Os08g29110	7.962 ± 0.284	20.017 ± 1.821	0.010	0.40
LOC_Os08g31219	16.082 ± 0.676	7.467 ± 2.258	0.025	2.15
LOC_Os08g31850	17.040 ± 1.479	4.015 ± 0.888	0.001	4.24
LOC_Os08g32930	34.833 ± 3.990	78.672 ± 6.412	0.002	0.44
LOC_Os08g33150	3.991 ± 0.254	16.117 ± 0.217	0.000	0.25
LOC_Os08g34010	11.700 ± 1.411	28.448 ± 1.911	0.001	0.41
LOC_Os08g35319	6.829 ± 1.427	14.785 ± 1.619	0.007	0.46
LOC_Os08g35420	472.751 ± 17.935	115.535 ± 21.895	0.000	4.09
LOC_Os08g37060	14.685 ± 1.025	6.990 ± 1.375	0.004	2.10
LOC_Os08g37370	92.756 ± 15.184	22.473 ± 1.617	0.022	4.13
LOC_Os08g37700	16.088 ± 0.646	36.492 ± 2.097	0.003	0.44
LOC_Os08g38086	9.452 ± 1.123	19.840 ± 3.032	0.028	0.48
LOC_Os08g38160	3.387 ± 0.766	10.775 ± 1.989	0.023	0.31
LOC_Os08g38910	11.017 ± 0.092	1.914 ± 0.127	0.000	5.76
LOC_Os08g39450	265.380 ± 17.627	107.839 ± 14.054	0.001	2.46
LOC_Os08g39840	5.588 ± 0.712	16.359 ± 0.361	0.000	0.34
LOC_Os08g40620	10.817 ± 0.045	25.176 ± 3.788	0.033	0.43
LOC_Os08g40870	7.873 ± 0.929	19.497 ± 2.572	0.015	0.40
LOC_Os08g41090	15.498 ± 1.019	41.493 ± 2.879	0.003	0.37
LOC_Os08g41280	55.828 ± 4.423	25.782 ± 3.139	0.002	2.17
LOC_Os08g41440	30.852 ± 1.921	3.931 ± 0.217	0.002	7.85
LOC_Os08g41880	10.110 ± 0.926	3.193 ± 0.582	0.002	3.17
LOC_Os08g41990	97.668 ± 4.747	225.933 ± 7.090	0.000	0.43
LOC_Os08g42800	30.037 ± 3.674	8.848 ± 2.387	0.004	3.39
LOC_Os08g43550	6.644 ± 0.892	23.943 ± 0.829	0.000	0.28
LOC_Os08g44480	24.688 ± 1.788	57.622 ± 3.320	0.001	0.43
LOC_Os08g44640	7.102 ± 0.246	15.520 ± 1.643	0.017	0.46
LOC_Os08g45190	40.974 ± 6.271	14.727 ± 2.549	0.016	2.78
LOC_Os09g00999	18905.977 ± 506.656	6355.795± 1311.531	0.002	2.97

LOC_Os09g01000	11336.240 ± 644.544	3483.679± 1290.297	0.005	3.25
LOC_Os09g04290	5.378 ± 0.683	10.872 ± 1.116	0.007	0.49
LOC_Os09g04300	9.045 ± 0.765	18.474 ± 1.492	0.004	0.49
LOC_Os09g04310	20.061 ± 2.157	40.329 ± 3.518	0.004	0.50
LOC_Os09g04430	7.018 ± 1.250	21.463 ± 3.011	0.011	0.33
LOC_Os09g04690	29.318 ± 6.588	2.768 ± 1.970	0.022	10.59
LOC_Os09g06740	7.058 ± 0.911	15.525 ± 2.788	0.039	0.45
LOC_Os09g09520	5.362 ± 0.628	11.527 ± 0.920	0.002	0.47
LOC_Os09g09830	14.325 ± 1.124	74.315 ± 11.423	0.017	0.19
LOC_Os09g10930	9.523 ± 0.078	20.748 ± 3.542	0.046	0.46
LOC_Os09g11480	16.177 ± 1.032	51.889 ± 6.019	0.012	0.31
LOC_Os09g13440	29.770 ± 1.404	87.789 ± 7.288	0.006	0.34
LOC_Os09g13570	76.844 ± 2.319	182.771 ± 15.305	0.009	0.42
LOC_Os09g13575	19.217 ± 5.519	41.933 ± 1.453	0.022	0.46
LOC_Os09g14614	11.713 ± 1.112	23.942 ± 2.583	0.011	0.49
LOC_Os09g16950	20.920 ± 1.990	60.287 ± 5.045	0.004	0.35
LOC_Os09g17329	0.002 ± 0.003	40.943 ± 2.817	0.002	0.00
LOC_Os09g17344	0.039 ± 0.027	11.765 ± 1.865	0.012	0.00
LOC_Os09g18594	0.010 ± 0.014	36.251 ± 2.782	0.003	0.00
LOC_Os09g19150	0.032 ± 0.023	12.975 ± 0.991	0.003	0.00
LOC_Os09g19160	0.370 ± 0.084	11.232 ± 1.607	0.011	0.03
LOC_Os09g19280	0.505 ± 0.091	14.556 ± 0.814	0.001	0.03
LOC_Os09g19310	0.581 ± 0.093	16.677 ± 2.329	0.010	0.03
LOC_Os09g19380	0.058 ± 0.083	19.974 ± 0.479	0.000	0.00
LOC_Os09g19390	0.031 ± 0.043	15.814 ± 3.755	0.027	0.00
LOC_Os09g19560	8.234 ± 1.066	16.538 ± 2.199	0.019	0.50
LOC_Os09g19820	7.014 ± 0.707	17.795 ± 0.241	0.001	0.39
LOC_Os09g19850	7.132 ± 0.638	14.283 ± 1.339	0.007	0.50
LOC_Os09g19952	29.600 ± 2.897	6.147 ± 0.653	0.006	4.82
LOC_Os09g19954	12.825 ± 1.583	2.420 ± 0.410	0.008	5.30
LOC_Os09g20390	15.786 ± 2.606	7.322 ± 0.513	0.040	2.16
LOC_Os09g20930	19.645 ± 3.056	9.501 ± 2.082	0.023	2.07
LOC_Os09g21180	3.944 ± 0.773	18.178 ± 1.398	0.001	0.22
LOC_Os09g21210	12.295 ± 0.873	1.560 ± 0.084	0.003	7.88
LOC_Os09g21710	31.433 ± 4.582	6.963 ± 1.029	0.014	4.51
LOC_Os09g23430	13.949 ± 4.145	49.973 ± 9.085	0.017	0.28
LOC_Os09g24406	21.735 ± 3.171	5.532 ± 0.728	0.015	3.93
LOC_Os09g24408	11.085 ± 1.577	2.163 ± 0.395	0.011	5.12
LOC_Os09g24412	44.420 ± 8.416	11.703 ± 3.294	0.020	3.80
LOC_Os09g25690	18.254 ± 2.418	2.128 ± 0.781	0.007	8.58
LOC_Os09g26620	74.935 ± 2.337	208.326 ± 23.890	0.015	0.36
LOC_Os09g26780	15.258 ± 1.918	4.321 ± 1.413	0.004	3.53
LOC_Os09g27010	24.733 ± 2.662	10.858 ± 1.152	0.009	2.28
LOC_Os09g27734	12.656 ± 1.324	29.908 ± 5.716	0.045	0.42
LOC_Os09g28440	27.766 ± 3.790	0.427 ± 0.167	0.009	64.96

LOC_Os09g28550	4.601 ± 0.973	10.020 ± 1.770	0.030	0.46
LOC_Os09g28640	10.564 ± 0.291	21.727 ± 3.238	0.039	0.49
LOC_Os09g28650	13.418 ± 2.790	65.158 ± 4.574	0.001	0.21
LOC_Os09g29270	0.000 ± 0.000	58.910 ± 1.059	0.000	0.00
LOC_Os09g29390	3.809 ± 0.745	11.139 ± 1.226	0.004	0.34
LOC_Os09g29520	26.808 ± 1.754	73.441 ± 10.923	0.024	0.37
LOC_Os09g29540	13.553 ± 0.345	45.340 ± 7.200	0.024	0.30
LOC_Os09g29560	4.884 ± 0.544	12.010 ± 2.212	0.038	0.41
LOC_Os09g29840	42.171 ± 1.173	84.593 ± 4.234	0.003	0.50
LOC_Os09g30130	4.601 ± 0.366	11.142 ± 0.725	0.002	0.41
LOC_Os09g30300	16.523 ± 1.227	3.003 ± 2.064	0.003	5.50
LOC_Os09g30340	236.692 ± 7.991	111.498 ± 10.026	0.000	2.12
LOC_Os09g30350	89.819 ± 4.924	43.803 ± 4.421	0.001	2.05
LOC_Os09g30418	49.414 ± 1.175	130.529 ± 5.236	0.001	0.38
LOC_Os09g30438	38.765 ± 2.135	99.691 ± 4.062	0.000	0.39
LOC_Os09g30466	7.377 ± 0.601	17.579 ± 0.842	0.000	0.42
LOC_Os09g31031	14.833 ± 0.261	3.211 ± 0.919	0.002	4.62
LOC_Os09g31200	301.278 ± 34.271	64.484 ± 2.150	0.010	4.67
LOC_Os09g32000	10.347 ± 0.495	2.849 ± 1.067	0.004	3.63
LOC_Os09g32080	11.154 ± 0.309	3.276 ± 1.580	0.017	3.41
LOC_Os09g32169	0.731 ± 0.195	174.960 ± 30.078	0.015	0.00
LOC_Os09g33470	15.189 ± 0.548	35.060 ± 1.715	0.002	0.43
LOC_Os09g33490	4.615 ± 0.257	10.613 ± 1.220	0.017	0.43
LOC_Os09g35010	109.578 ± 11.576	2.746 ± 1.162	0.005	39.91
LOC_Os09g35020	10.362 ± 1.623	0.867 ± 1.125	0.004	11.95
LOC_Os09g35030	29.807 ± 2.122	0.718 ± 0.399	0.002	41.51
LOC_Os09g37540	5.591 ± 0.626	11.278 ± 1.777	0.034	0.50
LOC_Os09g39230	8.929 ± 1.537	18.355 ± 0.786	0.005	0.49
LOC_Os09g39740	6.797 ± 0.321	18.574 ± 0.980	0.002	0.37
LOC_Os10g01470	28.900 ± 4.198	59.092 ± 6.999	0.011	0.49
LOC_Os10g02380	5.385 ± 0.346	12.261 ± 1.163	0.009	0.44
LOC_Os10g02480	22.954 ± 0.555	50.103 ± 3.161	0.006	0.46
LOC_Os10g04050	8.345 ± 1.242	16.719 ± 2.030	0.012	0.50
LOC_Os10g04570	2.448 ± 0.517	15.966 ± 0.657	0.000	0.15
LOC_Os10g04720	4.902 ± 0.755	12.764 ± 2.267	0.029	0.38
LOC_Os10g04730	11.980 ± 0.713	29.962 ± 3.485	0.015	0.40
LOC_Os10g05250	1.327 ± 0.128	35.916 ± 6.355	0.016	0.04
LOC_Os10g05780	5.956 ± 0.729	12.772 ± 0.851	0.001	0.47
LOC_Os10g09240	27.928 ± 3.359	13.456 ± 3.168	0.011	2.08
LOC_Os10g10175	7.760 ± 1.127	15.998 ± 2.612	0.032	0.49
LOC_Os10g10244	8.103 ± 0.303	17.256 ± 0.850	0.002	0.47
LOC_Os10g11870	4.365 ± 0.225	13.965 ± 1.712	0.014	0.31
LOC_Os10g14870	14.973 ± 0.583	33.807 ± 5.296	0.036	0.44
LOC_Os10g18490	4.971 ± 1.648	25.181 ± 0.181	0.003	0.20
LOC_Os10g20090	5.414 ± 0.276	14.331 ± 1.048	0.004	0.38

LOC_Os10g20310	9.073 ± 0.614	22.660 ± 1.951	0.006	0.40
LOC_Os10g20380	2.789 ± 0.470	10.974 ± 0.889	0.001	0.25
LOC_Os10g20480	0.008 ± 0.011	31.320 ± 0.846	0.000	0.00
LOC_Os10g21190	3497.965 ± 38.125	1204.542 ± 748.157	0.049	2.90
LOC_Os10g21192	444.797 ± 23.466	113.399 ± 18.834	0.000	3.92
LOC_Os10g21230	23.202 ± 4.695	9.237 ± 1.095	0.046	2.51
LOC_Os10g21238	30.153 ± 2.014	10.952 ± 2.305	0.001	2.75
LOC_Os10g21244	23.043 ± 0.541	5.663 ± 1.423	0.001	4.07
LOC_Os10g21248	25.364 ± 4.639	10.840 ± 1.679	0.035	2.34
LOC_Os10g21250	10.072 ± 1.026	5.024 ± 0.329	0.013	2.00
LOC_Os10g21266	19.717 ± 2.411	4.667 ± 0.073	0.013	4.23
LOC_Os10g21268	426.688 ± 8.657	82.223 ± 15.274	0.000	5.19
LOC_Os10g21352	33.121 ± 3.729	9.231 ± 3.803	0.003	3.59
LOC_Os10g21372	22.444 ± 2.568	9.782 ± 1.823	0.006	2.29
LOC_Os10g21394	17.621 ± 0.965	4.483 ± 0.559	0.000	3.93
LOC_Os10g21406	126.822 ± 6.505	45.603 ± 11.758	0.003	2.78
LOC_Os10g21418	10.066 ± 1.556	4.463 ± 0.420	0.030	2.26
LOC_Os10g21560	5.732 ± 0.390	11.675 ± 1.101	0.010	0.49
LOC_Os10g24970	4.915 ± 1.044	10.284 ± 1.937	0.039	0.48
LOC_Os10g25230	28.994 ± 0.507	0.873 ± 0.420	0.000	33.21
LOC_Os10g25290	27.883 ± 2.634	4.327 ± 1.086	0.002	6.44
LOC_Os10g25430	5.765 ± 0.394	17.838 ± 1.604	0.006	0.32
LOC_Os10g27340	16.496 ± 1.089	38.378 ± 2.985	0.005	0.43
LOC_Os10g28050	22.109 ± 1.636	63.451 ± 3.475	0.001	0.35
LOC_Os10g28080	3.683 ± 0.674	12.064 ± 1.774	0.013	0.31
LOC_Os10g28210	6.185 ± 0.248	18.941 ± 3.268	0.031	0.33
LOC_Os10g30410	8.160 ± 0.348	16.885 ± 0.741	0.001	0.48
LOC_Os10g30880	12.643 ± 1.423	29.270 ± 2.771	0.005	0.43
LOC_Os10g31320	86.958 ± 15.105	19.532 ± 4.490	0.018	4.45
LOC_Os10g31330	269.796 ± 35.787	45.173 ± 1.719	0.012	5.97
LOC_Os10g32444	5.058 ± 0.744	11.488 ± 2.296	0.047	0.44
LOC_Os10g32520	4.696 ± 0.269	10.250 ± 0.949	0.010	0.46
LOC_Os10g32730	7.989 ± 0.624	18.624 ± 2.547	0.022	0.43
LOC_Os10g32770	12.434 ± 0.713	25.645 ± 1.627	0.003	0.48
LOC_Os10g32810	25.927 ± 1.473	8.004 ± 0.523	0.001	3.24
LOC_Os10g33210	4.065 ± 0.065	11.166 ± 0.943	0.008	0.36
LOC_Os10g33990	22.994 ± 4.457	10.860 ± 1.708	0.047	2.12
LOC_Os10g34180	6.568 ± 0.153	24.822 ± 1.374	0.003	0.26
LOC_Os10g34409	0.241 ± 0.134	21.154 ± 2.017	0.004	0.01
LOC_Os10g34795	33.387 ± 8.979	76.813 ± 2.919	0.014	0.43
LOC_Os10g34940	4.515 ± 0.684	13.153 ± 1.397	0.005	0.34
LOC_Os10g35140	6.779 ± 0.914	14.395 ± 1.865	0.015	0.47
LOC_Os10g35770	5.372 ± 0.666	19.275 ± 1.729	0.003	0.28
LOC_Os10g36260	87.029 ± 2.496	191.426 ± 22.669	0.022	0.45
LOC_Os10g36500	64.272 ± 12.854	5.604 ± 1.027	0.023	11.47

LOC_Os10g36580	71.439 ± 10.693	9.338 ± 1.906	0.012	7.65
LOC_Os10g36860	11.270 ± 0.823	23.427 ± 2.180	0.009	0.48
LOC_Os10g37340	24.806 ± 1.141	7.033 ± 1.256	0.000	3.53
LOC_Os10g38110	31.554 ± 0.677	63.458 ± 1.716	0.000	0.50
LOC_Os10g38229	10.026 ± 1.539	4.250 ± 0.678	0.020	2.36
LOC_Os10g38234	25.379 ± 3.930	11.314 ± 0.849	0.032	2.24
LOC_Os10g38238	21.091 ± 2.931	6.121 ± 0.782	0.014	3.45
LOC_Os10g38272	29.612 ± 2.648	12.504 ± 1.798	0.003	2.37
LOC_Os10g38274	12.935 ± 2.069	4.101 ± 1.025	0.013	3.15
LOC_Os10g38276	14.502 ± 2.763	4.775 ± 2.045	0.019	3.04
LOC_Os10g38470	5.256 ± 0.885	11.395 ± 1.052	0.004	0.46
LOC_Os10g38780	39.794 ± 1.150	89.834 ± 2.946	0.000	0.44
LOC_Os10g39010	11.717 ± 0.678	27.235 ± 1.359	0.001	0.43
LOC_Os10g39140	27.640 ± 1.418	104.694 ± 4.575	0.001	0.26
LOC_Os10g39880	117.030 ± 14.834	22.697 ± 5.950	0.006	5.16
LOC_Os10g40030	16.486 ± 0.864	37.482 ± 0.823	0.000	0.44
LOC_Os10g40130	6.225 ± 0.303	16.982 ± 1.839	0.013	0.37
LOC_Os10g40220	0.033 ± 0.031	10.580 ± 0.960	0.004	0.00
LOC_Os10g41689	24.572 ± 2.268	9.466 ± 0.735	0.007	2.60
LOC_Os10g42030	4.424 ± 0.238	17.402 ± 2.130	0.012	0.25
LOC_Os10g42040	32.698 ± 2.203	131.617 ± 16.623	0.013	0.25
LOC_Os10g42700	7.997 ± 0.064	17.809 ± 0.427	0.001	0.45
LOC_Os10g42960	14.805 ± 0.358	32.718 ± 0.825	0.000	0.45
LOC_Os10g43050	29.343 ± 0.758	61.658 ± 5.939	0.015	0.48
LOC_Os10g43060	14.673 ± 0.179	4.832 ± 0.626	0.001	3.04
LOC_Os11g01890	6.286 ± 0.798	15.397 ± 1.676	0.007	0.41
LOC_Os11g02730	12.759 ± 0.664	4.361 ± 1.826	0.014	2.93
LOC_Os11g05380	10.407 ± 0.287	0.025 ± 0.035	0.000	420.11
LOC_Os11g05552	5.940 ± 0.566	12.827 ± 1.013	0.003	0.46
LOC_Os11g05614	10.215 ± 0.704	2.097 ± 0.657	0.000	4.87
LOC_Os11g05860	112.925 ± 2.089	47.903 ± 5.152	0.001	2.36
LOC_Os11g06020	38.874 ± 3.540	90.280 ± 1.327	0.001	0.43
LOC_Os11g06780	7.422 ± 0.770	17.100 ± 2.761	0.031	0.43
LOC_Os11g06980	3.193 ± 0.173	12.312 ± 1.942	0.021	0.26
LOC_Os11g07670	4.565 ± 0.331	10.359 ± 0.643	0.002	0.44
LOC_Os11g07860	2.199 ± 0.128	11.051 ± 1.075	0.007	0.20
LOC_Os11g07910	6.066 ± 1.259	12.463 ± 1.381	0.009	0.49
LOC_Os11g07930	4.179 ± 0.129	19.744 ± 0.406	0.000	0.21
LOC_Os11g07960	11.380 ± 0.724	33.583 ± 4.536	0.018	0.34
LOC_Os11g07980	23.688 ± 2.115	84.050 ± 3.835	0.000	0.28
LOC_Os11g08300	14.474 ± 0.306	63.449 ± 4.306	0.004	0.23
LOC_Os11g09590	7.434 ± 0.340	18.782 ± 2.193	0.016	0.40
LOC_Os11g10320	132.890 ± 12.375	273.528 ± 38.584	0.027	0.49
LOC_Os11g10470	117.450 ± 11.259	29.932 ± 7.136	0.002	3.92
LOC_Os11g10480	11.360 ± 1.206	4.744 ± 0.949	0.004	2.39

LOC_Os11g10850	30.442 ± 7.161	83.594 ± 7.615	0.002	0.36
LOC_Os11g12340	1.025 ± 0.042	16.781 ± 1.399	0.004	0.06
LOC_Os11g12590	9.674 ± 0.664	23.361 ± 0.689	0.000	0.41
LOC_Os11g13390	5.967 ± 0.466	26.494 ± 1.517	0.001	0.23
LOC_Os11g13420	4.404 ± 0.984	29.233 ± 3.349	0.006	0.15
LOC_Os11g13680	3.598 ± 0.907	11.579 ± 0.371	0.002	0.31
LOC_Os11g16550	32.154 ± 1.200	6.879 ± 1.149	0.000	4.67
LOC_Os11g17290	25.081 ± 1.013	51.124 ± 2.516	0.002	0.49
LOC_Os11g20310	8.999 ± 0.673	19.407 ± 2.233	0.016	0.46
LOC_Os11g25040	41.362 ± 3.343	13.913 ± 1.846	0.002	2.97
LOC_Os11g25130	7.685 ± 0.395	18.719 ± 1.446	0.006	0.41
LOC_Os11g27730	10.999 ± 0.788	25.879 ± 1.861	0.003	0.43
LOC_Os11g28600	66.848 ± 2.694	149.438 ± 20.499	0.028	0.45
LOC_Os11g29970	4.286 ± 0.102	11.959 ± 0.581	0.002	0.36
LOC_Os11g30200	22.319 ± 1.965	47.472 ± 4.926	0.010	0.47
LOC_Os11g30210	8.117 ± 0.447	18.351 ± 1.320	0.004	0.44
LOC_Os11g31060	79.957 ± 19.940	6.323 ± 0.654	0.035	12.65
LOC_Os11g31530	4.476 ± 0.210	25.224 ± 2.975	0.010	0.18
LOC_Os11g32770	12.694 ± 2.899	1.195 ± 0.406	0.028	10.63
LOC_Os11g33394	7.840 ± 0.623	36.558 ± 1.853	0.001	0.21
LOC_Os11g37230	41.578 ± 1.321	87.395 ± 3.977	0.002	0.48
LOC_Os11g37960	24.299 ± 2.438	4.126 ± 0.914	0.003	5.89
LOC_Os11g38020	8.040 ± 0.517	23.386 ± 0.553	0.000	0.34
LOC_Os11g38210	0.366 ± 0.272	20.393 ± 4.985	0.029	0.02
LOC_Os11g38330	0.012 ± 0.017	29.330 ± 1.405	0.001	0.00
LOC_Os11g39209	6.429 ± 0.575	15.631 ± 2.888	0.041	0.41
LOC_Os11g39310	6.113 ± 0.145	12.432 ± 0.968	0.010	0.49
LOC_Os11g39370	42.173 ± 2.039	109.603 ± 2.088	0.000	0.38
LOC_Os11g40770	0.000 ± 0.000	12.693 ± 3.600	0.038	0.00
LOC_Os11g40930	0.000 ± 0.000	15.966 ± 1.991	0.008	0.00
LOC_Os11g40970	0.156 ± 0.102	41.286 ± 2.396	0.002	0.00
LOC_Os11g41034	0.812 ± 0.098	34.208 ± 0.621	0.000	0.02
LOC_Os11g41910	13.892 ± 0.068	28.788 ± 0.890	0.002	0.48
LOC_Os11g42030	0.000 ± 0.000	11.344 ± 1.691	0.011	0.00
LOC_Os11g42500	11.558 ± 2.688	1.122 ± 0.433	0.029	10.30
LOC_Os11g42970	6.242 ± 1.027	28.831 ± 6.659	0.038	0.22
LOC_Os11g43600	4.950 ± 0.382	11.183 ± 1.112	0.009	0.44
LOC_Os11g44870	31.262 ± 0.545	73.462 ± 4.264	0.005	0.43
LOC_Os11g45840	20.440 ± 0.937	41.488 ± 1.369	0.000	0.49
LOC_Os11g45850	20.239 ± 1.281	44.913 ± 4.683	0.013	0.45
LOC_Os11g45890	11.089 ± 2.104	33.819 ± 3.093	0.002	0.33
LOC_Os11g46810	1.714 ± 0.245	10.407 ± 0.402	0.000	0.16
LOC_Os11g47240	11.273 ± 1.111	30.277 ± 0.102	0.002	0.37
LOC_Os11g47269	4.985 ± 0.819	12.783 ± 1.117	0.002	0.39
LOC_Os11g47460	7.542 ± 1.466	16.447 ± 2.319	0.015	0.46

LOC_Os11g47870	6.136 ± 1.126	14.409 ± 1.536	0.005	0.43
LOC_Os12g01160	10.127 ± 0.768	22.659 ± 1.332	0.001	0.45
LOC_Os12g01950	4.349 ± 0.551	11.595 ± 1.774	0.021	0.38
LOC_Os12g02040	10.209 ± 0.396	1.396 ± 0.667	0.000	7.32
LOC_Os12g02310	12.552 ± 1.069	0.684 ± 0.503	0.001	18.34
LOC_Os12g02330	150.342 ± 10.856	380.720 ± 31.042	0.005	0.39
LOC_Os12g02960	15.839 ± 1.645	50.200 ± 2.573	0.000	0.32
LOC_Os12g02980	19.380 ± 1.031	6.863 ± 0.754	0.000	2.82
LOC_Os12g03350	20.240 ± 2.451	41.022 ± 3.977	0.006	0.49
LOC_Os12g03370	28.129 ± 2.363	12.282 ± 0.508	0.009	2.29
LOC_Os12g03420	21.648 ± 0.540	51.314 ± 6.537	0.023	0.42
LOC_Os12g05050	30.778 ± 2.542	70.752 ± 2.933	0.000	0.44
LOC_Os12g05260	14.459 ± 0.392	5.468 ± 1.664	0.013	2.64
LOC_Os12g06850	9.586 ± 0.650	22.103 ± 3.973	0.044	0.43
LOC_Os12g07180	0.017 ± 0.024	10.928 ± 0.849	0.003	0.00
LOC_Os12g07210	17.983 ± 1.901	6.595 ± 0.507	0.010	2.73
LOC_Os12g07490	6.093 ± 1.195	13.708 ± 1.327	0.004	0.44
LOC_Os12g08090	36.491 ± 2.113	11.606 ± 0.979	0.001	3.14
LOC_Os12g08130	24.784 ± 1.582	6.199 ± 1.977	0.001	4.00
LOC_Os12g08830	6.310 ± 0.391	14.027 ± 2.091	0.031	0.45
LOC_Os12g09584	5.735 ± 0.830	15.620 ± 2.190	0.014	0.37
LOC_Os12g10184	6.083 ± 0.444	13.287 ± 0.749	0.001	0.46
LOC_Os12g10580	148.451 ± 6.012	28.662 ± 4.753	0.000	5.18
LOC_Os12g10710	1.583 ± 0.186	17.609 ± 1.432	0.004	0.09
LOC_Os12g10740	13.503 ± 0.340	30.651 ± 0.688	0.000	0.44
LOC_Os12g11660	7.637 ± 0.897	25.020 ± 2.412	0.005	0.31
LOC_Os12g11790	0.031 ± 0.044	13.201 ± 2.038	0.012	0.00
LOC_Os12g11830	10.269 ± 2.016	52.717 ± 1.897	0.000	0.19
LOC_Os12g12000	0.116 ± 0.164	32.747 ± 3.189	0.005	0.00
LOC_Os12g12090	0.000 ± 0.000	162.834 ± 11.809	0.003	0.00
LOC_Os12g12110	1495.893 ± 200.364	461.441 ± 30.428	0.016	3.24
LOC_Os12g12115	0.000 ± 0.000	99.122 ± 9.653	0.005	0.00
LOC_Os12g12120	0.105 ± 0.094	10.284 ± 0.671	0.002	0.01
LOC_Os12g12130	0.013 ± 0.018	14.313 ± 2.358	0.013	0.00
LOC_Os12g12170	6.332 ± 0.578	22.979 ± 2.848	0.012	0.28
LOC_Os12g12560	0.941 ± 0.114	16.156 ± 1.904	0.008	0.06
LOC_Os12g12690	0.000 ± 0.000	17.445 ± 3.564	0.020	0.00
LOC_Os12g12850	95.476 ± 3.345	282.857 ± 8.311	0.000	0.34
LOC_Os12g13290	3.687 ± 0.389	13.510 ± 1.008	0.002	0.27
LOC_Os12g13460	3.429 ± 0.289	13.641 ± 1.118	0.004	0.25
LOC_Os12g13674	0.216 ± 0.132	43.230 ± 1.847	0.001	0.01
LOC_Os12g14540	0.000 ± 0.000	19.066 ± 1.641	0.004	0.00
LOC_Os12g14840	0.216 ± 0.119	14.758 ± 1.050	0.002	0.01
LOC_Os12g15505	0.000 ± 0.000	112.377 ± 13.816	0.007	0.00
LOC_Os12g16200	11.248 ± 0.610	24.485 ± 0.771	0.000	0.46

LOC_Os12g16220	4.215 ± 0.534	10.382 ± 1.242	0.010	0.41
LOC_Os12g16240	21.027 ± 2.203	7.073 ± 0.937	0.005	2.97
LOC_Os12g16280	0.731 ± 0.023	18.080 ± 0.473	0.000	0.04
LOC_Os12g16350	4.885 ± 0.370	13.153 ± 1.362	0.009	0.37
LOC_Os12g16520	7.855 ± 0.340	17.718 ± 0.767	0.001	0.44
LOC_Os12g16524	8.917 ± 0.490	24.136 ± 1.866	0.005	0.37
LOC_Os12g16540	9.042 ± 1.645	22.053 ± 4.011	0.030	0.41
LOC_Os12g17120	0.091 ± 0.064	12.471 ± 3.203	0.032	0.01
LOC_Os12g18630	10.629 ± 0.522	23.015 ± 1.561	0.004	0.46
LOC_Os12g19030	4.268 ± 0.280	10.073 ± 0.464	0.000	0.42
LOC_Os12g19580	33.874 ± 1.338	10.828 ± 2.137	0.001	3.13
LOC_Os12g20144	6.091 ± 0.309	12.463 ± 0.968	0.007	0.49
LOC_Os12g20410	10.275 ± 1.168	3.728 ± 1.301	0.006	2.76
LOC_Os12g22810	2.824 ± 0.240	11.310 ± 1.007	0.005	0.25
LOC_Os12g22839	15.860 ± 1.701	75.146 ± 17.203	0.038	0.21
LOC_Os12g22870	12.717 ± 2.590	0.054 ± 0.077	0.020	234.73
LOC_Os12g23150	0.750 ± 0.167	16.617 ± 2.316	0.010	0.05
LOC_Os12g23780	0.092 ± 0.075	14.849 ± 0.774	0.001	0.01
LOC_Os12g24320	24.231 ± 3.540	87.529 ± 8.004	0.003	0.28
LOC_Os12g25160	0.000 ± 0.000	10.928 ± 1.887	0.015	0.00
LOC_Os12g25170	8.916 ± 1.553	20.467 ± 1.131	0.002	0.44
LOC_Os12g25280	0.000 ± 0.000	13.043 ± 2.982	0.025	0.00
LOC_Os12g25800	2.427 ± 0.984	10.237 ± 0.619	0.002	0.24
LOC_Os12g27220	147.994 ± 5.587	0.506 ± 0.244	0.001	292.44
LOC_Os12g27254	28.985 ± 0.885	0.202 ± 0.083	0.000	143.73
LOC_Os12g29330	20.463 ± 1.491	46.874 ± 4.155	0.007	0.44
LOC_Os12g29400	12.745 ± 2.594	3.410 ± 0.195	0.036	3.74
LOC_Os12g29950	13.514 ± 1.345	30.048 ± 3.506	0.013	0.45
LOC_Os12g31460	30.289 ± 2.275	61.431 ± 6.239	0.012	0.49
LOC_Os12g32610	21.550 ± 5.968	1.394 ± 0.532	0.040	15.46
LOC_Os12g33130	54.277 ± 0.550	126.239 ± 3.644	0.001	0.43
LOC_Os12g33922	4.834 ± 1.730	10.084 ± 0.672	0.037	0.48
LOC_Os12g34500	28.051 ± 4.517	13.633 ± 1.194	0.038	2.06
LOC_Os12g34510	17.895 ± 1.177	6.187 ± 1.882	0.003	2.89
LOC_Os12g35630	47.504 ± 3.252	111.149 ± 4.579	0.000	0.43
LOC_Os12g35890	4.318 ± 0.474	10.594 ± 1.985	0.040	0.41
LOC_Os12g36210	24.621 ± 2.023	0.168 ± 0.238	0.003	146.42
LOC_Os12g36830	54.016 ± 2.908	151.012 ± 9.560	0.003	0.36
LOC_Os12g36850	18.984 ± 0.902	70.459 ± 3.428	0.001	0.27
LOC_Os12g36860	6.204 ± 1.304	27.493 ± 1.128	0.000	0.23
LOC_Os12g36880	74.200 ± 5.188	252.485 ± 30.701	0.013	0.29
LOC_Os12g36910	10.317 ± 0.676	5.009 ± 0.633	0.001	2.06
LOC_Os12g37260	663.737 ± 36.570	33.447 ± 1.129	0.002	19.84
LOC_Os12g37350	53.549 ± 1.585	5.939 ± 0.655	0.000	9.02
LOC_Os12g37415	53.380 ± 12.754	128.847 ± 15.742	0.007	0.41

LOC_Os12g38490	23.514 ± 0.630	47.637 ± 0.797	0.000	0.49
LOC_Os12g38660	15.670 ± 1.330	42.029 ± 4.958	0.013	0.37
LOC_Os12g38720	22.024 ± 1.458	46.270 ± 1.488	0.000	0.48
LOC_Os12g39120	5.225 ± 0.498	12.054 ± 1.451	0.014	0.43
LOC_Os12g39370	19.889 ± 0.310	40.288 ± 2.654	0.008	0.49
LOC_Os12g39770	1.559 ± 0.600	24.542 ± 2.987	0.007	0.06
LOC_Os12g40180	18.512 ± 5.516	2.658 ± 1.502	0.047	6.96
LOC_Os12g41560	2.391 ± 0.203	22.692 ± 4.408	0.023	0.11
LOC_Os12g42150	6.395 ± 0.510	13.401 ± 1.491	0.014	0.48
LOC_Os12g42220	6.148 ± 0.296	17.988 ± 1.850	0.010	0.34
LOC_Os12g42876	14.887 ± 0.936	35.327 ± 0.258	0.000	0.42
LOC_Os12g43410	16.478 ± 0.735	51.675 ± 9.914	0.037	0.32
LOC_Os12g43440	5.886 ± 1.323	21.028 ± 3.046	0.010	0.28
LOC_Os12g43550	5.113 ± 0.152	11.863 ± 0.859	0.007	0.43
LOC_Os12g43600	2562.011 ± 370.410	6951.678 ± 313.125	0.000	0.37
LOC_Os12g43640	2.618 ± 0.057	11.829 ± 0.359	0.001	0.22
LOC_Os12g43810	4.775 ± 0.675	13.003 ± 2.496	0.036	0.37
LOC_Os12g43880	10.887 ± 0.983	31.639 ± 4.453	0.018	0.34
ChrSy.fgenesh.gene.14	54.749 ± 2.238	197.501 ± 3.229	0.000	0.28
