# Supplementary material for: Integrative Analysis of the microRNAome and Transcriptome Illuminates the Response of Susceptible Rice Plants to Rice Stripe Virus
Source: PLoS One. 2016 Jan 22;11(1):e0146946. doi: 10.1371/journal.pone.0146946 (PMC4723043; doi:10.1371/journal.pone.0146946)
Supplement: S6 Table — (PDF) [file pone.0146946.s006.pdf]

S6 Table. The miRNAs negatively correlated with their target genes

miRNAs		Genes		
**Name**	**up/down**	**ID**	**up/down**	**Description**
osa-miR1320-3p	up	LOC_Os11g47240	down	Probable LRR receptor-like serine/threonine-protein kinase
osa-miR1320-3p	up	LOC_Os12g23150	down	4-nitrophenylphosphatase
osa-miR1320-5p	up	LOC_Os01g71340	down	Glucan endo-1,3-beta-glucosidase, acidic isoform
osa-miR1423-5p	up	LOC_Os10g20310	down	Uncharacterized protein
osa-miR1423-5p	up	LOC_Os10g36260	down	Uncharacterized protein
osa-miR1423-5p	up	LOC_Os10g35140	down	Uncharacterized transporter sll0355
osa-miR1423-5p	up	LOC_Os07g39780	down	SUMO-activating enzyme subunit 2
osa-miR1423-5p	up	LOC_Os04g43710	down	Phosphoenolpyruvate carboxylase kinase 2
osa-miR1423-5p	up	LOC_Os02g37090	down	Protein PHYLLO, chloroplastic
osa-miR1423-5p	up	LOC_Os02g37090	down	Protein PHYLLO, chloroplastic
osa-miR1423-5p	up	LOC_Os01g47450	down	Carboxyl-terminal-processing protease
osa-miR1423-5p	up	LOC_Os06g34400	down	RING-H2 finger protein ATL57
osa-miR1423-5p	up	LOC_Os05g35980	down	Uncharacterized protein
osa-miR1423-5p	up	LOC_Os06g38450	down	Vignain
osa-miR1423-5p	up	LOC_Os09g19280	down	Disease resistance protein RPM1
osa-miR1425-3p	up	LOC_Os07g03040	down	Putative uncharacterized protein
osa-miR1425-3p	up	LOC_Os01g49830	down	AP2/ERF domain-containing protein
osa-miR1425-3p	up	LOC_Os11g45890	down	Uncharacterized protein
osa-miR1429-5p	up	LOC_Os07g35680	down	Cysteine-rich receptor-like protein kinase 8
osa-miR1429-5p	up	LOC_Os11g45890	down	Uncharacterized protein
osa-miR1432-3p	up	LOC_Os02g42160	down	Wall-associated receptor kinase-like 1
osa-miR1432-3p	up	LOC_Os07g35680	down	Cysteine-rich receptor-like protein kinase 8
osa-miR1432-5p	up	LOC_Os12g39120	down	Probable protein phosphatase 2C 78
osa-miR1432-5p	up	LOC_Os02g57280	down	Brown planthopper-induced resistance protein 6

osa-miR1432-5p	up	LOC_Os12g16200	down	Glutathione synthetase, chloroplastic
osa-miR1432-5p	up	LOC_Os06g02500	down	Superoxide dismutase [Fe], chloroplastic (Fragment)
osa-miR156a	down	LOC_Os08g07080	up	Zingiberene synthase
osa-miR156a	down	LOC_Os01g01660	up	Isoflavone reductase homolog IRL
osa-miR156a	down	LOC_Os10g25230	up	ZIM domain containing protein, putative, expressed
osa-miR156c-3p	up	LOC_Os01g02400	down	Probable receptor-like protein kinase
osa-miR156c-3p	up	LOC_Os10g04720	down	G-type lectin S-receptor-like serine/threonine-protein kinase B120
osa-miR156c-3p	up	LOC_Os12g14540	down	Uncharacterized protein
osa-miR156d	down	LOC_Os08g07080	up	Zingiberene synthase
osa-miR156d	down	LOC_Os01g01660	up	Isoflavone reductase homolog IRL
osa-miR156d	down	LOC_Os10g25230	up	ZIM domain containing protein, putative, expressed
osa-miR156e	down	LOC_Os08g07080	up	Zingiberene synthase
osa-miR156e	down	LOC_Os01g01660	up	Isoflavone reductase homolog IRL
osa-miR156e	down	LOC_Os10g25230	up	ZIM domain containing protein, putative, expressed
osa-miR156f-5p	down	LOC_Os08g07080	up	Zingiberene synthase
osa-miR156f-5p	down	LOC_Os01g01660	up	Isoflavone reductase homolog IRL
osa-miR156f-5p	down	LOC_Os10g25230	up	ZIM domain containing protein, putative, expressed
osa-miR156g-3p	up	LOC_Os01g02400	down	Probable receptor-like protein kinase
osa-miR156g-3p	up	LOC_Os10g04720	down	G-type lectin S-receptor-like serine/threonine-protein kinase B120
osa-miR156g-3p	up	LOC_Os12g14540	down	Uncharacterized protein
osa-miR156h-5p	down	LOC_Os08g07080	up	Zingiberene synthase
osa-miR156h-5p	down	LOC_Os01g01660	up	Isoflavone reductase homolog IRL
osa-miR156h-5p	down	LOC_Os10g25230	up	ZIM domain containing protein, putative, expressed
osa-miR156i	down	LOC_Os08g07080	up	Zingiberene synthase
osa-miR156i	down	LOC_Os01g01660	up	Isoflavone reductase homolog IRL
osa-miR156i	down	LOC_Os10g25230	up	ZIM domain containing protein, putative, expressed
osa-miR156j-3p	up	LOC_Os02g38170	down	Uncharacterized protein

osa-miR156j-3p	up	LOC_Os02g38170	down	Uncharacterized protein
osa-miR156j-3p	up	LOC_Os02g40180	down	Probable LRR receptor-like serine/threonine-protein kinase
osa-miR156j-5p	down	LOC_Os08g07080	up	Zingiberene synthase
osa-miR156j-5p	down	LOC_Os01g01660	up	Isoflavone reductase homolog IRL
osa-miR156j-5p	down	LOC_Os10g25230	up	ZIM domain containing protein, putative, expressed
osa-miR156k	down	LOC_Os08g07080	up	Zingiberene synthase
osa-miR156k	down	LOC_Os01g01660	up	Isoflavone reductase homolog IRL
osa-miR156k	down	LOC_Os10g25230	up	ZIM domain containing protein, putative, expressed
osa-miR159a.1	up	LOC_Os11g07910	down	Transmembrane 9 superfamily member 3
osa-miR159a.1	up	LOC_Os04g40420	down	SWI/SNF complex subunit SWI3A
osa-miR159a.1	up	LOC_Os07g30020	down	Multiple C2 and transmembrane domain-containing protein 1
osa-miR159a.1	up	LOC_Os10g04730	down	Cysteine-rich receptor-like protein kinase 5
osa-miR159a.1	up	LOC_Os11g27730	down	indole-2-monooxygenase
osa-miR159a.1	up	LOC_Os11g40970	down	Probable LRR receptor-like serine/threonine-protein kinase
osa-miR159a.1	up	LOC_Os11g40930	down	Uncharacterized protein
osa-miR159a.2	up	LOC_Os01g71340	down	Glucan endo-1,3-beta-glucosidase, acidic isoform
osa-miR159a.2	up	LOC_Os01g02700	down	Probable receptor-like protein kinase
osa-miR159a.2	up	LOC_Os01g02400	down	Probable receptor-like protein kinase
osa-miR159a.2	up	LOC_Os09g13570	down	CPuORF2 - conserved peptide uORF-containing transcript, expressed
osa-miR159a.2	up	LOC_Os08g44640	down	Serine carboxypeptidase-like 51
osa-miR159a.2	up	LOC_Os03g46070	down	Zeamatin
osa-miR159a.2	up	LOC_Os02g17710	down	Probable leucine-rich repeat receptor-like protein kinase
osa-miR159a.2	up	LOC_Os07g11280	down	Pentatricopeptide repeat-containing protein
osa-miR159a.2	up	LOC_Os07g33480	down	Cytochrome P450 716B1
osa-miR159a.2	up	LOC_Os04g45710	down	Uncharacterized protein
osa-miR159a.2	up	LOC_Os11g06020	down	BEL1-like homeodomain protein 1
osa-miR159a.2	up	LOC_Os06g05050	down	Wall-associated receptor kinase 3

osa-miR159a.2	up	LOC_Os03g24930	down	Probable receptor-like protein kinase
osa-miR159a.2	up	LOC_Os01g70710	down	heavy metal-associated domain containing protein
osa-miR159a.2	up	LOC_Os11g07980	down	Ankyrin repeat domain-containing protein 6
osa-miR159a.2	up	LOC_Os02g03960	down	Ocs element-binding factor 1
osa-miR159a.2	up	LOC_Os07g13530	down	Ras-related protein RABE1c
osa-miR159a.2	up	LOC_Os08g01240	down	Uncharacterized protein
osa-miR159a.2	up	LOC_Os07g05840	down	Uncharacterized protein
osa-miR159a.2	up	LOC_Os12g14840	down	Uncharacterized protein
osa-miR159a.2	up	LOC_Os04g24804	down	Uncharacterized protein
osa-miR159a.2	up	LOC_Os11g40970	down	Probable LRR receptor-like serine/threonine-protein kinase
osa-miR159b	up	LOC_Os11g07910	down	Transmembrane 9 superfamily member 3
osa-miR159b	up	LOC_Os04g40420	down	SWI/SNF complex subunit SWI3A
osa-miR159b	up	LOC_Os07g30020	down	Multiple C2 and transmembrane domain-containing protein 1
osa-miR159b	up	LOC_Os10g04730	down	Cysteine-rich receptor-like protein kinase 5
osa-miR159b	up	LOC_Os11g27730	down	indole-2-monooxygenase
osa-miR159b	up	LOC_Os11g40970	down	Probable LRR receptor-like serine/threonine-protein kinase
osa-miR159b	up	LOC_Os11g40930	down	Uncharacterized protein
osa-miR159d	up	LOC_Os11g07910	down	Transmembrane 9 superfamily member 3
osa-miR159d	up	LOC_Os04g40420	down	SWI/SNF complex subunit SWI3A
osa-miR159d	up	LOC_Os03g27830	down	Uncharacterized protein
osa-miR159d	up	LOC_Os09g29520	down	Wall-associated receptor kinase 3
osa-miR159d	up	LOC_Os07g30020	down	Multiple C2 and transmembrane domain-containing protein 1
osa-miR159d	up	LOC_Os10g04730	down	Cysteine-rich receptor-like protein kinase 5
osa-miR159d	up	LOC_Os11g27730	down	indole-2-monooxygenase
osa-miR159d	up	LOC_Os11g40970	down	Probable LRR receptor-like serine/threonine-protein kinase
osa-miR159d	up	LOC_Os06g21740	down	Transposon Ty3-G Gag-Pol polyprotein
osa-miR159d	up	LOC_Os11g40930	down	Uncharacterized protein

osa-miR159e	up	LOC_Os11g07910	down	Transmembrane 9 superfamily member 3
osa-miR159e	up	LOC_Os04g40420	down	SWI/SNF complex subunit SWI3A
osa-miR159e	up	LOC_Os03g27830	down	Uncharacterized protein
osa-miR159e	up	LOC_Os09g29520	down	Wall-associated receptor kinase 3
osa-miR159e	up	LOC_Os07g30020	down	Multiple C2 and transmembrane domain-containing protein 1
osa-miR159e	up	LOC_Os10g04730	down	Cysteine-rich receptor-like protein kinase 5
osa-miR159e	up	LOC_Os11g27730	down	indole-2-monooxygenase
osa-miR159e	up	LOC_Os11g40970	down	Probable LRR receptor-like serine/threonine-protein kinase
osa-miR159e	up	LOC_Os11g40930	down	Uncharacterized protein
osa-miR160a-3p	up	LOC_Os10g20310	down	Uncharacterized protein
osa-miR160a-3p	up	LOC_Os04g40420	down	SWI/SNF complex subunit SWI3A
osa-miR160a-3p	up	LOC_Os12g01950	down	Uncharacterized protein
osa-miR160a-3p	up	LOC_Os06g15330	down	Zinc finger protein CONSTANS-LIKE 16
osa-miR160b-3p	up	LOC_Os10g20310	down	Uncharacterized protein
osa-miR160b-3p	up	LOC_Os04g40420	down	SWI/SNF complex subunit SWI3A
osa-miR160b-3p	up	LOC_Os12g01950	down	Uncharacterized protein
osa-miR160b-3p	up	LOC_Os06g15330	down	Zinc finger protein CONSTANS-LIKE 16
osa-miR160c-3p	up	LOC_Os10g20310	down	Uncharacterized protein
osa-miR160c-3p	up	LOC_Os12g01950	down	Uncharacterized protein
osa-miR160c-3p	up	LOC_Os06g15330	down	Zinc finger protein CONSTANS-LIKE 16
osa-miR160d-3p	up	LOC_Os11g06020	down	BEL1-like homeodomain protein 1
osa-miR160d-3p	up	LOC_Os11g45890	down	Uncharacterized protein
osa-miR160d-3p	up	LOC_Os09g17329	down	Retrovirus-related Pol polyprotein from transposon TNT 1-94
osa-miR164a	up	LOC_Os05g26990	down	Phospholipid-transporting ATPase 2
osa-miR164a	up	LOC_Os07g42450	down	40S ribosomal protein SA
osa-miR164a	up	LOC_Os03g64140	down	Uncharacterized protein
osa-miR164a	up	LOC_Os01g45530	down	Single-stranded DNA-binding protein, mitochondrial

osa-miR164a	up	LOC_Os04g37619	down	Zeaxanthin epoxidase, chloroplastic
osa-miR164a	up	LOC_Os06g38340	down	Probable LRR receptor-like serine/threonine-protein kinase
osa-miR164a	up	LOC_Os02g38160	down	Beta-1,4-mannosyl-glycoprotein 4-beta-N-acetylglucosaminyltransferase
osa-miR164a	up	LOC_Os07g48510	down	Thioredoxin-like 1-1, chloroplastic
osa-miR164a	up	LOC_Os12g43410	down	Thaumatin-like protein
osa-miR164a	up	LOC_Os02g57280	down	Brown planthopper-induced resistance protein 5
osa-miR164a	up	LOC_Os05g38880	down	Uncharacterized protein
osa-miR164a	up	LOC_Os01g70460	down	Uncharacterized protein
osa-miR164a	up	LOC_Os01g09252	down	Zinc finger CCCH domain-containing protein 17 (abiotic stresses)
osa-miR164a	up	LOC_Os11g40970	down	Probable LRR receptor-like serine/threonine-protein kinase
osa-miR164b	up	LOC_Os05g26990	down	Phospholipid-transporting ATPase 2
osa-miR164b	up	LOC_Os07g42450	down	40S ribosomal protein SA
osa-miR164b	up	LOC_Os03g64140	down	Uncharacterized protein
osa-miR164b	up	LOC_Os01g45530	down	Single-stranded DNA-binding protein, mitochondrial
osa-miR164b	up	LOC_Os04g37619	down	Zeaxanthin epoxidase, chloroplastic
osa-miR164b	up	LOC_Os06g38340	down	Probable LRR receptor-like serine/threonine-protein kinase
osa-miR164b	up	LOC_Os02g38160	down	Beta-1,4-mannosyl-glycoprotein 4-beta-N-acetylglucosaminyltransferase
osa-miR164b	up	LOC_Os07g48510	down	Thioredoxin-like 1-1, chloroplastic
osa-miR164b	up	LOC_Os12g43410	down	Thaumatin-like protein
osa-miR164b	up	LOC_Os02g57280	down	Brown planthopper-induced resistance protein 4
osa-miR164b	up	LOC_Os05g38880	down	Uncharacterized protein
osa-miR164b	up	LOC_Os01g70460	down	Uncharacterized protein
osa-miR164b	up	LOC_Os11g40970	down	Probable LRR receptor-like serine/threonine-protein kinase
osa-miR164f	up	LOC_Os05g26990	down	Phospholipid-transporting ATPase 2
osa-miR164f	up	LOC_Os07g42450	down	40S ribosomal protein SA
osa-miR164f	up	LOC_Os03g64140	down	Uncharacterized protein
osa-miR164f	up	LOC_Os01g45530	down	Single-stranded DNA-binding protein, mitochondrial

osa-miR164f	up	LOC_Os04g37619	down	Zeaxanthin epoxidase, chloroplastic
osa-miR164f	up	LOC_Os06g38340	down	Probable LRR receptor-like serine/threonine-protein kinase
osa-miR164f	up	LOC_Os02g38160	down	Beta-1,4-mannosyl-glycoprotein 4-beta-N-acetylglucosaminyltransferase
osa-miR164f	up	LOC_Os07g48510	down	Thioredoxin-like 1-1, chloroplastic
osa-miR164f	up	LOC_Os12g43410	down	Thaumatin-like protein
osa-miR164f	up	LOC_Os02g57280	down	Brown planthopper-induced resistance protein 3
osa-miR164f	up	LOC_Os05g38880	down	Uncharacterized protein
osa-miR164f	up	LOC_Os01g70460	down	Uncharacterized protein
osa-miR164f	up	LOC_Os11g40970	down	Probable LRR receptor-like serine/threonine-protein kinase
osa-miR166b-5p	up	LOC_Os01g07590	down	universal stress protein domain containing protein
osa-miR166b-5p	up	LOC_Os11g07910	down	Transmembrane 9 superfamily member 3
osa-miR166b-5p	up	LOC_Os01g74540	down	GATA zinc finger domain containing protein
osa-miR166b-5p	up	LOC_Os01g42790	down	Zingipain-2
osa-miR166b-5p	up	LOC_Os08g37700	down	33 kDa ribonucleoprotein, chloroplastic
osa-miR166b-5p	up	LOC_Os03g52640	down	Uncharacterized protein
osa-miR166d-5p	up	LOC_Os01g07590	down	universal stress protein domain containing protein
osa-miR166d-5p	up	LOC_Os01g74540	down	GATA zinc finger domain containing protein
osa-miR166d-5p	up	LOC_Os01g42790	down	Zingipain-2
osa-miR166d-5p	up	LOC_Os08g37700	down	33 kDa ribonucleoprotein, chloroplastic
osa-miR166d-5p	up	LOC_Os03g52640	down	Uncharacterized protein
osa-miR167a-3p	up	LOC_Os01g71720	down	Amino-acid permease BAT1
osa-miR167a-3p	up	LOC_Os03g40550	down	Fructokinase-2
osa-miR167a-3p	up	LOC_Os09g04290	down	Uncharacterized protein
osa-miR167a-3p	up	LOC_Os08g32930	down	Putative ribonuclease H protein
osa-miR167a-3p	up	LOC_Os01g71630	down	Uncharacterized protein
osa-miR167a-5p	down	LOC_Os04g31040	up	Violaxanthin de-epoxidase, chloroplastic
osa-miR167a-5p	down	LOC_Os03g18740	up	Sex determination protein tasselseed-2

osa-miR167a-5p	down	LOC_Os06g03930	up	Cytochrome P450 704C1
osa-miR167a-5p	down	LOC_Os03g10950	up	Probable protein phosphatase 2C 29
osa-miR167a-5p	down	LOC_Os01g38180	up	Peptidyl-prolyl cis-trans isomerase FKBP62
osa-miR167a-5p	down	LOC_Os04g45460	up	Cysteine-rich repeat secretory protein 15
osa-miR167c-5p	down	LOC_Os04g31040	up	Violaxanthin de-epoxidase, chloroplastic
osa-miR167c-5p	down	LOC_Os03g18740	up	Sex determination protein tasselseed-2
osa-miR167c-5p	down	LOC_Os06g03930	up	Cytochrome P450 704C1
osa-miR167c-5p	down	LOC_Os03g10950	up	Probable protein phosphatase 2C 29
osa-miR167c-5p	down	LOC_Os04g45460	up	Cysteine-rich repeat secretory protein 15
osa-miR167e-3p	up	LOC_Os02g03330	down	Uncharacterized protein
osa-miR167e-3p	up	LOC_Os01g50930	down	Uncharacterized protein
osa-miR167e-3p	up	LOC_Os03g52460	down	Glucose-1-phosphate adenylyltransferase large subunit 1, chloroplastic
osa-miR167e-3p	up	LOC_Os07g11280	down	Pentatricopeptide repeat-containing protein
osa-miR167e-3p	up	LOC_Os04g45710	down	Uncharacterized protein
osa-miR167e-3p	up	LOC_Os08g32930	down	Putative ribonuclease H protein
osa-miR167e-5p	down	LOC_Os04g31040	up	Violaxanthin de-epoxidase, chloroplastic
osa-miR167e-5p	down	LOC_Os03g18740	up	Sex determination protein tasselseed-2
osa-miR167e-5p	down	LOC_Os06g03930	up	Cytochrome P450 704C1
osa-miR167e-5p	down	LOC_Os03g10950	up	Probable protein phosphatase 2C 29
osa-miR167e-5p	down	LOC_Os04g45460	up	Cysteine-rich repeat secretory protein 15
osa-miR167h-3p	up	LOC_Os12g08830	down	PsbP domain-containing protein 4, chloroplastic
osa-miR167h-3p	up	LOC_Os06g22440	down	Jasmonate O-methyltransferase
osa-miR167h-3p	up	LOC_Os08g25050	down	Uncharacterized protein
osa-miR167h-3p	up	LOC_Os11g27730	down	indole-2-monooxygenase
osa-miR167i-3p	up	LOC_Os02g03330	down	Uncharacterized protein
osa-miR167i-3p	up	LOC_Os01g50930	down	Uncharacterized protein
osa-miR167i-3p	up	LOC_Os03g52460	down	Glucose-1-phosphate adenylyltransferase large subunit 1, chloroplastic

osa-miR167i-3p	up	LOC_Os07g11280	down	Pentatricopeptide repeat-containing protein
osa-miR167i-3p	up	LOC_Os04g45710	down	Uncharacterized protein
osa-miR167i-3p	up	LOC_Os08g32930	down	Putative ribonuclease H protein
osa-miR167i-5p	down	LOC_Os04g31040	up	Violaxanthin de-epoxidase, chloroplastic
osa-miR167i-5p	down	LOC_Os03g18740	up	Sex determination protein tasselseed-2
osa-miR167i-5p	down	LOC_Os06g03930	up	Cytochrome P450 704C1
osa-miR167i-5p	down	LOC_Os03g10950	up	Probable protein phosphatase 2C 29
osa-miR167i-5p	down	LOC_Os04g45460	up	Cysteine-rich repeat secretory protein 15
osa-miR171c-3p	up	LOC_Os03g59774	down	Uncharacterized protein
osa-miR171c-3p	up	LOC_Os06g15330	down	Zinc finger protein CONSTANS-LIKE 16
osa-miR171c-3p	up	LOC_Os06g15330	down	Zinc finger protein CONSTANS-LIKE 16
osa-miR171c-3p	up	LOC_Os09g16950	down	L-type lectin-domain containing receptor kinase IX.1
osa-miR171c-3p	up	LOC_Os12g29330	down	NAC domain-containing protein 29
osa-miR171c-3p	up	LOC_Os12g43640	down	Receptor-like protein kinase HAIKU2
osa-miR171c-3p	up	LOC_Os01g48950	down	Uncharacterized protein
osa-miR171c-3p	up	LOC_Os03g52640	down	Uncharacterized protein
osa-miR172c	up	LOC_Os12g16540	down	Wall-associated receptor kinase 3
osa-miR172c	up	LOC_Os01g50930	down	Uncharacterized protein
osa-miR172c	up	LOC_Os01g44980	down	Peptide deformylase 1B, chloroplastic
osa-miR172c	up	LOC_Os01g53020	down	heat shock protein DnaJ
osa-miR172c	up	LOC_Os05g21180	down	Phosphatidylinositol:ceramide inositolphosphotransferase
osa-miR172c	up	LOC_Os06g34450	down	E3 ubiquitin-protein ligase
osa-miR172c	up	LOC_Os12g39120	down	Probable protein phosphatase 2C 78
osa-miR172c	up	LOC_Os09g27734	down	Uncharacterized protein
osa-miR172c	up	LOC_Os06g40640	down	Fructose-bisphosphate aldolase, cytoplasmic isozyme 1
osa-miR172c	up	LOC_Os11g42030	down	Uncharacterized protein
osa-miR172d-3p	up	LOC_Os12g16540	down	Wall-associated receptor kinase 3

osa-miR172d-3p	up	LOC_Os01g50930	down	Uncharacterized protein
osa-miR172d-3p	up	LOC_Os01g44980	down	Peptide deformylase 1B, chloroplastic
osa-miR172d-3p	up	LOC_Os01g53020	down	heat shock protein DnaJ
osa-miR172d-3p	up	LOC_Os05g21180	down	Phosphatidylinositol:ceramide inositolphosphotransferase
osa-miR172d-3p	up	LOC_Os06g34450	down	E3 ubiquitin-protein ligase
osa-miR172d-3p	up	LOC_Os12g39120	down	Probable protein phosphatase 2C 78
osa-miR172d-3p	up	LOC_Os02g57280	down	Brown planthopper-induced resistance protein 2
osa-miR172d-3p	up	LOC_Os07g08340	down	Pyruvate kinase isozyme A, chloroplastic
osa-miR172d-3p	up	LOC_Os09g27734	down	Uncharacterized protein
osa-miR172d-3p	up	LOC_Os06g40640	down	Fructose-bisphosphate aldolase, cytoplasmic isozyme 1
osa-miR172d-3p	up	LOC_Os11g42030	down	Uncharacterized protein
osa-miR172d-5p	up	LOC_Os02g50470	down	Uncharacterized protein
osa-miR172d-5p	up	LOC_Os12g14840	down	Uncharacterized protein
osa-miR1861h	up	LOC_Os03g32160	down	calmodulin binding protein
osa-miR1861h	up	LOC_Os04g43770	down	Uncharacterized protein
osa-miR1861h	up	LOC_Os11g13420	down	RNA binding protein
osa-miR1861h	up	LOC_Os01g02780	down	Probable receptor-like protein kinase
osa-miR1861h	up	LOC_Os08g37700	down	33 kDa ribonucleoprotein, chloroplastic
osa-miR1861j	up	LOC_Os03g32160	down	calmodulin binding protein
osa-miR1861j	up	LOC_Os04g43770	down	Uncharacterized protein
osa-miR1861j	up	LOC_Os11g13420	down	RNA binding protein
osa-miR1861j	up	LOC_Os01g02780	down	Probable receptor-like protein kinase
osa-miR1861j	up	LOC_Os08g37700	down	33 kDa ribonucleoprotein, chloroplastic
osa-miR1863b.2	down	LOC_Os01g01660	up	Isoflavone reductase homolog IRL
osa-miR1863b.2	down	LOC_Os07g46670	up	Protein early responsive to dehydration 15
osa-miR1870-5p	up	LOC_Os03g46200	down	Uncharacterized N-acetyltransferase p20
osa-miR1870-5p	up	LOC_Os10g33210	down	Peptide transporter PTR3-A

osa-miR1870-5p	up	LOC_Os06g36040	down	Uncharacterized protein
osa-miR1870-5p	up	LOC_Os01g35330	down	circumsporozoite protein precursor, putative, expressed
osa-miR1870-5p	up	LOC_Os08g08130	down	Glutathione S-transferase T3
osa-miR1870-5p	up	LOC_Os12g07180	down	Glutathione S-transferase T3
osa-miR1870-5p	up	LOC_Os11g42030	down	Uncharacterized protein
osa-miR1870-5p	up	LOC_Os02g40190	down	LRR receptor-like serine/threonine-protein kinase EFR
osa-miR1883a	up	LOC_Os07g39780	down	SUMO-activating enzyme subunit 2
osa-miR1883a	up	LOC_Os03g40550	down	Fructokinase-2
osa-miR1883a	up	LOC_Os03g46070	down	Zeamatin
osa-miR1883a	up	LOC_Os11g07980	down	Ankyrin repeat domain-containing protein 6
osa-miR1883a	up	LOC_Os10g40030	down	Short-chain dehydrogenase TIC 32, chloroplastic
osa-miR1883a	up	LOC_Os03g52640	down	Uncharacterized protein
osa-miR2871a-5p	up	LOC_Os06g11800	down	Annexin D1
osa-miR2871a-5p	up	LOC_Os07g33480	down	Cytochrome P450 716B1
osa-miR396c-3p	up	LOC_Os03g46200	down	Uncharacterized N-acetyltransferase p20
osa-miR396c-3p	up	LOC_Os03g11670	down	Pentatricopeptide repeat-containing protein , chloroplastic
osa-miR396c-3p	up	LOC_Os01g12400	down	G-type lectin S-receptor-like serine/threonine-protein kinase
osa-miR396c-3p	up	LOC_Os09g06740	down	E3 ubiquitin-protein ligase SDIR1
osa-miR396c-3p	up	LOC_Os06g11150	down	Uncharacterized protein
osa-miR396c-3p	up	LOC_Os07g32590	down	Methionine aminopeptidase 1B, chloroplastic
osa-miR396c-3p	up	LOC_Os04g21820	down	Wall-associated receptor kinase 5
osa-miR396c-3p	up	LOC_Os07g30020	down	Multiple C2 and transmembrane domain-containing protein 1
osa-miR444a-5p	up	LOC_Os08g41990	down	Glutamate-1-semialdehyde 2,1-aminomutase, chloroplastic
osa-miR444a-5p	up	LOC_Os06g50300	down	Endoplasmin homolog
osa-miR444a-5p	up	LOC_Os06g05450	down	Uncharacterized protein
osa-miR444a-5p	up	LOC_Os03g40550	down	Fructokinase-2
osa-miR444a-5p	up	LOC_Os03g27120	down	Metacaspase-1

osa-miR444a-5p	up	LOC_Os02g57280	down	Brown planthopper-induced resistance protein 1
osa-miR444a-5p	up	LOC_Os06g40640	down	Fructose-bisphosphate aldolase, cytoplasmic isozyme 1
osa-miR444a-5p	up	LOC_Os11g38330	down	Uncharacterized protein
osa-miR444a-5p	up	LOC_Os12g14540	down	Uncharacterized protein
osa-miR444c.2	up	LOC_Os07g03200	down	Phytosulfokines 4
osa-miR444c.2	up	LOC_Os01g02400	down	Probable receptor-like protein kinase At5g39020
osa-miR444c.2	up	LOC_Os10g42700	down	CS domain containing protein
osa-miR444c.2	up	LOC_Os08g15460	down	Putative cyclic nucleotide-gated ion channel 15
osa-miR444c.2	up	LOC_Os04g43770	down	Uncharacterized protein
osa-miR444c.2	up	LOC_Os01g11160	down	Cationic amino acid transporter 5
osa-miR444c.2	up	LOC_Os07g14740	down	harpin-induced protein 4 domain containing protein
osa-miR444d.2	up	LOC_Os07g03200	down	Phytosulfokines 4
osa-miR444d.2	up	LOC_Os01g02400	down	Probable receptor-like protein kinase
osa-miR444d.2	up	LOC_Os10g42700	down	CS domain containing protein
osa-miR444d.2	up	LOC_Os08g15460	down	Putative cyclic nucleotide-gated ion channel 15
osa-miR444d.2	up	LOC_Os06g22440	down	Jasmonate O-methyltransferase
osa-miR444d.2	up	LOC_Os01g11160	down	Cationic amino acid transporter 5
osa-miR444d.2	up	LOC_Os07g14740	down	harpin-induced protein 3 domain containing protein
osa-miR444d.2	up	LOC_Os02g40200	down	Probable LRR receptor-like serine/threonine-protein kinase
osa-miR444e	up	LOC_Os07g03200	down	Phytosulfokines 4
osa-miR444e	up	LOC_Os01g02400	down	Probable receptor-like protein kinase
osa-miR444e	up	LOC_Os10g42700	down	CS domain containing protein
osa-miR444e	up	LOC_Os08g15460	down	Putative cyclic nucleotide-gated ion channel 15
osa-miR444e	up	LOC_Os06g22440	down	Jasmonate O-methyltransferase
osa-miR444e	up	LOC_Os01g11160	down	Cationic amino acid transporter 5
osa-miR444e	up	LOC_Os07g14740	down	harpin-induced protein 2 domain containing protein
osa-miR444e	up	LOC_Os02g40200	down	Probable LRR receptor-like serine/threonine-protein kinase

osa-miR444f	up	LOC_Os07g03200	down	Phytosulfokines 4
osa-miR444f	up	LOC_Os01g02400	down	Probable receptor-like protein kinase
osa-miR444f	up	LOC_Os10g42700	down	CS domain containing protein
osa-miR444f	up	LOC_Os04g43770	down	Uncharacterized protein
osa-miR444f	up	LOC_Os01g11160	down	Cationic amino acid transporter 5
osa-miR444f	up	LOC_Os07g14740	down	harpin-induced protein 1 domain containing protein
osa-miR444f	up	LOC_Os02g40200	down	Probable LRR receptor-like serine/threonine-protein kinase
osa-miR5072	up	LOC_Os02g33020	down	Heme-binding-like protein , chloroplastic
osa-miR5072	up	LOC_Os08g26230	down	Uncharacterized protein
osa-miR5072	up	LOC_Os08g32930	down	Putative ribonuclease H protein
osa-miR5072	up	LOC_Os01g73450	down	Uridylate kinase
osa-miR5072	up	LOC_Os10g04570	down	Putative disease resistance protein RGA4
osa-miR5072	up	LOC_Os10g27340	down	Prolyl 4-hydroxylase subunit alpha-1
osa-miR5072	up	LOC_Os12g16200	down	Glutathione synthetase, chloroplastic
osa-miR5072	up	LOC_Os01g06876	down	Receptor-like protein 12
osa-miR812a	up	LOC_Os02g38920	down	Glyceraldehyde-3-phosphate dehydrogenase, cytosolic
osa-miR812a	up	LOC_Os07g48510	down	Thioredoxin-like 1-1, chloroplastic
osa-miR812b	up	LOC_Os02g38920	down	Glyceraldehyde-3-phosphate dehydrogenase, cytosolic
osa-miR812b	up	LOC_Os07g48510	down	Thioredoxin-like 1-1, chloroplastic
osa-miR812c	up	LOC_Os02g38920	down	Glyceraldehyde-3-phosphate dehydrogenase, cytosolic
osa-miR812c	up	LOC_Os07g48510	down	Thioredoxin-like 1-1, chloroplastic
osa-miR812d	up	LOC_Os02g38920	down	Glyceraldehyde-3-phosphate dehydrogenase, cytosolic
osa-miR812d	up	LOC_Os07g48510	down	Thioredoxin-like 1-1, chloroplastic
osa-miR812e	up	LOC_Os02g38920	down	Glyceraldehyde-3-phosphate dehydrogenase, cytosolic
osa-miR812e	up	LOC_Os07g48510	down	Thioredoxin-like 1-1, chloroplastic
osa-miR812h	up	LOC_Os06g50300	down	Endoplasmin homolog
osa-miR812h	up	LOC_Os11g42970	down	CASP-like protein

osa-miR812h	up	LOC_Os02g03370	down	Uncharacterized protein
osa-miR812h	up	LOC_Os08g26230	down	Uncharacterized protein
osa-miR812h	up	LOC_Os05g38880	down	Uncharacterized protein
osa-miR812i	up	LOC_Os06g50300	down	Endoplasmin homolog
osa-miR812i	up	LOC_Os11g42970	down	CASP-like protein
osa-miR812i	up	LOC_Os02g03370	down	Uncharacterized protein
osa-miR812i	up	LOC_Os08g26230	down	Uncharacterized protein
osa-miR812i	up	LOC_Os05g38880	down	Uncharacterized protein
osa-miR812j	up	LOC_Os06g50300	down	Endoplasmin homolog
osa-miR812j	up	LOC_Os11g42970	down	CASP-like protein
osa-miR812j	up	LOC_Os02g03370	down	Uncharacterized protein
osa-miR812j	up	LOC_Os08g26230	down	Uncharacterized protein
osa-miR812j	up	LOC_Os05g38880	down	Uncharacterized protein
