# Supplementary material for: Integrative Analysis of the microRNAome and Transcriptome Illuminates the Response of Susceptible Rice Plants to Rice Stripe Virus
Source: PLoS One. 2016 Jan 22;11(1):e0146946. doi: 10.1371/journal.pone.0146946 (PMC4723043; doi:10.1371/journal.pone.0146946)
Supplement: S7 Table — (PDF) [file pone.0146946.s007.pdf]

S7 Table. The relative expression levels of mRNAs evaluated by qPCR assay

Genes	RI		CK		p-value	Fold change (RI/CK)
	CT values	Std. Dev.	CT values	Std. Dev.		
LOC_Os09g19280 (RPM1)	29.619	0.010	26.530	0.046	0.000	0.072
LOC_Os02g40190 (EFR)	22.516	0.069	21.014	0.327	0.002	0.230
LOC_Os08g34740 (SGT1)	23.902	0.076	22.638	0.563	0.025	0.271
LOC_Os02g33180 (RAR1)	24.142	0.035	22.625	0.237	0.001	0.227
LOC_Os03g18850 (PR1)	30.289	0.237	26.541	0.237	0.000	0.048
LOC_Os06g48590 (MPK4)	28.364	0.124	27.598	0.524	0.000	0.382
LOC_Os03g55164 (WRKY33)	28.132	0.255	26.887	0.356	0.005	0.274
LOC_Os03g49640 (MEKK1)	22.630	0.563	21.007	0.537	0.000	0.211
LOC_Os06g05520 (MEK1)	28.549	0.236	27.222	0.369	0.000	0.259
LOC_Os09g29840 (HSP90)	28.376	0.512	27.593	0.026	0.001	0.378
LOC_Os11g40970 (Probable LRR receptor-like serine/threonine-protein kinase	28.770	0.357	26.518	0.365	0.003	0.137
LOC_Os02g40200 (Probable LRR receptor-like serine/threonine-protein kinase)	30.263	0.079	26.581	0.537	0.000	0.051
LOC_Os01g71340 (Glucan endo-1,3-beta-glucosidase)	24.098	0.448	22.675	0.057	0.003	0.229
LOC_Os07g35680 (Cysteine-rich receptor-like protein kinase 8)	28.454	0.068	27.220	0.037	0.001	0.261
LOC_Os02g57280 (Brown planthopper-induced resistance protein 6)	22.545	0.359	20.959	0.279	0.002	0.205
LOC_Os01g53020 (heat shock protein DnaJ)	29.770	0.466	26.518	0.369	0.002	0.064
LOC_Os04g21820 (Wall-associated receptor kinase 5)	28.590	0.165	27.531	0.560	0.009	0.295
LOC_Os10g04570 (Putative disease resistance protein RGA4)	23.194	0.560	21.538	0.579	0.005	0.195
