# Supplementary material for: Integrative Analysis of the microRNAome and Transcriptome Illuminates the Response of Susceptible Rice Plants to Rice Stripe Virus
Source: PLoS One. 2016 Jan 22;11(1):e0146946. doi: 10.1371/journal.pone.0146946 (PMC4723043; doi:10.1371/journal.pone.0146946)
Supplement: S8 Table — (PDF) [file pone.0146946.s008.pdf]

S8 Table. The different expression levels of AP2/DREBP/EREBP/NAC-like transcript factors after RSV infection (Fold change≥2 and P<0.05)

Gene ID	Average FPKM values		p-value	Fold change (RI/CK)	Gene description
	RI	CK			
LOC_Os01g49830	6.585 ± 0.455	16.588 ± 1.448	0.0062	0.397	AP2/ERF and B3 domain-containing protein Os01g0693400
LOC_Os05g47650	4.351 ± 0.065	19.795 ± 2.507	0.0129	0.22	AP2/ERF and B3 domain-containing protein Os05g0549800
LOC_Os09g35030	29.807 ± 2.122	0.718 ± 0.399	0.002	41.507	Dehydration-responsive element-binding protein 1A
LOC_Os09g35010	109.578±11.576	2.746 ± 1.162	0.0054	39.911	Dehydration-responsive element-binding protein 1B
LOC_Os04g48350	19.079 ± 2.598	0.750 ± 0.151	0.0097	25.434	Dehydration-responsive element-binding protein 1E
LOC_Os09g35020	10.362 ± 1.623	0.867 ± 1.125	0.0037	11.95	Dehydration-responsive element-binding protein 1H
LOC_Os09g28440	27.766 ± 3.790	0.427 ± 0.167	0.0094	64.963	Ethylene-responsive transcription factor ERF109
LOC_Os04g52090	101.857 ± 9.393	11.384 ± 3.227	0.0025	8.948	Ethylene-responsive transcription factor 4
LOC_Os05g41780	375.156± 39.887	59.612 ± 4.436	0.0073	6.293	Ethylene-responsive transcription factor 4
LOC_Os04g46440	13.942 ± 1.851	3.479 ± 0.893	0.0063	4.007	Ethylene-responsive transcription factor ERF038
LOC_Os01g58420	31.267 ± 3.130	8.477 ± 2.614	0.0016	3.688	Ethylene-responsive transcription factor 4
LOC_Os03g08500	170.069 ± 8.295	64.924 ±13.471	0.0017	2.62	Ethylene-responsive transcription factor ERF073
LOC_Os09g11480	16.177 ± 1.032	51.889 ± 6.019	0.012	0.312	Ethylene-responsive transcription factor ERF110
LOC_Os05g03040	37.235 ± 7.101	95.716 ± 5.033	0.0011	0.389	Ethylene-responsive transcription factor RAP2-7
LOC_Os11g05614	10.215 ± 0.704	2.097 ± 0.657	0.0003	4.872	NAC domain-containing protein 90
LOC_Os01g48446	17.712 ± 1.693	4.216 ± 0.573	0.0041	4.201	NAC domain-containing protein 68
LOC_Os03g60080	293.077±43.510	88.794 ± 5.751	0.0204	3.301	NAC domain-containing protein 67
LOC_Os05g34830	15.105 ± 0.647	5.993 ± 1.367	0.0041	2.52	NAC domain-containing protein 48
LOC_Os07g12340	43.132 ± 0.605	21.039 ± 1.617	0.0009	2.05	NAC domain-containing protein 67
LOC_Os10g21560	5.732 ± 0.390	11.675 ± 1.101	0.01	0.491	NAC domain-containing protein 78
LOC_Os12g29330	20.463 ± 1.491	46.874 ± 4.155	0.0066	0.437	NAC domain-containing protein 29
LOC_Os09g33490	4.615 ± 0.257	10.613 ± 1.220	0.0167	0.435	NAC domain-containing protein 18
LOC_Os06g51070	31.221 ± 5.318	83.764±7.554	0.002	0.373	Putative NAC domain-containing protein 94
LOC_Os01g70110	5.157 ± 0.631	14.946±0.516	0.0001	0.345	NAC domain-containing protein 18
